# Precise Semi-Experimental Equilibrium (*r*_*e*_^SE^) Structure of Pyridine from 32 Isotopologues: Accurate Assessment of the Effect of Nitrogen-Atom Substitution in Aromatic Rings

**DOI:** 10.1021/acs.jpca.5c07184

**Published:** 2025-11-07

**Authors:** Maria A. Zdanovskaia, Brian J. Esselman, Samuel M. Kougias, Madeleine G. Atwood, Gregory H. Jones, John F. Stanton, R. Claude Woods, Robert J. McMahon

**Affiliations:** † Department of Chemistry, 5228 University of Wisconsin−Madison, Madison, Wisconsin 53706, United States; ‡ Quantum Theory Project, Departments of Physics and Chemistry, 3463 University of Florida, Gainesville, Florida 32611, United States

## Abstract

Millimeter-wave rotational spectra of 32 pyridine isotopologues have been measured within the 80–750 GHz frequency range. The rotational constants resulting from analysis of these spectra, corrected for the effects of vibration–rotation interactions and electron-mass distributions using CCSD­(T)/cc-pCVTZ calculations, enable the determination of a highly precise semiexperimental equilibrium structure (*r*_*e*_^SE^) of pyridine. The structural parameters of pyridine are in generally good agreement with those of previously published *r*_*e*_^SE^ structures, but the precision achieved here is substantially improved: bond lengths are determined to ±0.0002 Å and bond angles are determined to ±0.020° (2σ uncertainties). Such precision approaches the limit of semiexperimental equilibrium structure determination using a contemporary methodology. The current investigation provides critical structural data that enables a detailed analysis of the effects of nitrogen-atom substitution in prototypical aromatic molecules. Only through comparisons among recently determined, highly precise structures for benzene, pyridine, pyridazine, and pyrimidine are the subtle structural effects of nitrogen-atom substitution in the aromatic ring experimentally revealed.

## Graphical abstract

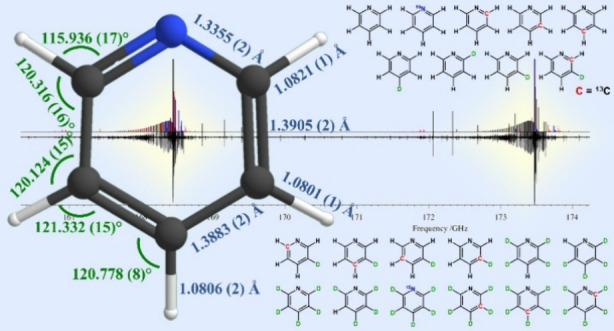

## Introduction

Pyridine (*c*-C_5_H_5_N, *C*_2*v*_, Figure 1) is a prototypical aromatic heterocycle. The nitrogen atom and its electron lone-pair change the structure and reactivity of pyridine, relative to that of benzene, in significant ways. Although comparisons of experimental structural parameters for pyridine and benzene abound, the differences are small and, in many cases, are smaller than the experimental uncertainty of the measurements. With the recent determinations of highly precise semiexperimental equilibrium structures (*r*_*e*_^SE^) of benzene,^1^ pyridazine,^2^ and pyrimidine,^3^ pyridine represents the missing structure needed to enable a systematic comparison among these molecules. In the current study, we determined the gas-phase structure of pyridine by measuring the rotational spectra of 32 pyridine isotopologues. This and other recently determined structures provide the basis for a detailed comparison of the structural changes that occur upon heteroatom substitution (CH with N) in some simple aromatic rings. In terms of spectroscopy, the rotational spectrum of the normal isotopologue of pyridine also remains of fundamental interest in the field of astrochemistry. For decades, pyridine has been considered to be a likely component of the interstellar medium (ISM). Numerous searches, however, failed to provide a successful detection, and the reason for the apparent absence of heterocycles in the ISM is not well understood.^4–8^

**Figure 1:**
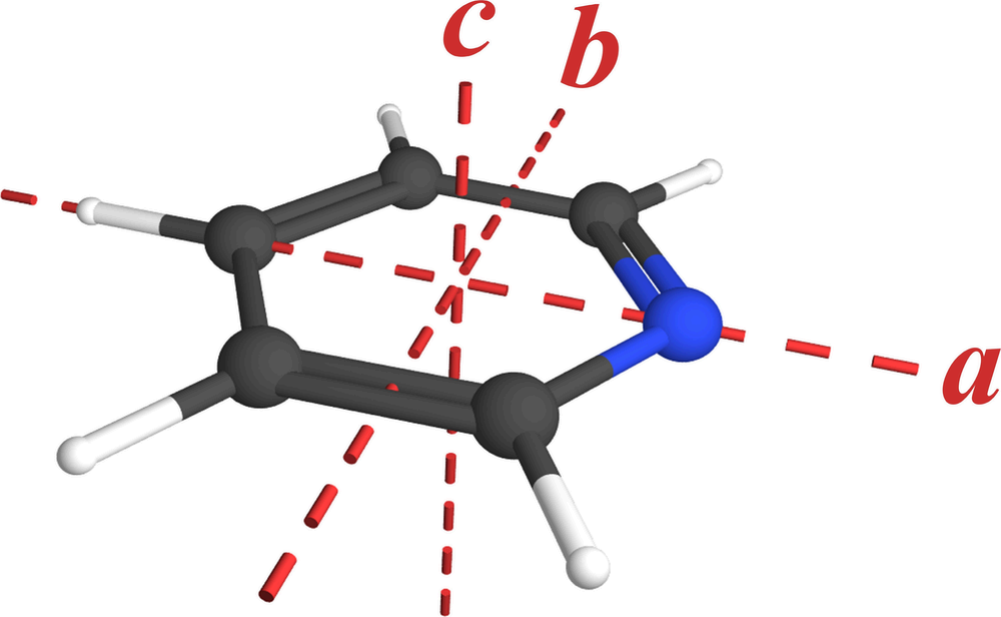
Pyridine structure (*C*_2v_, μ_*a*_ = 2.215 (1) D)^9^ with principal inertial axes.

Historians of science debate whether Koerner or Dewar was the first to propose the structure of pyridine (*ca*. 1868–1870).^10^ The vibrational spectrum of pyridine in the gas and liquid phases has been examined extensively using infrared (IR) and Raman spectroscopies,^11–27^ including multiresonant coherent multidimensional vibrational spectroscopy^28^ and mass-correlated rotational alignment spectroscopy (mass-CRASY).^29^ Various studies have reported the Raman and IR spectra of deuteriated pyridines, in both liquid and vapor phases.^14,15,17–19,30^ Remarkably, the crystal structure of pyridine was not solved until 1981.^31^ It has since been studied under high pressure^32^ and theoretically^33^ to elucidate the origin of the complex crystal structure.

Bak and Rastrup-Andersen measured the microwave spectrum of pyridine in 1953 with an interest in elucidating the structure of the molecule.^34^ McCulloh and Pollnow determined what is now known to be the correct value of the asymmetry parameter (κ = 0.84777), establishing the structure as an oblate *C*_2*v*_-symmetric top.^35,36^ In subsequent works, Bak and Rastrup-Andersen refined the pyridine substitution (*r*_*s*_) structure.^37–39^ So̷rensen *et al.* updated the rotational and quartic centrifugal distortion constants, the nuclear quadrupole coupling constants in the normal and singly heavy-atom substituted isotopologues, and the substitution structure.^9,40^ In 1977, Mata *et al*. addressed the quadrupole coupling in the singly deuteriated pyridines to further improve the substitution structure of pyridine.^41^ Refined values for quadrupole coupling^42^ and centrifugal distortion terms^43^ have subsequently been reported. A temperature-dependent (*r*_*g*_) molecular structure of gaseous pyridine using electron-diffraction, microwave, and infrared data was reported in 1987.^44^ In 2005, millimeter-wave data were used to improve the spectroscopic constants of the vibrational ground state and to examine the vibrationally excited states.^45^ Using the previously published rotational constants, three semiexperimental equilibrium structures (*r*_*e*_^SE^) of pyridine were reported in 2015 by Császár *et al*.,^46^ Piccardo *et al*.,^47,48^ and Penocchio *et al*.^49,50^ The semiexperimental equilibrium structures in all three works used vibration–rotation interaction and electron-mass distribution corrections calculated using density functional theory (DFT). Piccardo *et al*. and Penocchio *et al*. based their structures on eight isotopologues—the minimum number necessary to substitute each atom once. Császár *et al*. based their structure on 10 isotopologues: the minimum set of eight isotopologues along with two multiply substituted isotopologues ([2,3,4,5,6-^2^H] and [4-^13^C, ^15^N]). The planar, *C*_2*v*_ geometry of pyridine consists of ten independent structural parameters (six bond lengths, four bond angles). Due to the planarity of the molecule, each isotopologue provides two independent moments of inertia (*I*_*c*_ – *I*_*a*_ – *I*_*b*_ = 0). Thus, the data sets for the previous analyses (16 or 20 independent moments) are sufficient for complete structure determinations.

## Methods

### Materials and Synthesis

Pyridine, [^15^N]-pyridine, and [2,3,4,5,6-^2^H]-pyridine were obtained commercially and used for analysis without further purification. All these samples were sufficiently pure to observe all of their singly substituted heavy-atom isotopologues at natural abundance. The sample of [2,3,4,5,6-^2^H]-pyridine (pyridine-*d*_5_) was sufficiently *impure* to observe all three pyridine-*d*_4_ isotopologues as minor contaminants.

Various synthetic procedures, along with good fortune, enabled the spectroscopic observation of 16 of the 20 possible deuterium-containing isotopologues of pyridine. The [2-^2^H] and [3-^2^H] isotopologues of pyridine were prepared from commercially available 2-bromopyridine or 3-bromopyridine by lithium-halogen exchange, followed by quenching with D_2_O (Scheme 1). The lithiation chemistry of halopyridines is not necessarily clean, owing to rearrangements and other competing processes.^51,52^ In our hands, the reaction of 2-bromopyridine afforded a sample that contained not only the intended monosubstituted product, [2-^2^H]-pyridine, but also a small amount of two disubstituted products, [2,3-^2^H]- and [2,5-^2^H]-pyridine (Scheme 1a). The reaction of 3-bromopyridine afforded a sample of sufficient quantity and purity of [3-^2^H]-pyridine (Scheme 1b) to observe all singly substituted heavy-atom isotopologues at natural abundance.

**Scheme 1:**
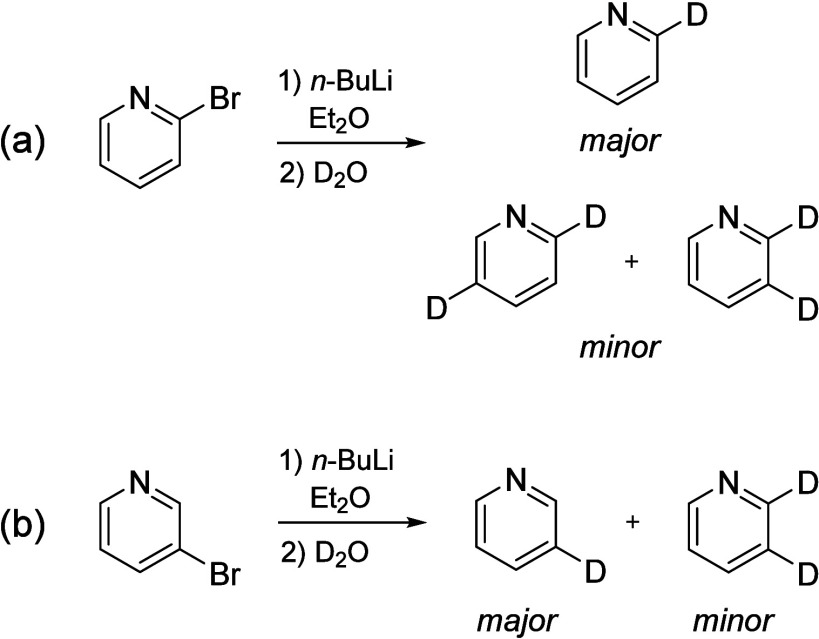
Lithium–Halogen Exchange Reactions, Followed by Quenching with D_2_O, Used to Prepare Deuterium-Containing Isotopologues of Pyridine

Base-catalyzed H/D exchange reactions of pyridine-*h*_5_ with Me_2_SO-*d*_6_ (Scheme 2a) and of pyridine-*d*_5_ with Me_2_SO-*h*_6_ (Scheme 2b) were employed, based upon the methodology of Li *et al*.^53^ Experimental details, a scheme depicting structures of all possible deuterium-containing isotopologues, and commentary concerning the occasionally subtle conventions for naming/numbering the isotopologues are available in the Supporting Information.

**Scheme 2:**
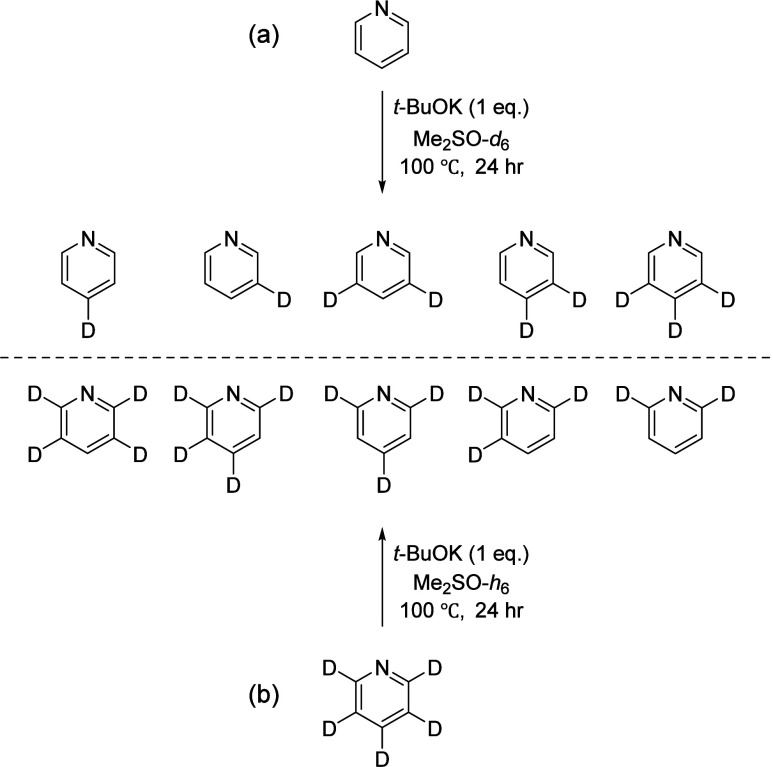
Hydrogen–Deuterium Exchange Reactions Used to Obtain Various Deuterium-Containing Isotopologues of Pyridine

### Spectroscopic Methods

Using an instrument described previously,^54^ continuous broadband spectra were obtained in the 130–230, 235–360, 350–500, and 500–750 GHz frequency regions for the normal isotopologue. Spectra of a reduced frequency range were obtained for the enriched samples of [^15^N]-pyridine (80–134, 155–243, 235–360, and 350–432 GHz) and [2,3,4,5,6-^2^H]-pyridine (130–230 GHz). Spectral segments were subsequently stitched together and analyzed using Kisiel’s Assignment and Analysis of Broadband Spectra (AABS) software.^55,56^ Spectra were obtained automatically at a rate of approximately 55 GHz per day, given the following experimental parameters: 0.6 MHz/s sweep rate, 10 ms time constant, and 50 kHz AM and 500 kHz FM modulation in a tone-burst design.^57^ Subsets of the full frequency range were acquired for the other isotopologue samples based largely on the amount of sample available. Spectroscopic data were analyzed using SPFIT and SPCAT,^58^ as well as PIFORM, AC, PLANM, and other useful helper programs.^59^ We incorporated previously published transitions into the least-squares fits of isotopologues for which such data were available, using the reported experimental uncertainties. Data acquired in this work assumed a uniform 50 kHz frequency measurement uncertainty. All output files from the least-squares fits are provided in Supporting Information.

### Computational Methods

The computational methods employed in this work have been described previously for a number of structure determinations.^1–3,54,60^ A development version of CFOUR^61^ was used for geometry optimizations at the CCSD­(T) level of theory using cc-pCVTZ, cc-pCVQZ, and cc-pCV5Z basis sets. Additional optimizations using CCSDT­(Q)/cc-pVDZ, CCSD­(T)/cc-pVDZ, CCSD­(T)/cc-pCVTZ with the RELATIVISTIC = X2C1E keyword, and SCF/cc-pVTZ with the DBOC = ON keyword comprise the calculations necessary for the “best theoretical estimate” (BTE), which uses the CCSD­(T)/cc-pCV5Z structure as a basis with four corrections:

1. Residual basis set effects beyond cc-pCV5Z.
2. Residual electron correlation effects beyond the CCSD­(T) treatment.
3. Effects of scalar (mass-velocity and Darwin) relativistic effects.
4. The diagonal Born–Oppenheimer correction (DBOC), calculated for the normal isotopic species, C_5_H_5_N.

Details regarding these corrections are provided in the Supporting Information.

The structure computed at CCSD­(T)/cc-pCVTZ was subsequently used for a second-order vibrational perturbation (VPT2) anharmonic frequency calculation by evaluating the cubic force constants using analytical second derivatives at displaced points.^62–64^ The VPT2 calculation was used to obtain anharmonic vibrational frequencies, vibration–rotation interaction constants (*B*_0_ – *B*_*e*_), and quartic and sextic centrifugal distortion terms for each isotopologue. Rotational *g*-tensor calculations computed at CCSD­(T)/cc-pCVTZ provided electron-mass corrections for each isotopologue. Computed, isotopologue-specific centrifugal distortion constants were used in the least-squares fits of spectroscopic data for constants that could not be determined experimentally. The computed vibration–rotation interaction and electron-mass corrections were combined with the averaged determinable constants (*B*_0_^″^) to obtain the semiexperimental equilibrium determinable constants (*B*_*e*_^″^). These equilibrium constants, after conversion to the corresponding moments of inertia, were used by the *xrefit* module of CFOUR to determine the *r*_*e*_^SE^ structural parameters *via* a nonlinear least-squares fit, using the Levenberg–Marquardt algorithm. The *xrefiteration* script was used to examine the impact that incorporation of data from each isotopologue had on the final semiexperimental equilibrium structure parameters.^2^

## Results and Discussion

### Analysis of Rotational Spectra

Initially, we fit pyridine and its isotopologues in the III*^r^* representation, but the A-reduction least-squares fit exhibited a breakdown for the normal and other isotopologues, as has been observed with other molecules.^65–72^ We therefore use the I*^r^* representation for all A- and S-reduction least-squares fits presented in this work. All isotopologues are fit using both Hamiltonian models, enabling the calculation of determinable constants (*A*_0_^″^) from each reduction and comparison of the two sets of determinable constants (*A*_0_^″(*S*)^ *vs* *A*_0_^″(*A*)^) as one measure of the quality of the experimentally determined constants. For isotopologues whose spectroscopic constants were available in the literature, those constants were combined with computed distortion constants to make initial predictions in our spectral region. Over 6,000 transitions were newly measured and least-squares fit for the normal isotopologue of pyridine. The extended range of analyzed *J* and *K* quantum number values enabled the determination of the complete set of quartic and sextic centrifugal distortion constants, several of which have not previously been reported. Similarly, over 1200 transitions were newly measured for each mono-^13^C isotopologue, over 3500 for [^15^N]-pyridine, over 900 for [2-^2^H]-pyridine, and over 1800 for [3-^2^H]-pyridine. Over 600 newly measured transitions were included with those of Bettens and Bauder^73^ in the least-squares fit of [4-^13^C, ^15^N]-pyridine, and over 800 transitions were added to those of Heineking *et al*.^42^ for [4-^2^H]-pyridine. Since the isotopologues noted above have been reported in several previous publications, we provide their spectroscopic constants and data distribution plots in the Supporting Information and focus here on less-studied species. The [3-^2^H]-pyridine sample was sufficiently pure to also analyze all five singly ^13^C-substituted [3-^2^H]-pyridine isotopologues. The spectroscopic constants for these isotopologues have not been previously reported, but we present their data in the Supporting Information in the interest of brevity.

We assigned and measured over 3500 new transitions of [2,3,4,5,6-^2^H]-pyridine, which were combined with previous microwave data, and least-squares fit using a sextic, distorted-rotor Hamiltonian in both the A and S reductions, I*^r^* representation (Table 1). The computed rotational constants are within 1% of the experimentally determined values, and the computed quartic centrifugal distortion terms are within 5% of the experimentally determined values, except *D*_*K*_ (8% difference). The sextic centrifugal distortion terms that were determined in the least-squares fit are newly reported. In the A reduction, computed constants fall within 20% of the values that could be experimentally determined, except Φ_*JK*_ and Φ_*KJ*_ (87% and 37%, respectively). While these discrepancies are somewhat larger than those typically encountered, they do not stand out as unusual levels of agreement. In the S reduction, however, the discrepancies are quite a bit larger. The computed terms differ from the experimentally determined values by 25–330%. Attempting to hold even the term in poorest agreement constant at its computed value, however, results in many transitions being rejected from the data set. Therefore, these terms are included in the least-squares fit. The agreement of the rotational and quartic centrifugal distortion constants, as well as most of the A-reduction sextic terms, lends credence to both the quality of the computation and the meaningful determination of the experimental constants. The origin of discrepancies in the S reduction is not clear. One possibility is that the transition data set is sufficiently broad to contain information about some of the sextic centrifugal distortion constants that are held at their computed values—enough information to affect the fitted constants, but not quite enough to determine an additional sextic term with low uncertainty. The data distribution plot shown in Figure 2 provides a visualization of the quantum number ranges observed. The plot also enables a visual comparison of previously measured transitions to the expanded data set acquired in this work.

**Table 1:**
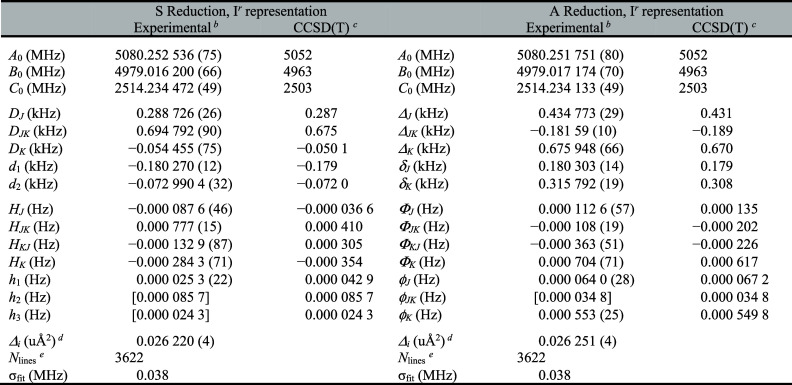
Experimental and Computed Spectroscopic Constants for [2,3,4,5,6-^2^H]-Pyridine^a^

a Values in square brackets held fixed at the computed value [CCSD­(T)/cc-pCVTZ] in the least-squares fit.

b Includes transitions from Spycher *et al*.^74^

c Evaluated using the cc-pCVTZ basis set.

d Inertial defect, Δ_*i*_ = *I*_*c*_ – *I*_*a*_ – *I*_*b*_. Calculated using PLANM.

e Number of independent transitions.

**Figure 2:**
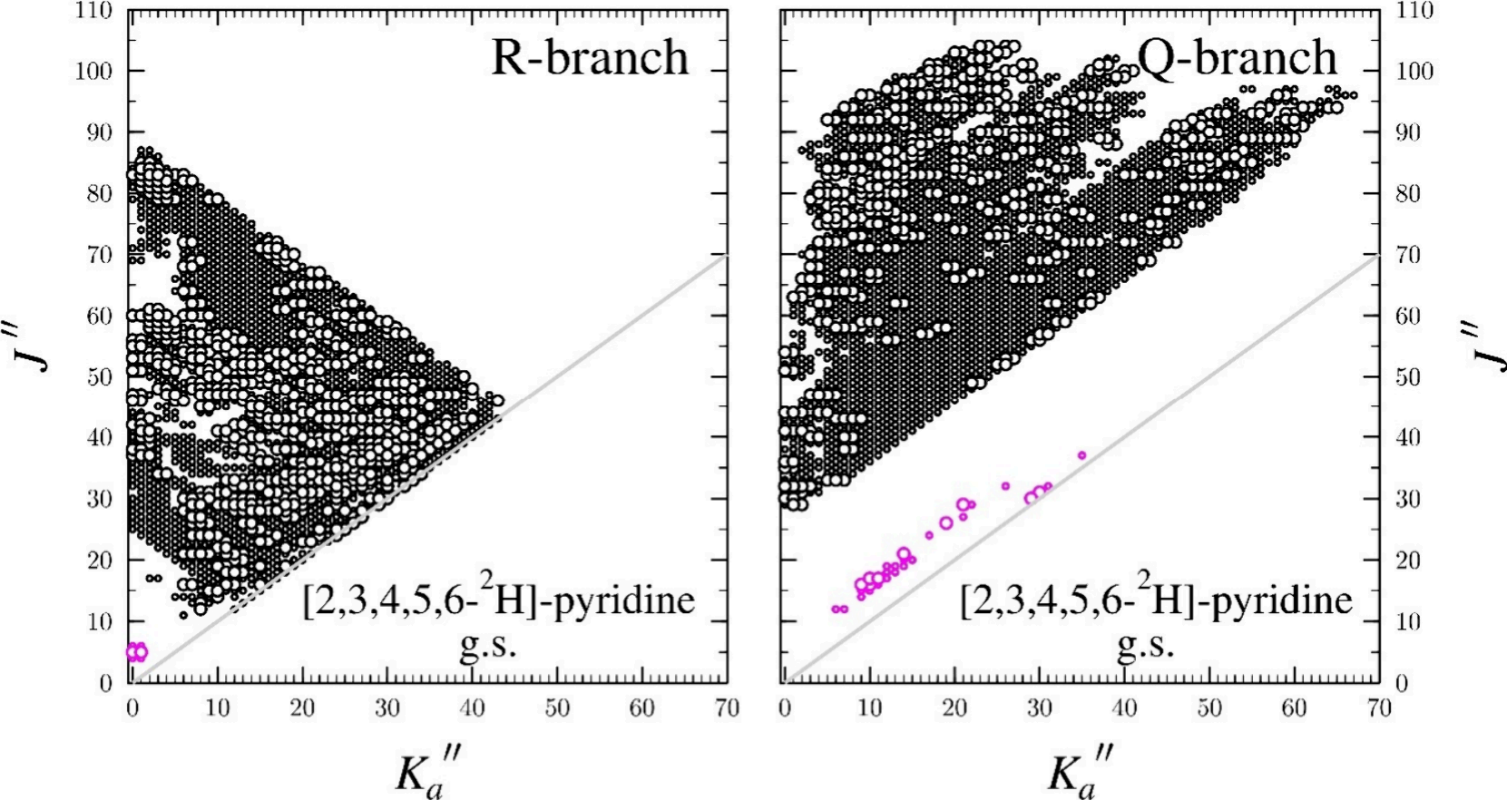
Data distribution plot for the least-squares fit of millimeter-wave spectroscopic data for [2,3,4,5,6-^2^H]-pyridine in the ground vibrational state. Black circles are measurements from the current work, while magenta circles are from previous work.^74^ The size of the outlined circle is proportional to the value of |(*f*_obs._ – *f*_calc._)/δ*f* |, where δ*f* is the frequency measurement uncertainty (50 kHz), and no quotient values are larger than three.

A snippet of the rotational spectrum collected using commercially available pyridine-*d*_5_ is presented in Figure 3. This isotopologue exhibits a typical oblate-top band structure. The sample was sufficiently pure (nominally 95.5%) to observe all singly substituted heavy-atom isotopologues at natural abundance. Fortuitously, the sample was sufficiently *impure* to observe all three pyridine-*d*_4_ isotopologues, as well.

**Figure 3:**
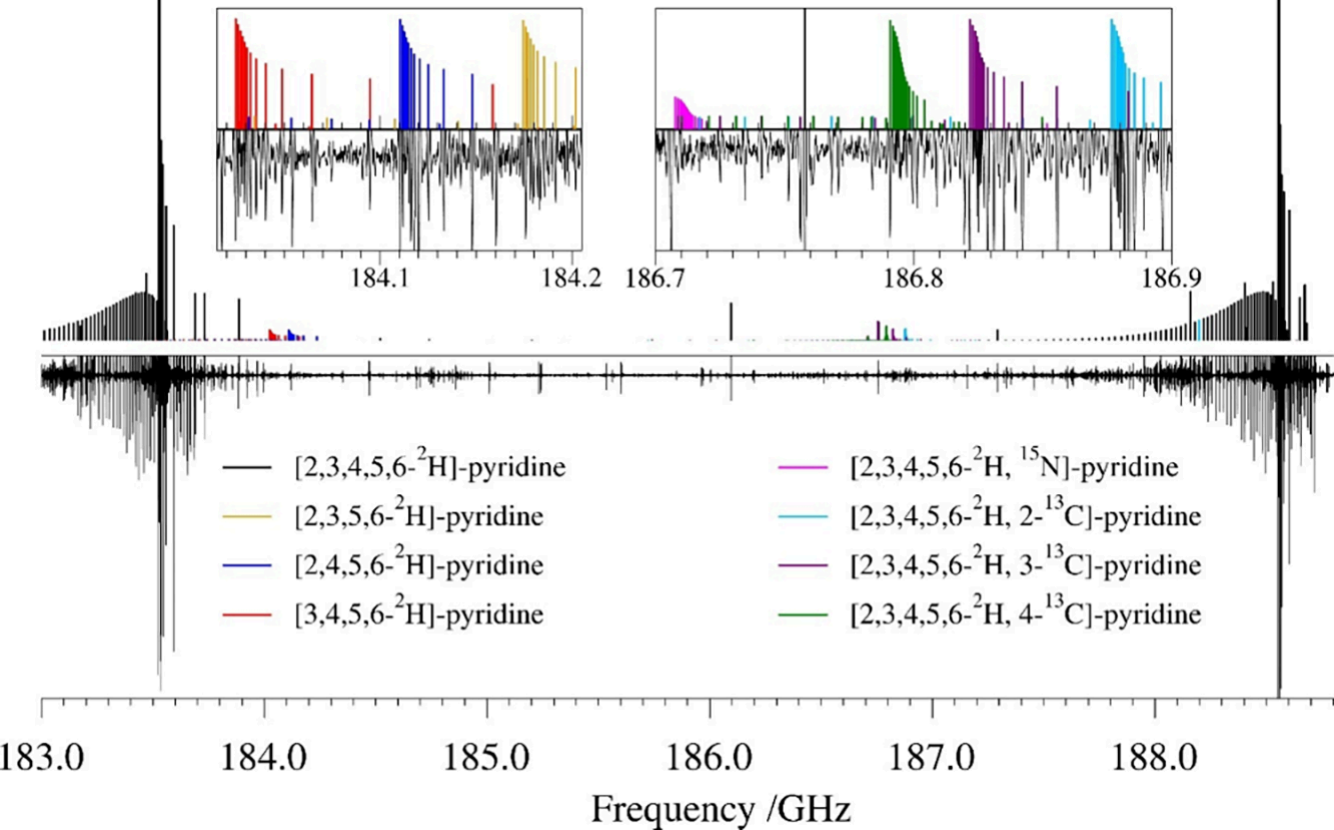
Experimental rotational spectrum (bottom) of a commercial sample of pyridine-*d*_5_ from 183.0 to 188.8 GHz and stick spectra (top) from experimental spectroscopic constants: [2,3,4,5,6-^2^H]-pyridine (black), [2,3,5,6-^2^H]-pyridine (gold), [2,4,5,6-^2^H]-pyridine (blue), [3,4,5,6-^2^H]-pyridine (red), [2,3,4,5,6-^2^H, ^15^N]-pyridine (pink), [2,3,4,5,6-^2^H, 2-^13^C]-pyridine (cyan), [2,3,4,5,6-^2^H, 3-^13^C]-pyridine (purple), and [2,3,4,5,6-^2^H, 4-^13^C]-pyridine (green). Unlabeled transitions are attributed to vibrationally excited states. The top-left inset shows an expansion of the 184.0–184.2 GHz region for improved visualization of pyridine-*d*_4_ isotopologues. The top-right inset shows an expansion of the 186.7–186.9 GHz region for improved visualization of the singly heavy-atom substituted isotopologues of [2,3,4,5,6-^2^H]-pyridine.

Unfortunately, even the well-predicted spectroscopic constants taken directly from the CCSD­(T)/cc-pCVTZ VPT2 calculation are not always sufficient to identify low-intensity species or species in regions of high spectral confusion, as was the case for some heavy-atom substituted pyridines and polydeuteriated isotopologues. In such cases, an improved prediction was generated using a preliminary, well-determined *r*_*e*_^SE^ structure in *xrefit*. Generally, the more isotopologues with accurately determined rotational constants incorporated into an *r*_*e*_^SE^ structure, the more accurate the prediction of rotational constants for other isotopologues becomes. Using the predicted equilibrium (*B*_*e*_) spectroscopic constants from *xrefit*, we applied the computed vibration–rotation interaction and electron-mass corrections to predict determinable constants. These constants were then used, together with the centrifugal distortion constants from the VPT2 calculation, to generate spectral predictions that very nearly landed on the bandheads of these hard-to-find species. A comparison of the VPT2 and *r*_*e*_^SE^-based predictions of the rotational constants is provided in the Supporting Information.

Table 2 presents the spectroscopic constants for one isotopologue that required this technique for identification: [2,3,4,5,6-^2^H, ^15^N]-pyridine. We showcase it here as an example for two points of interest. Although the computed rotational constants are again within 1% of the least-squares fitted values and the quartic centrifugal distortion terms are within 5% for values that could be determined, one should note some highly disconcerting observations about the least-squares fitted constants. In both reductions, the *A*_0_ and *B*_0_ values are exceptionally close to one another (and even *A*_0_ < *B*_0_), resulting from the extreme near-oblate asymmetric-top nature of this isotopologue. Although the value of *A*_*e*_ must necessarily be larger than *B*_*e*_, the inverted size is real for the observable constants. These issues are the result of both reductions breaking down for this nearly accidental oblate top (κ = 0.999 98, based on the *B*_*e*_ constants predicted by *xrefit*),^75^ similar to previously documented cases.^65–72^ This issue with the rotational constants affects how this isotopologue is incorporated into the *r*_*e*_^SE^ structure determination, which is the second point of interest (*vide infra*). The other heavy-atom substituted isotopologues of perdeuterio pyridine, or the tetradeuterio pyridine isotopologues, do not suffer the reduction breakdown. The spectroscopic constants and data distribution plots for these species are provided in the Supporting Information.

**Table 2:**
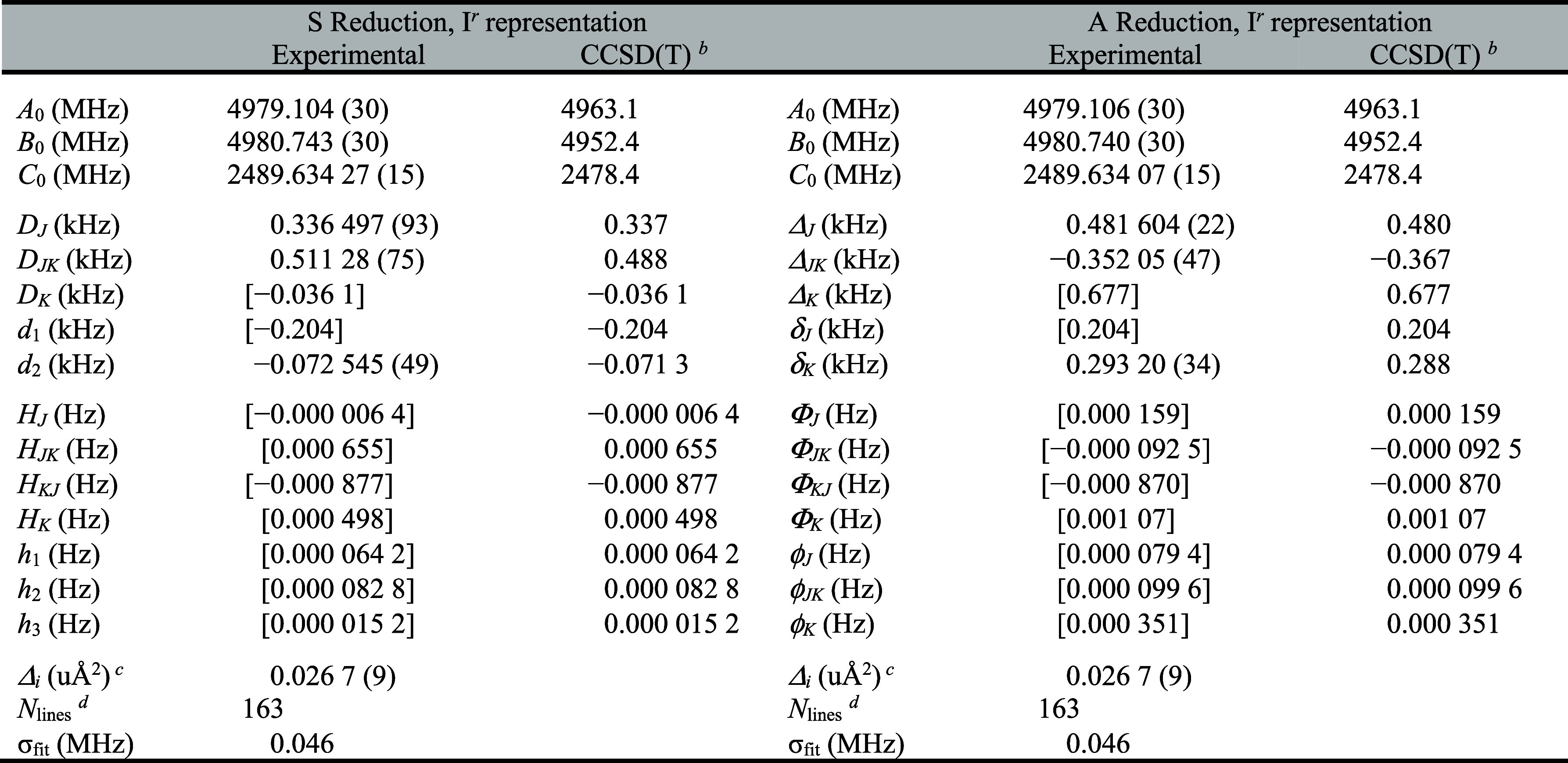
Experimental and Computed Spectroscopic Constants for [2,3,4,5,6-^2^H, ^15^N]-Pyridine^a^

a Values in square brackets held fixed at the computed value [CCSD­(T)/cc-pCVTZ] in the least-squares fit.

b Evaluated using the cc-pCVTZ basis set.

c Inertial defect, Δ_*i*_ = *I*_*c*_ – *I*_*a*_ – *I*_*b*_. Calculated using PLANM.

d Number of independent transitions.

### Semi-Experimental Equilibrium (*r*_*e*_^SE^) Structure of Pyridine

As mentioned, comparing the rotational constants of pyridine obtained using both the A and S reductions (I*^r^* representation) enabled a measure of quality control for the constants used in the *r*_*e*_^SE^ least-squares fit. The constants determined in each reduction were used to calculate the determinable rotational constants (*B*_0_^″^) using the equations provided in the Supporting Information.^76^ As long as the determinable constants from the A and S reductions agreed to within 0.008 MHz, the values were averaged for use in *xrefit*. To mitigate the effects of poorly determined rotational constants, determinable constants that did not agree to within 0.008 MHz were excluded from the structure determination by being assigned a weighting factor of zero. Specifically, *A*_0_^″^ and *B*_0_^″^ were zero-weighted for [3-^2^H, 2-^13^C]-, [3-^2^H, 3-^13^C]-, and [2,3,4,5,6-^2^H, 4-^13^C]-pyridine. *A*_0_^″^ was zero-weighted for [2,5-^2^H]-pyridine. Because the averaged determinable constants of [2,3,4,5,6-^2^H, ^15^N]-pyridine, like the determinable constants from each reduction, have *A*_0_^″^ < *B*_0_^″^, they could not be included in the structure determination, and only *C*_0_^″^ was used in the structure determination.^77^ (The *xrefit* module errs if the input rotational constants do not follow *A* > *B* > *C*.)

Within the *xrefit* module, the experimental determinable constants (*B*_0_^″^) for each isotopologue are corrected by computed vibration–rotation interaction constants and the electron-mass corrections to produce the equilibrium constants (*B*_*e*_); these values are subsequently used to determine the corresponding moments of inertia, inertial defects, and the resultant structure. The inertial defects (Table 3) exhibit the expected pattern, where the uncorrected inertial defect values (Δ_*i* 0_, from the *B*_0_^″^ constants) are small and their magnitude decreases with the inclusion of each correction (for vibration–rotation interaction and for electron-mass distribution), moving these values closer to the ideal value of zero for a planar molecule. Similar to previous cases,^2,3^ the magnitude of the fully corrected inertial defect is *ca*. 0.001 uÅ^2^. The consistency of inertial defects within each level of correction, throughout the list of isotopologues, gives confidence that the rotational constants are well-determined and accurate, and that the computational corrections are applied correctly. As in recent works,^1–3,54,60,71,78–80^ the fully corrected equilibrium inertial defects and those without electron-mass correction have the same, small standard deviations and even the same individual residuals for each isotopologue. The magnitude of the fully corrected inertial defects and their near-uniformity provides evidence of the high quality of both the corrections and the measurements, demonstrating that the equilibrium rotational constants used in computing the *r*_*e*_^SE^ structure are very well determined.

**Table 3:** Inertial Defects (Δ_*i*_) of Pyridine Isotopologues Used in the Current *r*_*e*_^SE^ Structure Determination

isotopologue	Δ_*i* 0_ (uÅ^2^)^a^	Δ_*i* e_ (uÅ^2^)^a^ ^,^ ^b^	Δ_*i* e_ (uÅ^2^)^a^ ^,^ ^c^
C_5_H_5_N	0.038 54	–0.013 27	–0.000 83
[^15^N]-C_5_H_5_N	0.039 13	–0.013 22	–0.000 78
[2-^13^C]-C_5_H_5_N	0.039 12	–0.013 32	–0.000 87
[3-^13^C]-C_5_H_5_N	0.039 16	–0.013 31	–0.000 87
[4-^13^C]-C_5_H_5_N	0.039 20	–0.013 24	–0.000 80
[2-^13^C, ^15^N]-C_5_H_5_N	0.039 70	–0.013 25	–0.000 81
[3-^13^C, ^15^N]-C_5_H_5_N	0.039 77	–0.013 24	–0.000 80
[4-^13^C, ^15^N]-C_5_H_5_N	0.039 77	–0.013 20	–0.000 76
[2-^2^H]-C_5_H_5_N	0.036 45	–0.013 48	–0.001 04
[3-^2^H]-C_5_H_5_N	0.036 45	–0.013 48	–0.001 04
[4-^2^H]-C_5_H_5_N	0.036 29	–0.013 24	–0.000 80
[3-^2^H, 2-^13^C]-C_5_H_5_N	0.037 05	–0.013 42	–0.000 98
[3-^2^H, 3-^13^C]-C_5_H_5_N	0.037 37	–0.013 11	–0.000 67
[3-^2^H, 4-^13^C]-C_5_H_5_N	0.037 29	–0.013 22	–0.000 77
[3-^2^H, 5-^13^C]-C_5_H_5_N	0.036 90	–0.013 67	–0.001 23
[3-^2^H, 6-^13^C]-C_5_H_5_N	0.036 98	–0.013 48	–0.001 04
[2,3-^2^H]-C_5_H_5_N	0.033 35	–0.013 69	–0.001 24
[2,5-^2^H]-C_5_H_5_N	0.032 98	–0.013 94	–0.001 49
[2,6-^2^H]-C_5_H_5_N	0.033 02	–0.013 45	–0.001 00
[3,4-^2^H]-C_5_H_5_N	0.034 22	–0.013 36	–0.000 92
[3,5-^2^H]-C_5_H_5_N	0.034 54	–0.013 29	–0.000 85
[2,4,6-^2^H]-C_5_H_5_N	0.030 75	–0.013 53	–0.001 09
[2,5,6-^2^H]-C_5_H_5_N	0.030 64	–0.013 54	–0.001 09
[3,4,5-^2^H]-C_5_H_5_N	0.032 20	–0.013 33	–0.000 89
[2,3,5,6-^2^H]-C_5_H_5_N	0.028 38	–0.013 46	–0.001 02
[2,4,5,6-^2^H]-C_5_H_5_N	0.028 48	–0.013 54	–0.001 09
[3,4,5,6-^2^H]-C_5_H_5_N	0.029 11	–0.013 52	–0.001 07
[2,3,4,5,6-^2^H]-C_5_H_5_N	0.026 13	–0.013 48	–0.001 03
[2,3,4,5,6-^2^H, ^15^N]-C_5_H_5_N	0.026 59^d^	–0.013 48^d^	–0.001 04^d^
[2,3,4,5,6-^2^H, 2-^13^C]-C_5_H_5_N	0.026 58	–0.013 53	–0.001 08
[2,3,4,5,6-^2^H, 3-^13^C]-C_5_H_5_N	0.026 54	–0.013 52	–0.001 07
[2,3,4,5,6-^2^H, 4-^13^C]-C_5_H_5_N	0.026 68	–0.013 47	–0.001 03
Average (*x̅*)	0.034 02	–0.013 42	–0.000 97
Std. Dev. (*s*)	0.004 70	0.000 17	0.000 17

a Inertial defect (Δ_*i*_ = *I*_*c*_ – *I*_*a*_ – *I*_*b*_).

b Inertial defect using *B*_*e*_ with vibration–rotation interaction corrections only.

c Inertial defect using *B*_*e*_ with vibration–rotation interaction and electron-mass corrections.

d Inertial defects were calculated manually using PLANM due to *xrefit* disallowing entry of values that do not conform to *A* > *B* > *C*.

The semiexperimental equilibrium structure of pyridine determined in the current study is presented in Table 4, alongside the previously determined semiexperimental equilibrium structures and the best theoretical estimate (BTE). As is evident from Table 4 and visually represented in Figure 4, the values of the parameters determined in all four semiexperimental structures display good agreement. In the current study, the *r*_*e*_^SE^ parameters are determined with greater precision, yet they agree, within the claimed 2σ uncertainties of the previous studies, with the values determined by Császár *et al*.,^46^ Piccardo *et al*.,^47,48^ and Penocchio *et al*.^49,50^ save two minor deviations: angle θ_C3–C4–H_ (Császár) and angle θ_C3–C2–H_ (Piccardo and Penocchio). Overall, the agreement between all three semiexperimental equilibrium structures supports their accurate determination in all four works and the validity of their uncertainty estimates.

**Table 4:** Semi-Experimental and Best Theoretical Estimate (BTE) Equilibrium Structures of Pyridine

parameter	*r*_*e*_^SE^ this work^a^	*r*_*e*_^SE^ Császár *et al*.^46^ ^,^ ^b^	*r*_*e*_^SE^ Piccardo *et al*.^47^ ^,^ ^b^	*r*_*e*_^SE^ Penocchio *et al*.^49^ ^,^ ^b^	*r*_*e*_ BTE this work
*R*_C2–H_ (Å)	1.0821 (1)	1.0816 (8)	1.0818 (4)	1.0821 (4)	1.0824
*R*_C3–H_ (Å)	1.0801 (1)	1.0795 (8)	1.0796 (4)	1.0799 (4)	1.0802
*R*_C4–H_ (Å)	1.0806 (2)	1.0803 (8)	1.0802 (4)	1.0806 (4)	1.0808
*R*_N–C2_ (Å)	*1.3355 (2)* ^c^	1.3362 (10)	1.3358 (10)	1.3356 (10)	1.3360^d^
*R*_C2–C3_ (Å)	1.3905 (2)	1.3902 (8)	1.3907 (4)	1.3907 (4)	1.3906
*R*_C3–C4_ (Å)	1.3883 (2)	1.3890 (8)	1.3888 (4)	1.3885 (4)	1.3886
*R*_C4–N_ (Å)	2.7980 (2)				2.7989
θ_C6–N–C2_ (°)	*116.973 (20)* ^c^	116.90 (8)	116.93 (4)	116.95 (4)	116.929^d^
θ_N–C2–C3_ (°)	*123.748 (15)* ^c^	123.80 (8)	123.79 (6)	123.77 (6)	123.773^d^
θ_C2–C3–C4_ (°)	118.544 (14)	118.54 (8)	118.53 (2)	118.54 (2)	118.544
θ_C3–C2–H_ (°)	120.316 (16)	120.30 (12)	120.25 (4)	120.24 (4)	120.301
θ_C4–C3–H_ (°)	121.332 (15)	121.34 (10)	121.37 (4)^d^	121.37 (4)^d^	121.317
θ_C3–C4–H_ (°)	120.778 (8)	120.71 (4)	120.78 (2)	120.78 (2)	120.782

a Using all available isotopologues (32 in total), with only *C*_0_^″^ used from [3-^2^H, 2-^13^C]-pyridine, [3-^2^H, 3-^13^C]-pyridine, [2,3,4,5,6-^2^H, 4-^13^C]-pyridine, and [2,3,4,5,6-^2^H, ^15^N]-pyridine and only *B*_0_^″^ and *C*_0_^″^ for [2,5-^2^H, ^15^N]-pyridine.

b Uncertainties reported as 1σ in the original articles are multiplied by two to achieve 2σ.

c Values were determined using an alternate *z*-matrix in the current work.

d Values were determined using mathematical relationships between available values.

**Figure 4:**
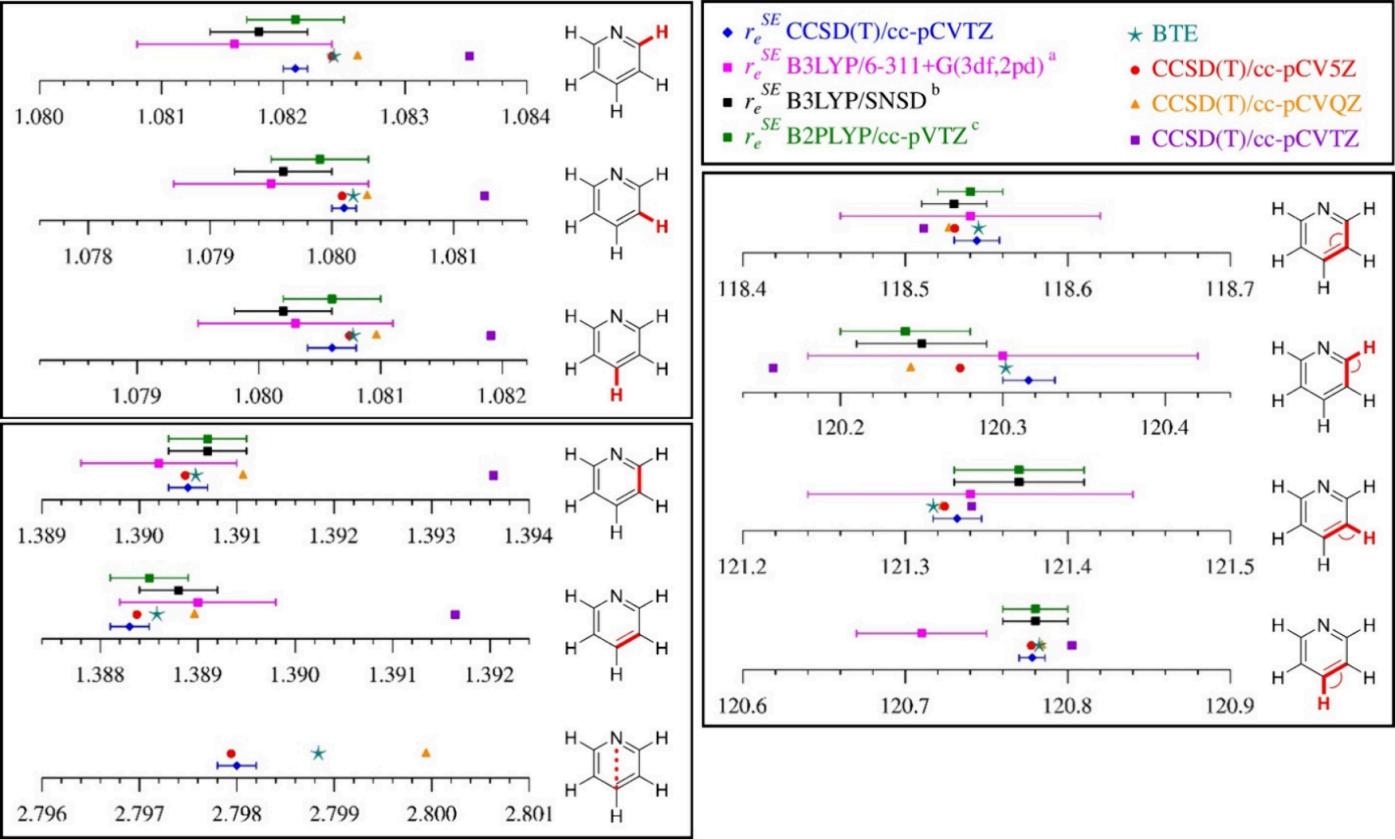
Graphical comparison of pyridine structural parameters with interatomic distances in Ångstrom (Å) and angles in degree (°). Uncertainties shown for current *r*_*e*_^SE^ CCSD­(T) are 2σ, while those for *r*_*e*_^SE^ structures (^a^) ref 46, (^b^) ref 47, and (^c^) ref 49 are displayed as 2× the reported 1σ uncertainties. The values of θ_C4–C3–H_ for (^b^) ref 47 and (^c^) ref 49 are calculated from their reported conjugate angles. The CCSD­(T)/cc-pCVQZ marker for θ_C4–C3–H_ is not observable because of overlap with the CCSD­(T)/cc-pCV5Z marker and is at a slightly lower value.

Comparing the values predicted as the best theoretical estimate with the current *r*_*e*_^SE^ structural parameters (Table 4), the level of agreement, viewed purely through the lens of whether the BTE values fall within the 2σ uncertainties, may not seem as good as that observed for similar structures. For both pyrimidine^3^ and pyridazine,^2^ the *r*_*e*_^SE^ structural parameters agree with the BTE values within the statistical experimental uncertainty (2σ) of each structural parameter. For pyridine, three of the six distances (*R*_C3–C4_, *R*_C2–H_, and *R*_C4–N_) fail to display this measure of agreement. This poorer apparent agreement between the *r*_*e*_^SE^ and BTE parameters for pyridine, relative to pyrimidine and pyridazine, may be a consequence of the smaller 2σ uncertainties associated with the (slightly) improved precision of the *r*_*e*_^SE^ structure for pyridine.^81^ A similar situation was observed in the case of benzene, where the BTE of the C–C bond distance did not agree with the *r*_*e*_^SE^ value to within the 2σ uncertainty of the highly precise *r*_*e*_^SE^ structure.^1^ Unlike other cases, the CCSD­(T)/cc-pCV5Z *r*_*e*_ structure parameters of pyridine, without BTE correction, actually fall within the 2σ uncertainties of the *r*_*e*_^SE^ values for all but one interatomic distance. The specific effects of the various corrections to the CCSD­(T)/cc-pCV5Z *r*_*e*_ structure parameters to obtain the BTE values are discussed in the Supporting Information. Based on the parameter values of the triple-zeta, quadruple-zeta, and quintuple-zeta *r*_*e*_ structures (Figure 4), it appears that the basis sets have converged for some parameters (θ_C2–C3–C4_, for example). It is not similarly clear that the computations have converged for the interatomic distances. It is possible that a higher-level calculation might improve the level of agreement; the necessary calculations, however, are very time and resource intensive.

### Quantifying the Isotopologue-Dependence of the Structure Determination

In order to further examine the accuracy and precision of the semiexperimental equilibrium structure, we examine the impact of each isotopologue on the final structure using *xrefiteration* (described in detail previously).^2^ Briefly, *xrefiteration* uses a core subset of isotopologues to determine an initial semiexperimental equilibrium structure and subsequently redetermines the structure by incorporating one isotopologue at a time until all isotopologues are incorporated into the structural least-squares fit. The order in which isotopologues are added is determined by their effect on the overall uncertainty in the structural parameters. At each step, the isotopologue that provides the largest decrease in uncertainty is incorporated—a sequence that often evolves to the eventual incorporation of isotopologues that increase the uncertainty. The value and 2σ uncertainty of each parameter can then be examined as a function of the isotopologues in the structural least-squares fit (Figure 5). The *xrefiteration* program is very useful in analyzing the effects of isotopologue incorporation, as well as identifying problematic issues in the data set.

**Figure 5:**
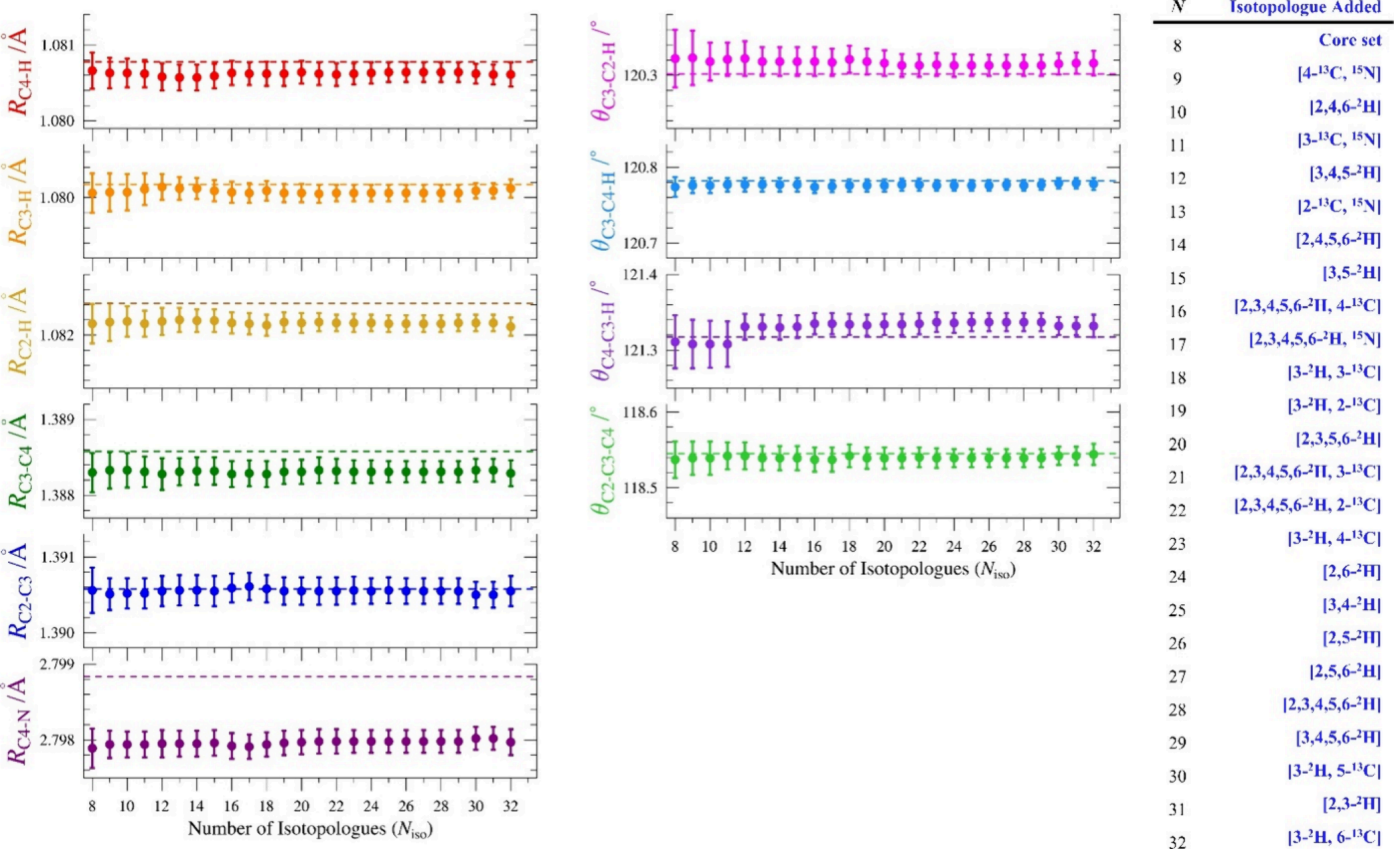
Plots of the structural parameters as a function of the number of isotopologues (*N*_iso_) and their 2σ uncertainties with consistent scales for each distance (0.0015 Å) and each angle (0.15°). The dashed line in each plot is the BTE value calculated for that parameter.^82^ The isotopologue ordering along the *x*-axis is provided in the table on the right.

In the case of this pyridine structure determination, the core set of eight isotopologues consists of the normal isotopologue plus the seven isotopologues that arise from single-atom substitution at each unique atom. There are a few general observations to be made. From the minimal core set of eight to the final 32 isotopologues, the values of the parameters change relatively little; all of the final values are within the uncertainties of their initial values. Incorporating additional isotopologues in the structure determination results, primarily, in decreasing the uncertainties. As isotopologues are added to the structure, the decrease in uncertainties is generally gradual, indicating that the isotopologues provide similar information for the structure. Some parameters, such as *R*_C4–N_ at the ninth isotopologue, *R*_C2–C3_ at the ninth isotopologue, or θ_C4–C3–H_ at the 12th isotopologue, have a relatively large improvement in the uncertainty, but these improvements do not occur for all parameters. Reasonably, this indicates that incorporation of new isotopologues can provide more structural information not already incorporated into the structure. The *R*_C4–N_ and *R*_C2–C3_ distances are a case in point. The values of both parameters change upon inclusion of the ninth isotopologue, with substantial decreases in both uncertainties (Figure 5). The ninth isotopologue, [4-^13^C, ^15^N]-pyridine, possesses an isotopic substitution pattern that would be expected to directly impact the determination of the cross-ring distance and, as a result, the *R*_C2–C3_ distance. Regarding the θ_C4–C3–H_ angle, the value of the parameter jumps upon inclusion of the 12th isotopologue with concomitant decrease in the parameter’s uncertainty (Figure 5). The 12th isotopologue, [3,4,5-^2^H]-pyridine, would be expected to provide information regarding the θ_C4–C3–H_ angle. While the value of this angle, supported by the 12th and subsequent isotopologues, falls within the uncertainties of the value determined by the core set and first three additional isotopologues, those first isotopologues provided substantial improvement to other parameters to be incorporated earlier in the order. The isotopologues beyond the 11th were necessary to improve both the accuracy and precision of the θ_C4–C3–H_ angle, specifically. For most parameters, however, the values vary only slightly as each isotopologue is added. By the addition of the 16th isotopologue, the changes in each parameter are well within the uncertainty of the previous step, indicating that the values are very likely converged. As is shown in the Supporting Information, due to the very large number of isotopologues in the final data set, no individual isotopologue is critical for this structure determination, *i.e.*, the large number of isotopologues provide redundant information to the structure determination. As with our previous works, no clear pattern has been identified regarding the order in which the isotopologues are added. Altogether, these observations indicate that the quality of data for each isotopologue is similar and that the overall quality is quite good.

The *xrefiteration* plot of parameter uncertainty values (δ*r*_*e*_^SE^) for the overall interatomic distances, angles, and combined uncertainties^2^ is presented in Figure 6. The plot of overall uncertainties, as well as those of the individual parameters, supports the idea that the overall structure is not converged using only eight isotopologues. Inspection of this plot, along with Figure 5 and the final structure confirms that no single isotopologue is critical to determining the structure of pyridine, *i.e.*, the removal of any single isotopologue does not have a significant impact on the final structure. Figure 6 shows that the impact of the first five isotopologues added is the most important to lowering the total uncertainty, then the impact of the additional isotopologues becomes less pronounced, but continues to decrease gradually through the addition of the 28th isotopologue. With the subsequent three isotopologue incorporations, a very slight increase is observed in the overall uncertainty. This behavior likely arises as the large data set of isotopologues causes the analysis to reach the limit of *r*_*e*_^SE^ structure determination imposed by the Born–Oppenheimer approximation, which assumes that all isotopologues share an equilibrium geometry. A breakdown of the Born–Oppenheimer approximation would result in a situation where incorporation of additional isotopologues works against those already used in the structure determination, causing an increase in the uncertainty of the parameters. It is likely that Figure 6 shows such a breakdown for the current structure of pyridine and incorporation of even more isotopologues will likely continue to very marginally increase the uncertainties of the parameters. The final isotopologue to be incorporated ([3-^2^H, 6-^13^C]-pyridine) causes a sharp increase in the uncertainties to approximately the same level as that of the 19th isotopologue, but does not cause a significant change in the value of any parameter (Figure 5). Only 98 transitions are measured for this isotopologue (one of the smallest data sets), and all of the transitions are degenerate. As a result, the values of *A*_0_ and *B*_0_ may still be somewhat convoluted with one another. Despite this concern, we have no definitive reason to suspect the data to be of poor quality. Small numbers of transitions are used to determine the constants of a few other isotopologues that do not elicit this behavior in the uncertainties, the determinable constants of the isotopologue in question are in good agreement, and the inertial defect (Table 3) does not clearly suggest an underlying problem. It is possible that an undetected issue remains with the spectroscopic constants of this isotopologue, or that it provided new information that changes the structure in a small but important manner, as evidenced by the small changes seen in the parameters at the end of Figure 5.

**Figure 6:**
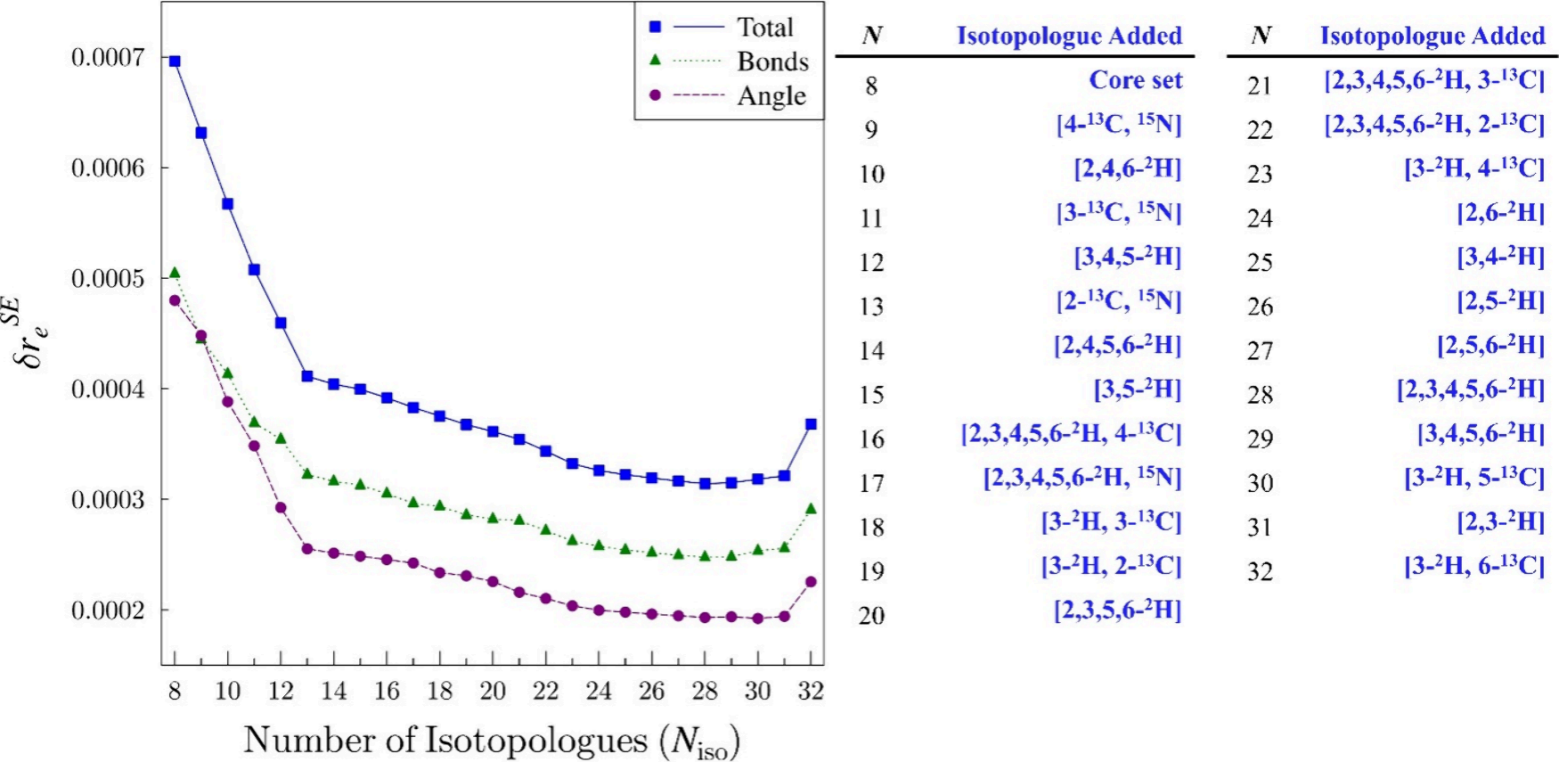
Plot of δ*r*_*e*_^SE^ values as a function of the number of isotopologues (*N*_iso_) incorporated in the structure determination. The total uncertainty (δ*r*_*e*_^SE^ Total, blue squares), the bond–distance uncertainty (δr*_e_* ^SE^ Bonds, green triangles), and the uncertainty in the angles (δ*r*_*e*_^SE^ Angle, purple circles) are presented. The isotopologue ordering along the *x*-axis is provided in the table on the right and is the same as that in Figure 5.

### Structural Comparisons: Gas-Phase *vs* Solid-State

The gas-phase (*r*_*e*_^SE^) structure of pyridine and a solid-state crystal structure are fundamentally different physical structures, yet comparison of gas-phase *vs* solid-state structure of pyridine is instructive (Figure 7). As determined by X-ray diffraction, the crystal structure of pyridine is unusually complex.^31–33^ There are four independent molecules in the unit cell, meaning that there are four independent sets of parameters to describe the molecular structure of pyridine in the solid state. The solid-state values presented in Figure 7 represent the numerical averages of the four individual parameters for each molecule in the unit cell.^31^ Qualitatively, the gas-phase and solid-state values display good agreement (Figure 7); there is no reason that these values must necessarily agree to within the experimental uncertainties of the measurements, and most do not. It is readily apparent that the gas-phase structural parameters are substantially more precise than those of the solid-state parameters. For bond distances and angles involving heavy atoms, gas-phase values are determined to five significant figures while solid-state values are determined to four. For bond distances involving a hydrogen atom, gas-phase values are determined to four digits beyond the decimal; solid-state values are determined only to two. Hydrogen atom positions are not well-determined by single-crystal X-ray diffraction,^83^ and this effect is clearly manifest in the lower accuracy and precision of bond distances and angles (involving hydrogen) in the solid-state structure of pyridine. That the solid-state parameters of pyridine need to be averaged over four discrete molecules in the unit cell further degrades the accuracy and precision of those values. The gas-phase semiexperimental equilibrium structure (*r*_*e*_^SE^) of pyridine in the current study represents the most accurate determination of molecular structure for pyridine yet reported. The equilibrium structure, by its very definition, is unencumbered by vibration, temperature, or effects of the solid state (intermolecular interaction, crystal packing forces, phonons, molecular vibrations).

**Figure 7:**
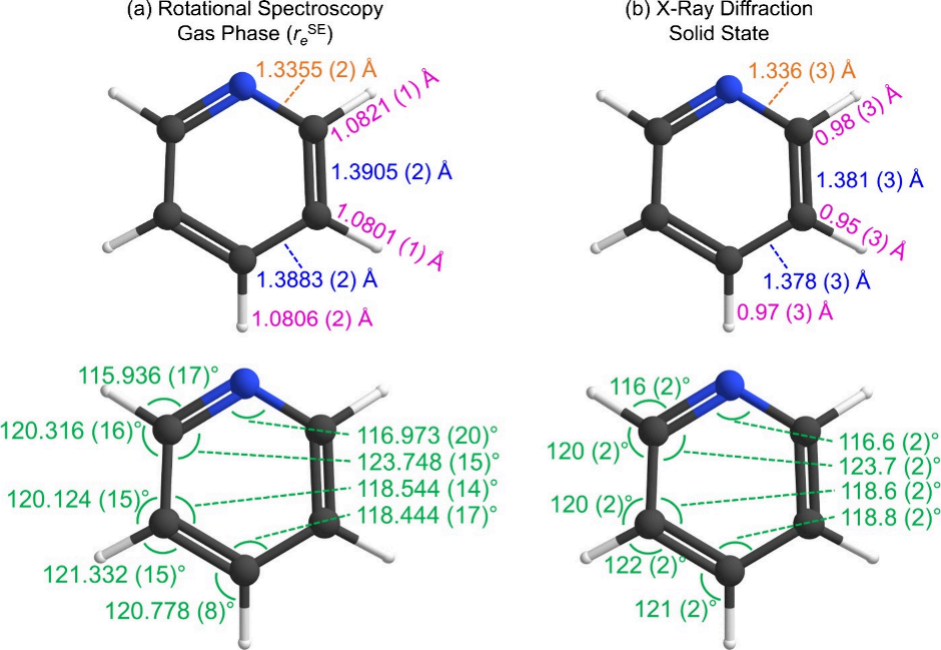
Structural parameters for pyridine: (a) from rotational spectroscopy/gas phase (*r*_*e*_^SE^) (this work) and (b) from X-ray crystallography/solid state.^31^ Distances are in Ångstrom (Å) and bond angles are in degree (°). The color of each value indicates the bond type: C–C (blue), C–N (gold), and C–H (pink). Angles are in green.

### Structural Comparisons: The Effect of Nitrogen Substitution in Aromatic Heterocycles

The current investigation provides critical structural data that enables a detailed analysis of the effects of nitrogen substitution in prototypical aromatic molecules. Only through comparisons among recently determined, highly precise structures for benzene,^1^ pyridine, pyridazine,^2^ and pyrimidine^3^ are the subtle structural effects of nitrogen substitution in the aromatic ring experimentally revealed. Semiexperimental equilibrium structure parameters of these species are depicted in Figure 8. The structural changes that occur upon nitrogen substitution within an aromatic ring are subtle, perhaps surprisingly so. In comparing the effects of a single nitrogen substitution—benzene *vs* pyridine—the primary structural changes occur in proximity to nitrogen. The C–N distance of pyridine is 0.0558 Å shorter than the corresponding C–C distance of benzene, while the vicinal C–H bond distance increases slightly (1.0821 Å *vs* 1.0809 Å) (Figure 8a). The C–N–C angle contracts slightly compared to the C–C–C angle (117° *vs* 120°), while the adjacent N–C–C angle compensates by expanding (124° *vs* 120°) (Figure 8b). The rest of the structural parameters for pyridine and benzene are remarkably similar, with the precise values of the differences depicted in Figure 8c. The structural perturbations introduced by heteroatom substitution are not manifest around the entire ring. While the remaining differences are small, they are real, as established through the high precision and accuracy of the *r*_*e*_^SE^ structure determinations.

**Figure 8:**
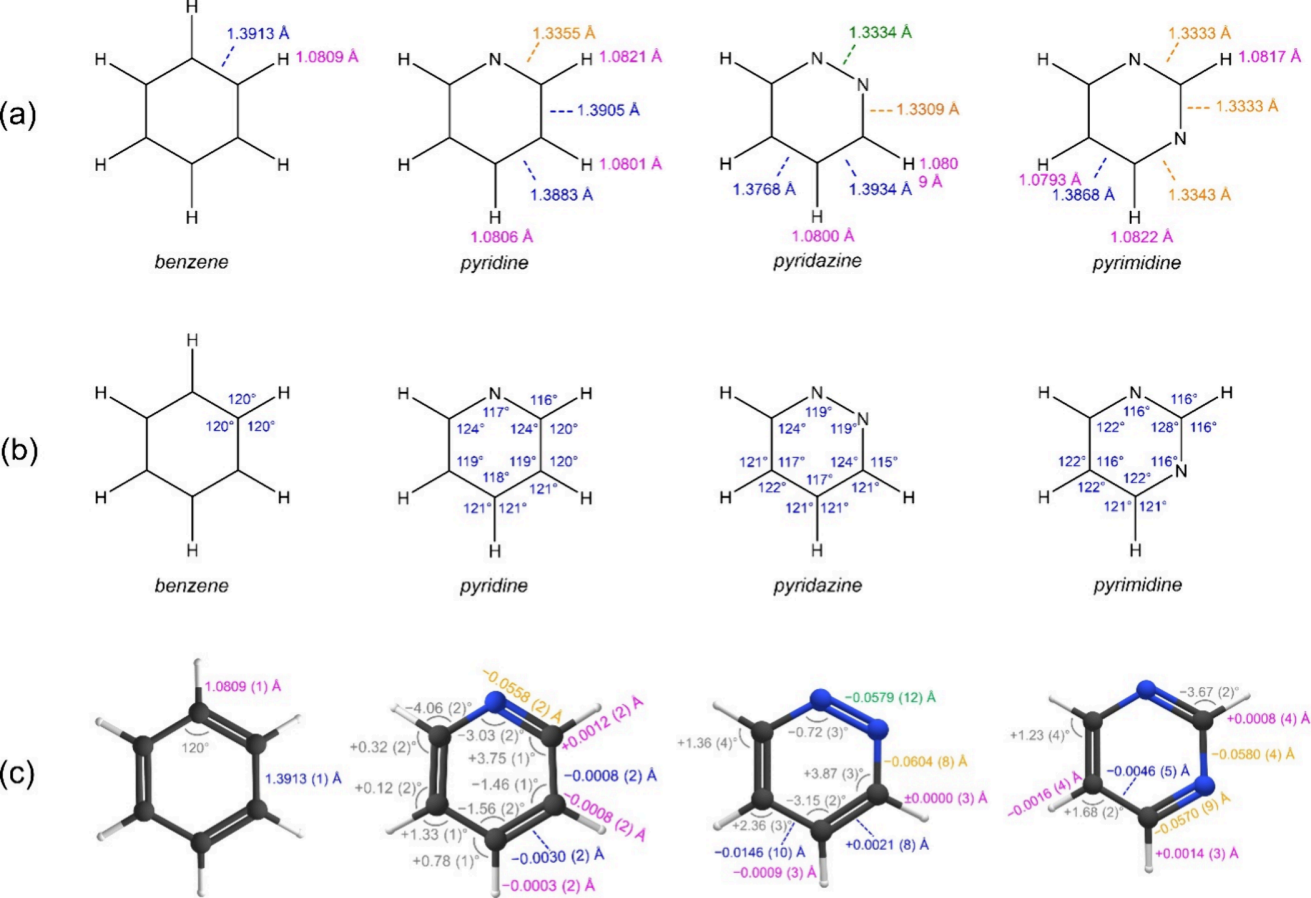
Gas-phase, semiexperimental equilibrium structures (*r*_*e*_^SE^) for benzene,^1^ pyridine (this work), pyridazine,^2^ and pyrimidine.^3^ (a) Bond distances in Ångstrom (Å). The color of each value indicates the bond type: C–C (blue), C–N (gold), N–N (green), and C–H (pink). (b) Bond angles in degree (°). (c) Changes in the parameter value, relative to benzene.

The structural changes that occur upon incorporation of a second nitrogen atom within an aromatic ring are remarkably similar to those observed upon incorporation of the first nitrogen atom (Figure 8). In the case of pyrimidine, the two unique C–N distances are within 0.001 Å of each other and within 0.002 Å of the corresponding C–N distance in pyridine. The C–C distances in benzene, pyridine, and pyrimidine are all within 0.0045 Å. While the C–H distances in pyridazine are quite similar to those of benzene (within 0.001 Å), these distances in pyridine and pyrimidine are elongated when adjacent to a nitrogen atom (by ∼0.001 Å) and slightly contracted when not adjacent to a nitrogen atom. Overall, it appears that the atomic compositions and bond type in molecules of similar structure determine the bond lengths to the hundredths of an Ångstrom, but require consideration of the broader structure beyond that precision. The C–N–C angle of pyrimidine contracts slightly, compared to benzene (116° *vs* 120°), while the adjacent N–C–C angle compensates by expanding (122° *vs* 120°) (Figure 8b). The N–C–N angle, which may be considered ‘doubly vicinal’ to the positions of nitrogen substitution, expands in a manner that is qualitatively double the effect of single substitution (128° pyrimidine, 124° pyridine, 120° benzene). For pyridazine, interpretation of structural data is complicated by the fact that the heteroatoms are adjacent. Interestingly, the N–N (1.3334 Å) and C–N (1.3309 Å) distances are very similar. The C–C distances of pyridazine (1.3934 Å, 1.3768 Å; difference = 0.0166 Å) are also more similar than one might nominally infer from the conventional rationalization in terms of the greater contribution of resonance structure B (Figure 9). Although the N–N–C angle in pyridazine (119°) is not significantly contracted, relative to benzene, the adjacent N–C–C angle is expanded (124°), as observed in each of the heterocycles in this comparison.

**Figure 9:**
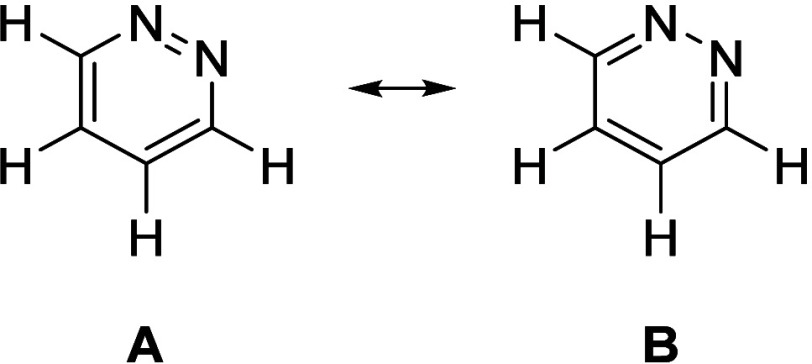
Canonical resonance structures for pyridazine.

## Conclusions

Structural changes associated with nitrogen-atom substitution in an aromatic ring are probed *via* high-resolution rotational spectroscopy. The current study of pyridine, along with recent works on benzene,^1^ pyridazine,^2^ and pyrimidine,^3^ affords substantial improvement in the accuracy of bond distances and angles. The importance of these improvements is amplified when considered in the context of the relative precision and accuracy that has now been realized for these small-parameter changes in the nitrogen heterocycles. The changes depicted in Figure 8 are likely the most precise measurements of the differences yet obtained for aromatic organic molecules in this size range, and the situation is particularly favorable in the present case of pyridine, where the combined 2σ uncertainties in benzene and pyridine are 0.0002 Å or less in all of the bond distances.

The primary structural changes that occur upon nitrogen substitution within an aromatic ring occur in proximity to nitrogen. The C–N distance is shorter (*ca*. 0.05 Å) than the corresponding C–C distance of benzene. The C–N–C angle contracts slightly, compared to the C–C–C angle (117° *vs* 120°), while the adjacent N–C–C angle compensates by expanding (124° *vs* 120°). The rest of the structural parameters for benzene and the heterocycles pyridine, pyridazine, and pyrimidine, are remarkably similar. These comparisons are possible only because methods for structure determination have evolved to afford sufficient precision to quantify these fundamental, yet subtle, structural effects. While single-crystal X-ray diffraction techniques are also powerful methods for structure determination, the structures are subject to crystal packing forces, which are more than sufficient to perturb a molecular structure at the level of precision that is considered here.

In the current study, the uncertainties associated with structural parameters for pyridine are decreased nearly to a fundamental limit of current *r*_*e*_^SE^ structure determination. Measurement precision is now so great that it challenges the basic assumption that isotopic substitution does not affect the equilibrium geometry of hydrogen-containing molecules.^1,54^

## Supplementary Material





## Notes

Computational output files, least-squares fitting output files, all A-reduction and S-reduction spectroscopic constants, equations used to calculate the BTE, data distribution plots, additional plots and figures mentioned in the text, the *r*_*e*_^SE^-predicted *B*_0_ values, chart of all possible deuterium isotopologues of pyridine, experimental details for isotopologue synthesis and characterization (PDF)
Least-squares fitting files, computational output files, *xrefit* files, *xrefiteration* files, output files used to compute the BTE (ZIP)

## ⊥

J.F.S.—deceased March 21, 2025.
