# Supplementary material for: Precise Semi-Experimental Equilibrium (r e SE) Structure of Pyridine from 32 Isotopologues: Accurate Assessment of the Effect of Nitrogen-Atom Substitution in Aromatic Rings
Source: J Phys Chem A. 2025 Nov 7;129(46):10737–50. doi: 10.1021/acs.jpca.5c07184 (PMC12641475; doi:10.1021/acs.jpca.5c07184)
Supplement: Supplementary file 1 [file jp5c07184_si_001.pdf]

Supporting Information

Precise Semi-Experimental Equilibrium (r_e^{SE}) Structure of Pyridine from 32 Isotopologues: Accurate Assessment of the Effect of Nitrogen-Atom Substitution in Aromatic Rings

Maria A. Zdanovskaia,[1] Brian J. Esselman,[1]

Samuel M. Kougias,[1] Madeleine G. Atwood,[1] Gregory H. Jones,[2]

John F. Stanton,[2,†] R. Claude Woods,[1,*] Robert J. McMahon[1,*]

[1] Department of Chemistry, University of Wisconsin–Madison, Madison, Wisconsin 53706, United States

[2] Quantum Theory Project, Departments of Physics and Chemistry, University of Florida, Gainesville, Florida 32611, United States

† Deceased March 21, 2025

* corresponding authors
E-mail address: rcwoods@wisc.edu (R.C. Woods)
E-mail address: robert.mcmahon@wisc.edu (R.J. McMahon)

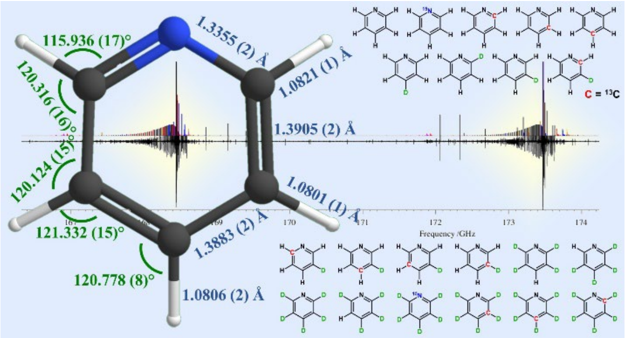

	Table of Contents	Page
**Eq. S1 – S6**.	Equations for calculating determinable constants	S7
**Eq. S7 – S13.**	Equations for Calculating the Best Theoretical Estimate (BTE)	S7 – S8
**Table S0.**	Corrections (ΔR) to CCSD(T)/cc-pCV5Z used to obtain the Best Theoretical Estimate [a]	S8
	**Overview of tables of spectroscopic constants and figures of data distribution plots**	S9
**Table S1.**	Experimental and computed spectroscopic constants for the normal isotopologue of pyridine.	S10
**Figure S1.**	Data distribution plot for the least-squares fit of millimeter-wave spectroscopic data for the normal isotopologue of pyridine	S11
**Table S2.**	Experimental and computed spectroscopic constants for [^{15N}]-pyridine	S12
**Figure S2.**	Data distribution plot for the least-squares fit of millimeter-wave spectroscopic data for [^{15}N]-pyridine	S13
**Table S3**.	Experimental and computed spectroscopic constants for [2-^{13}C]-pyridine	S14
**Figure S3.**	Data distribution plot for the least-squares fit of millimeter-wave spectroscopic data for [2-^{13}C]-pyridine	S15
**Table S4**.	Experimental and computed spectroscopic constants for [3-^{13}C]-pyridine	S16
**Figure S4.**	Data distribution plot for the least-squares fit of millimeter-wave spectroscopic data for [3-^{13}C]-pyridine	S17
**Table S5**.	Experimental and computed spectroscopic constants for [4-^{13}C]-pyridine	S18
**Figure S5.**	Data distribution plot for the least-squares fit of millimeter-wave spectroscopic data for [4-^{13}C]-pyridine	S19
**Table S6**.	Experimental and computed spectroscopic constants for [2-^{13}C, ^{15}N]-pyridine	S20
**Figure S6.**	Data distribution plot for the least-squares fit of millimeter-wave spectroscopic data for [2-^{13}C, ^{15}N]-pyridine	S21
**Table S7**.	Experimental and computed spectroscopic constants for [3-^{13}C, ^{15}N]-pyridine	S22
**Figure S7.**	Data distribution plot for the least-squares fit of millimeter-wave spectroscopic data for [3-^{13}C, ^{15}N]-pyridine	S23
**Table S8**.	Experimental and computed spectroscopic constants for [4-^{13}C, ^{15}N]-pyridine	S24
**Figure S8.**	Data distribution plot for the least-squares fit of millimeter-wave spectroscopic data for [4-^{13}C, ^{15}N]-pyridine	S25

Table of Contents		Page
**Table S9**.	Experimental and computed spectroscopic constants for [2-^{2}H]-pyridine	S26
**Figure S9.**	Data distribution plot for the least-squares fit of millimeter-wave spectroscopic data for [2-^{2}H]-pyridine	S27
**Table S10**.	Experimental and computed spectroscopic constants for [3-^{2}H]-pyridine	S28
**Figure S10.**	Data distribution plot for the least-squares fit of millimeter-wave spectroscopic data for [3-^{2}H]-pyridine	S29
**Table S11**.	Experimental and computed spectroscopic constants for [4-^{2}H]-pyridine	S30
**Figure S11.**	Data distribution plot for the least-squares fit of millimeter-wave spectroscopic data for [4-^{2}H]-pyridine	S31
**Table S12**.	Experimental and computed spectroscopic constants for [3-^{2}H, 2-^{13}C]-pyridine	S32
**Figure S12.**	Data distribution plot for the least-squares fit of millimeter-wave spectroscopic data for [3-^{2}H, 2-^{13}C]-pyridine	S33
**Table S13**.	Experimental and computed spectroscopic constants for [3-^{2}H, 3-^{13}C]-pyridine	S34
**Figure S13.**	Data distribution plot for the least-squares fit of millimeter-wave spectroscopic data for [3-^{2}H, 3-^{13}C]-pyridine	S35
**Table S14**.	Experimental and computed spectroscopic constants for [3-^{2}H, 4-^{13}C]-pyridine	S36
**Figure S14.**	Data distribution plot for the least-squares fit of millimeter-wave spectroscopic data for [3-^{2}H, 4-^{13}C]-pyridine	S37
**Table S15**.	Experimental and computed spectroscopic constants for [3-^{2}H, 5-^{13}C]-pyridine	S38
**Figure S15.**	Data distribution plot for the least-squares fit of millimeter-wave spectroscopic data for [3-^{2}H, 5-^{13}C]-pyridine	S39
**Table S16**.	Experimental and computed spectroscopic constants for [3-^{2}H, 6-^{13}C]-pyridine	S40
**Figure S16.**	Data distribution plot for the least-squares fit of millimeter-wave spectroscopic data for [3-^{2}H, 6-^{13}C]-pyridine	S41
**Table S17**.	Experimental and Computed Spectroscopic Constants for [2,3,4,5,6-^{2}H]-Pyridine	S42
**Figure S17.**	Data distribution plot for the least-squares fit of millimeter-wave spectroscopic data for [2,3,4,5,6-^{2}H]-pyridine	S43
**Table S18**.	Experimental and Computed Spectroscopic Constants for [2,3,4,5,6-^{2}H, ^{15}N]-Pyridine	S44
**Figure S18.**	Data distribution plot for the least-squares fit of millimeter-wave spectroscopic data for [2,3,4,5,6-^{2}H , ^{15}N]-pyridine	S45

Table of Contents		Page
**Table S19**.	Experimental and computed spectroscopic constants for [2,3,4,5,6-^{2}H , 2-^{13}C]-pyridine	S46
**Figure S19.**	Data distribution plot for the least-squares fit of millimeter-wave spectroscopic data for [2,3,4,5,6-^{2}H , 2-^{13}C]-pyridine	S47
**Table S20**.	Experimental and computed spectroscopic constants for [2,3,4,5,6-^{2}H , 3-^{13}C]-pyridine	S48
**Figure S20.**	Data distribution plot for the least-squares fit of millimeter-wave spectroscopic data for [2,3,4,5,6-^{2}H , 3-^{13}C]-pyridine	S49
**Table S21**.	Experimental and computed spectroscopic constants for [2,3,4,5,6-^{2}H , 4-^{13}C]-pyridine	S50
**Figure S21.**	Data distribution plot for the least-squares fit of millimeter-wave spectroscopic data for [2,3,4,5,6-^{2}H , 4-^{13}C]-pyridine	S51
**Table S22**.	Experimental and computed spectroscopic constants for [3,4,5,6-^{2}H]-pyridine	S52
**Figure S22.**	Data distribution plot for the least-squares fit of millimeter-wave spectroscopic data for [3,4,5,6-^{2}H]-pyridine	S53
**Table S23**.	Experimental and computed spectroscopic constants for [2,4,5,6-^{2}H]-pyridine	S54
**Figure S23.**	Data distribution plot for the least-squares fit of millimeter-wave spectroscopic data for [2,4,5,6-^{2}H]-pyridine	S55
**Table S24**.	Experimental and computed spectroscopic constants for [2,3,5,6-^{2}H]-pyridine	S56
**Figure S24.**	Data distribution plot for the least-squares fit of millimeter-wave spectroscopic data for [2,3,5,6-^{2}H]-pyridine	S57
**Table S25**.	Experimental and computed spectroscopic constants for [2,5,6-^{2}H]-pyridine	S58
**Figure S25.**	Data distribution plot for the least-squares fit of millimeter-wave spectroscopic data for [2,5,6-^{2}H]-pyridine	S59
**Table S26**.	Experimental and computed spectroscopic constants for [3,4,5-^{2}H]-pyridine	S60
**Figure S26.**	Data distribution plot for the least-squares fit of millimeter-wave spectroscopic data for [3,4,5-^{2}H]-pyridine	S61
**Table S27**.	Experimental and computed spectroscopic constants for [2,4,6-^{2}H]-pyridine	S62
**Figure S27.**	Data distribution plot for the least-squares fit of millimeter-wave spectroscopic data for [2,4,6-^{2}H]-pyridine	S63
**Table S28**.	Experimental and computed spectroscopic constants for [2,3-^{2}H]-pyridine	S64
**Figure S28.**	Data distribution plot for the least-squares fit of millimeter-wave spectroscopic data for [2,3-^{2}H]-pyridine	S65

Table of Contents		Page
**Table S29**.	Experimental and computed spectroscopic constants for [2,5-^{2}H]-pyridine	S66
**Figure S29.**	Data distribution plot for the least-squares fit of millimeter-wave spectroscopic data for [2,5-^{2}H]-pyridine	S67
**Table S30**.	Experimental and computed spectroscopic constants for [2,6-^{2}H]-pyridine	S68
**Figure S30.**	Data distribution plot for the least-squares fit of millimeter-wave spectroscopic data for [2,6-^{2}H]-pyridine	S69
**Table S31**.	Experimental and computed spectroscopic constants for [3,4-^{2}H]-pyridine	S70
**Figure S31.**	Data distribution plot for the least-squares fit of millimeter-wave spectroscopic data for [3,4-^{2}H]-pyridine	S71
**Table S32**.	Experimental and computed spectroscopic constants for [3,5-^{2}H]-pyridine	S72
**Figure S32.**	Data distribution plot for the least-squares fit of millimeter-wave spectroscopic data for [3,5-^{2}H]-pyridine	S73
**Table S33**.	Differences between experimental determinable constants and those predicted by CCSD(T) and by *xrefit*	S74
**Table S34**.	Semi-experimental and best theoretical estimate equilibrium structures of pyridine using variable numbers of isotopologues for current r_e^{SE}	S76
Materials / synthesis / chart of deuterium isotopologues		S77 - S79
Experimental procedures for preparation of isotopologues		S80 – S82
Experimental mass spectra, ^{1}H NMR spectra, ^{2}H NMR spectra		S82 - S89
**References**		S90
CCSD(T) / cc-pCVTZ geometry optimization (*pyridine_opt_cc-pCVTZ.out*)		Separate file
CCSD(T) / cc-pCVTZ VPT2 anharmonic frequency calculation (*anharm.out*)		Separate file
CCSD(T) / cc-pCVTZ Isomass files containing computed distortion constants for pyridine isotopologues		Separate files
CCSD(T) / cc-pCVTZ magnetic calculations (*pyridine_magnetic_Iso##.out*)		Separate files
Xrefit r_e^{SE} structure output (*pyridine_reSE.out*)		Separate file
Xrefit output without computational corrections (provides $\Delta_{i\,0}$) (*pyridine_r0.out*)		Separate file

Table of Contents	Page
Xrefit output without electron-mass distribution correction (provides $\Delta_{i\ \mathrm{e}}$ using vibration-rotation interaction correction only) (*pyridine_reSE_sans_e.out*)	Separate file
Xrefit r_e^{SE} structure output with alternate *z*-matrix for molecular radius (*pyridine_reSE_altZMAT.out)*	Separate file
Xrefiteration output (*INPUT-results.csv*)	Separate file
Additional CFOUR optimization output files used to calculate BTE	Separate files

Equations for Calculating the Determinable Constants

$$A_0'' = A_0^{(A)} + 2\Delta_{\mathrm{J}} \quad \text{(S1)}$$
$$B_0'' = B_0^{(A)} + 2\Delta_{\mathrm{J}} + \Delta_{\mathrm{JK}} - 2\delta_J - 2\delta_K \quad \text{(S2)}$$
$$C_0'' = C_0^{(A)} + 2\Delta_{\mathrm{J}} + \Delta_{\mathrm{JK}} + 2\delta_J + 2\delta_K \quad \text{(S3)}$$

$$A_0'' = A_0^{(S)} + 2D_{\mathrm{J}} + 6d_2 \quad \text{(S4)}$$
$$B_0'' = B_0^{(S)} + 2D_J + D_{JK} + 2d_1 + 4d_2 \quad \text{(S5)}$$
$$C_0'' = C_0^{(S)} + 2D_J + D_{JK} - 2d_1 + 4d_2 \quad \text{(S6)}$$

Equations for Calculating the Best Theoretical Estimate (BTE)

1. Residual basis set effects beyond cc-pCV5Z: To estimate the correction needed to approach the infinite basis set limit, equilibrium structural parameters obtained with the cc-pCVXZ (X = T, Q, and 5) basis sets were extrapolated using the empirical exponential expression in Eq. (S7).[1]

$$R(x) = R(\infty) + Ae^{-Bx} \quad \text{(S7)}$$

R(x) are the values of the parameters obtained using the various basis sets (x = 3, 4, and 5), and R(∞) is the desired basis set limit estimate. Using three basis sets (x = 3, 4, and 5), the system of equations using Eq. (S7) can be solved, yielding Eq. (S8).

$$R(\infty) = -\frac{R(4)^2 - R(3)R(5)}{R(3) + R(5) - 2R(4)} \quad \text{(S8)}$$

The correction to the structure due to a finite basis set is estimated by Eq. (S9).

$$\Delta R(\text{basis}) = R(\infty) - R(\text{CCSD(T)/cc-pCV5Z}) \quad \text{(S9)}$$

2. Residual electron correlation effects beyond the CCSD(T) treatment: Residual correlation effects are assessed by doing geometry optimizations at the CCSDT(Q) level[2] and then estimating the correlation correction in Eq. (S10).

$$\Delta R(\text{cor}) = R(\text{CCSDT(Q)}) - R(\text{CCSD(T)}) \quad \text{(S10)}$$

   As calculations at the CCSDT(Q) level of theory are quite expensive, these two calculations are obtained with the cc-pVDZ basis, in the frozen-core approximation.

3. Effects of scalar (mass-velocity and Darwin) relativistic effects: The relativistic corrections are obtained by subtraction of the equilibrium parameters obtained with a standard non-relativistic calculation from those obtained with the X2C-1e variant of coupled-cluster theory,[3-5] as shown in Eq. (S11).

$$\Delta \mathrm{R(rel)} = R(\mathrm{CCSD(T)/cc\text{-}pCVTZ})_{\mathrm{SFX2C\text{-}1e}} - R(\mathrm{CCSD(T)/cc\text{-}pCVTZ})_{NR} \quad \text{(S11)}$$

4. The diagonal Born-Oppenheimer correction (DBOC): The diagonal Born-Oppenheimer correction (DBOC)[6] is obtained from Eq. (S12).

$$\Delta R(\mathrm{DBOC}) = R(\mathrm{SCF/cc\text{-}pVTZ})_{DBOC} - R(\mathrm{SCF/cc\text{-}pVTZ})_{NR} \quad \text{(S12)}$$

Here, the first value is obtained by minimizing the DBOC-corrected SCF energy with respect to nuclear positions, and the latter is again the traditional calculation.

The sum of the above-described corrections is used to obtain the best equilibrium structural parameters, given by Eq. (S13).

$$\Delta R(\mathrm{best}) = \Delta R(\mathrm{basis}) + \Delta R(\mathrm{cor}) + \Delta R(\mathrm{rel}) + \Delta R(\mathrm{DBOC}) \quad \text{(S13)}$$

The sum of the corrections, ΔR(best), is applied to the CCSD(T)/cc-pCV5Z structural parameters.

Table S0. Corrections (Δ*R*) to CCSD(T)/cc-pCV5Z used to obtain the Best Theoretical Estimate [a]

Parameter	ΔR(basis)	ΔR(rel)	ΔR(corr)	ΔR(DBOC)	ΔR(Best)
$R_{\mathrm{C2–H}}$ (Å) [b]	−0.000 06	−0.000 12	0.000 06	0.000 13	0.000 02
$R_{\mathrm{C3–H}}$ (Å)	−0.000 06	−0.000 11	0.000 13	0.000 13	0.000 09
$R_{\mathrm{C4–H}}$ (Å)	−0.000 07	−0.000 11	0.000 07	0.000 14	0.000 03
$R_{\mathrm{C2–C3}}$ (Å)	−0.000 18	−0.000 25	0.000 52	0.000 01	0.000 11
$R_{\mathrm{C3–C4}}$ (Å) [b]	−0.000 17	−0.000 24	0.000 60	0.000 02	0.000 20
$R_{\mathrm{C4–N}}$ (Å) [b]	−0.000 63	−0.000 15	0.001 65	0.000 02	0.000 90
$\theta_{\mathrm{C2–C3–C4}}$ (°)	0.001	0.001	0.012	0.000	0.015
$\theta_{\mathrm{C3–C2–H}}$ (°)	0.017	0.003	0.005	0.003	0.028
$\theta_{\mathrm{C4–C3–H}}$ (°)	0.000	0.000	−0.008	0.000	−0.007
$\theta_{\mathrm{C3–C4–H}}$ (°)	−0.003	0.001	0.007	0.000	0.005

[a] Exact values were used for all mathematical functions, but values in table are rounded to one decimal more than provided in structural determination for legibility, resulting in apparent discrepancies between sum of rounded corrections shown and rounded ΔR(Best).
[b] Rows highlighted in grey indicate parameters for which the value of the BTE falls outside 2σ of the r_e^{SE} value.

As is evident from the computed structural data plotted in Figure 4 of the manuscript, using basis sets with increasing functions, *i.e.*, cc-pCVTZ to cc-pCVQZ to cc-pCV5Z, results in shorter estimates of the interatomic distances. Extrapolation of each parameter to the infinite basis limit assuming exponential convergence results in a modification to the cc-pCV5Z interatomic distances that further shortens them (ΔR(basis), Table S0). Correction for relativistic effects also shortens the interatomic distances, whereas residual electron correlation and the diagonal Born-Oppenheimer corrections increase the distances. Moreover, for any parameters where the value of ΔR(corr) is small (less than the mean value of 0.000 51), the value of ΔR(DBOC) is large (greater than the mean value of 0.000 08), and *vice versa*. The same pattern is observed for pyrimidine, but not for pyridazine. The origin of overestimation cannot be isolated to a single correction, even when specifically examining the parameters that were not within agreement between r_e and r_e^{SE}.

Overview of tables of spectroscopic constants and figures of data distribution plots. For archival purposes, data for all isotoplogues are included in the Supporting Information, even if small portions of these data have been included in the manuscript. For all of the tables, it should be noted: Values in square brackets have been held constant at the computed value [CCSD(T)/cc-pCVTZ] in the least-squares fit.

Table S1. Experimental and computed spectroscopic constants for the normal isotopologue of pyridine

	S Reduction, I^r representation			A Reduction, I^r representation	
	Experimental [a]	CCSD(T) [b]		Experimental [a]	CCSD(T) [b]
A_0 (MHz)	6039.248 578 (36)	6020	A_0 (MHz)	6039.247 540 (36)	6020
B_0 (MHz)	5804.909 820 (32)	5770	B_0 (MHz)	5804.911 212 (32)	5770
C_0 (MHz)	2959.210 936 (26)	2945	C_0 (MHz)	2959.210 366 (26)	2945
D_J (kHz)	0.547 821 4 (80)	0.538	Δ_J (kHz)	0.758 197 2 (79)	0.743
D_{JK} (kHz)	0.656 954 (32)	0.641	Δ_{JK} (kHz)	−0.605 321 (23)	−0.588
D_K (kHz)	0.170 298 (30)	0.189	Δ_K (kHz)	1.222 209 (22)	1.213
d_1 (kHz)	−0.322 835 6 (37)	−0.317	δ_J (kHz)	0.322 835 4 (37)	0.317
d_2 (kHz)	−0.105 189 7 (16)	−0.102	δ_K (kHz)	0.490 069 8 (75)	0.484
H_J (Hz)	0.000 044 5 (12)	0.000 026 5	Φ_J (Hz)	0.000 356 4 (10)	0.000 338
H_{JK} (Hz)	0.001 227 5 (79)	0.001 30	Φ_{JK} (Hz)	−0.000 148 6 (47)	−0.000 060 4
H_{KJ} (Hz)	−0.002 295 (11)	−0.002 29	Φ_{KJ} (Hz)	−0.002 392 7 (70)	−0.002 42
H_K (Hz)	0.001 644 9 (62)	0.001 68	Φ_K (Hz)	0.002 810 2 (44)	0.002 86
h_1 (Hz)	0.000 148 87 (53)	0.000 142	ϕ_J (Hz)	0.000 175 33 (51)	0.000 168
h_2 (Hz)	0.000 156 18 (34)	0.000 156	ϕ_{JK} (Hz)	0.000 311 8 (17)	0.000 289
h_3 (Hz)	0.000 026 708 (75)	0.000 0267	ϕ_K (Hz)	0.000 807 4 (18)	0.000 817
N_{lines} [c]	6680		N_{lines} [c]	6680	
σ_{fit} (MHz)	0.040		σ_{fit} (MHz)	0.040	

[a] Includes transitions from previous work.[7] [b] Evaluated using the cc-pCVTZ basis set. [c] Number of independent transitions.

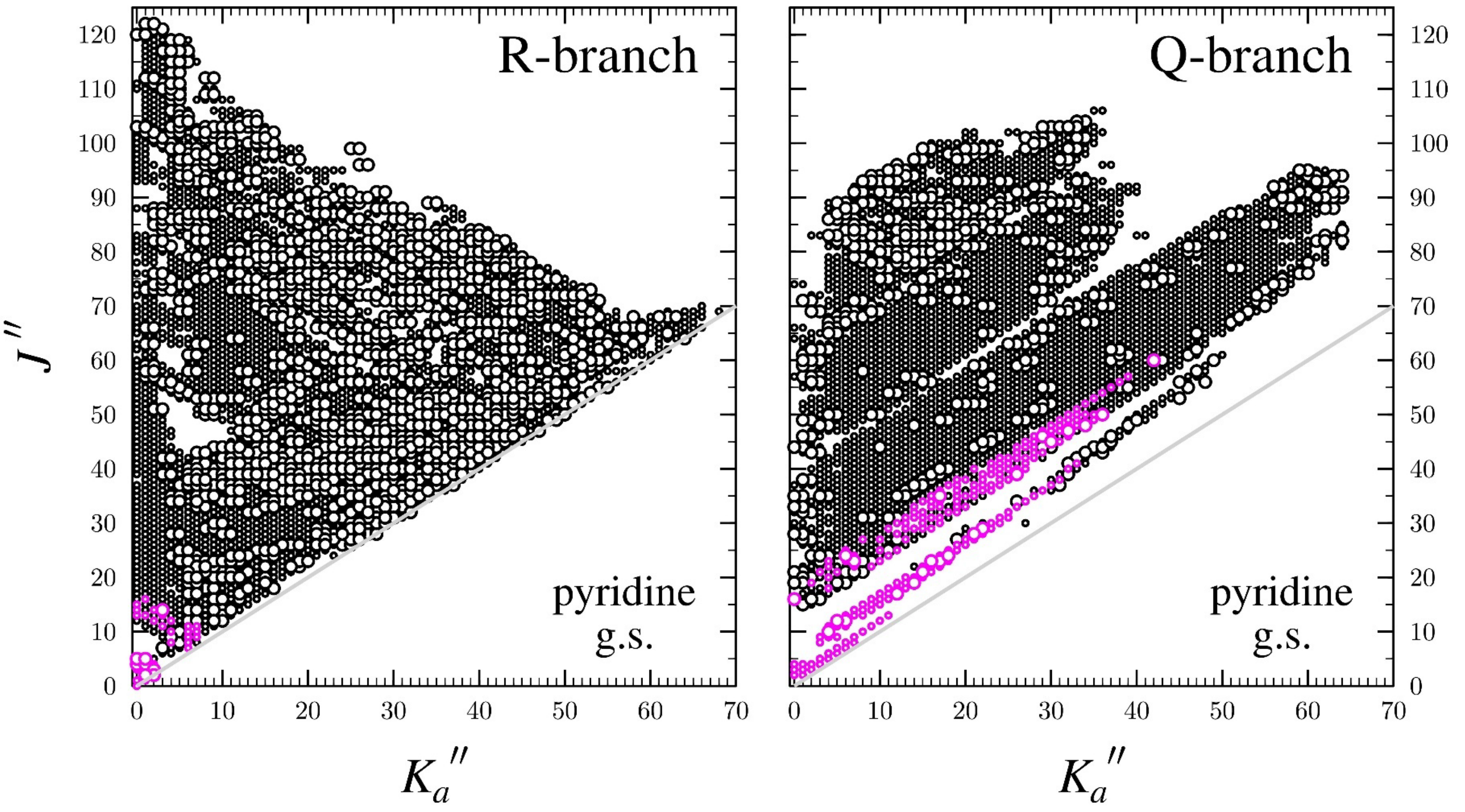

Figure S1. Data distribution plot for the least-squares fit of millimeter-wave spectroscopic data for the normal isotopologue of pyridine, ground vibrational state. Black circles are measurements from the current work, while magenta circles are from previous work.[7] The size of the outlined circle is proportional to the value of $|(f_{obs.} - f_{calc.})/\delta f|$, where δf is the frequency measurement uncertainty (50 kHz), and no quotient values are larger than three.

Table S2. Experimental and computed spectroscopic constants for [^{15}N]-pyridine

	S Reduction, I^r representation			A Reduction, I^r representation	
	Experimental [a]	CCSD(T) [b]		Experimental [a]	CCSD(T) [b]
A_0 (MHz)	6039.421 929 (41)	6020	A_0 (MHz)	6039.420 960 (41)	6020
B_0 (MHz)	5680.386 824 (38)	5646	B_0 (MHz)	5680.388 202 (38)	5646
C_0 (MHz)	2926.537 620 (41)	2913	C_0 (MHz)	2926.537 021 (41)	2913
D_J (kHz)	0.541 785 (17)	0.532	Δ_J (kHz)	0.737 167 (16)	0.722
D_{JK} (kHz)	0.575 858 (49)	0.562	Δ_{JK} (kHz)	−0.596 439 (35)	−0.580
D_K (kHz)	0.257 939 (59)	0.275	Δ_K (kHz)	1.234 862 (43)	1.226
d_1 (kHz)	−0.313 265 4 (53)	−0.307	δ_J (kHz)	0.313 266 8 (53)	0.307
d_2 (kHz)	−0.097 691 0 (28)	−0.095	δ_K (kHz)	0.492 668 (14)	0.486
H_J (Hz)	0.000 056 4 (26)	0.000 054 1	Φ_J (Hz)	0.000 335 1 (21)	0.000 331
H_{JK} (Hz)	0.001 068 (15)	0.001 06	Φ_{JK} (Hz)	−0.000 078 2 (98)	−0.000 085 5
H_{KJ} (Hz)	−0.002 088 (24)	−0.002	Φ_{KJ} (Hz)	−0.002 452 (18)	−0.002 35
H_K (Hz)	0.001 699 (19)	0.001 61	Φ_K (Hz)	0.002 933 (14)	0.002 82
h_1 (Hz)	0.000 139 70 (73)	0.000 140	ϕ_J (Hz)	0.000 165 92 (76)	0.000 165
h_2 (Hz)	0.000 139 32 (75)	0.000 139	ϕ_{JK} (Hz)	0.000 273 1 (41)	0.000 278
h_3 (Hz)	0.000 026 17 (16)	0.000 025 5	ϕ_K (Hz)	0.000 869 5 (41)	0.000 863
N_{lines} [c]	3770		N_{lines} [c]	3770	
σ_{fit} (MHz)	0.031		σ_{fit} (MHz)	0.031	

[a] Includes transitions from previous work.[8] [b] Evaluated using the cc-pCVTZ basis set. [c] Number of independent transitions.

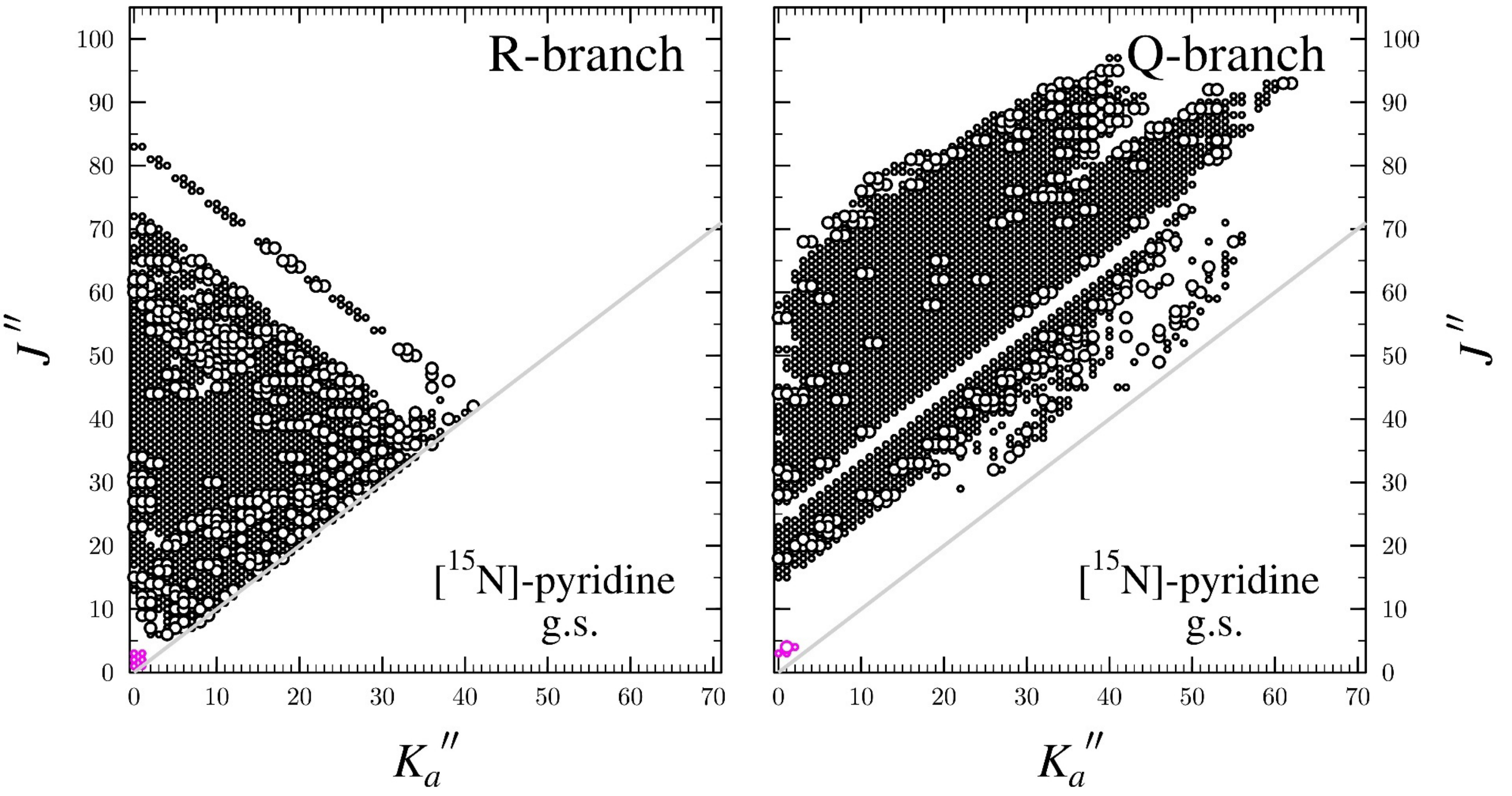

Figure S2. Data distribution plot for the least-squares fit of millimeter-wave spectroscopic data for [^{15}N]-pyridine, ground vibrational state. Black circles are measurements from the current work, while magenta circles are from previous work.[8] The size of the outlined circle is proportional to the value of $|(f_{obs.} - f_{calc.})/\delta f|$, where δf is the frequency measurement uncertainty (50 kHz), and no quotient values are larger than three.

Table S3. Experimental and computed spectroscopic constants for [2-^{13}C]-pyridine

	S Reduction, I^r representation			A Reduction, I^r representation	
	Experimental [a]	CCSD(T) [b]		Experimental [a]	CCSD(T) [b]
A_0 (MHz)	5963.118 13 (17)	5943	A_0 (MHz)	5963.117 12 (17)	5943
B_0 (MHz)	5758.945 29 (12)	5726	B_0 (MHz)	5758.946 63 (12)	5726
C_0 (MHz)	2928.960 765 (72)	2915	C_0 (MHz)	2928.960 175 (71)	2915
D_J (kHz)	0.502 188 (91)	0.497	Δ_J (kHz)	0.728 215 (53)	0.716
D_{JK} (kHz)	0.894 43 (64)	0.851	Δ_{JK} (kHz)	−0.462 26 (35)	−0.462
D_K (kHz)	−0.072 04 (66)	−0.0307	Δ_K (kHz)	1.059 00 (40)	1.064
d_1 (kHz)	−0.309 013 (23)	−0.304	δ_J (kHz)	0.308 976 (22)	0.304
d_2 (kHz)	−0.113 048 (26)	−0.109	δ_K (kHz)	0.517 32 (12)	0.507
H_J (Hz)	−0.000 047 1 (43)	−0.000 056 5	Φ_J (Hz)	0.000 3186 (37)	0.000 308
H_{JK} (Hz)	0.001 742 (35)	0.001 50	Φ_{JK} (Hz)	[−0.000 0154]	−0.000 015 4
H_{KJ} (Hz)	−0.002 280 (90)	−0.001 58	Φ_{KJ} (Hz)	−0.002 230 (63)	−0.002 00
H_K (Hz)	0.000 959 (71)	0.000 760	Φ_K (Hz)	0.002 448 (63)	0.002 33
h_1 (Hz)	[0.000 117]	0.000 117	ϕ_J (Hz)	[0.000 154]	0.000 154
h_2 (Hz)	[0.000 182]	0.000 182	ϕ_{JK} (Hz)	[0.000 28]	0.000 28
h_3 (Hz)	[0.000 0364]	0.000 036 4	ϕ_K (Hz)	[0.000 917]	0.000 917
N_{lines} [c]	1589		N_{lines} [c]	1589	
σ_{fit} (MHz)	0.039		σ_{fit} (MHz)	0.039	

[a] Includes transitions from previous work.[8] [b] Evaluated using the cc-pCVTZ basis set. [c] Number of independent transitions.

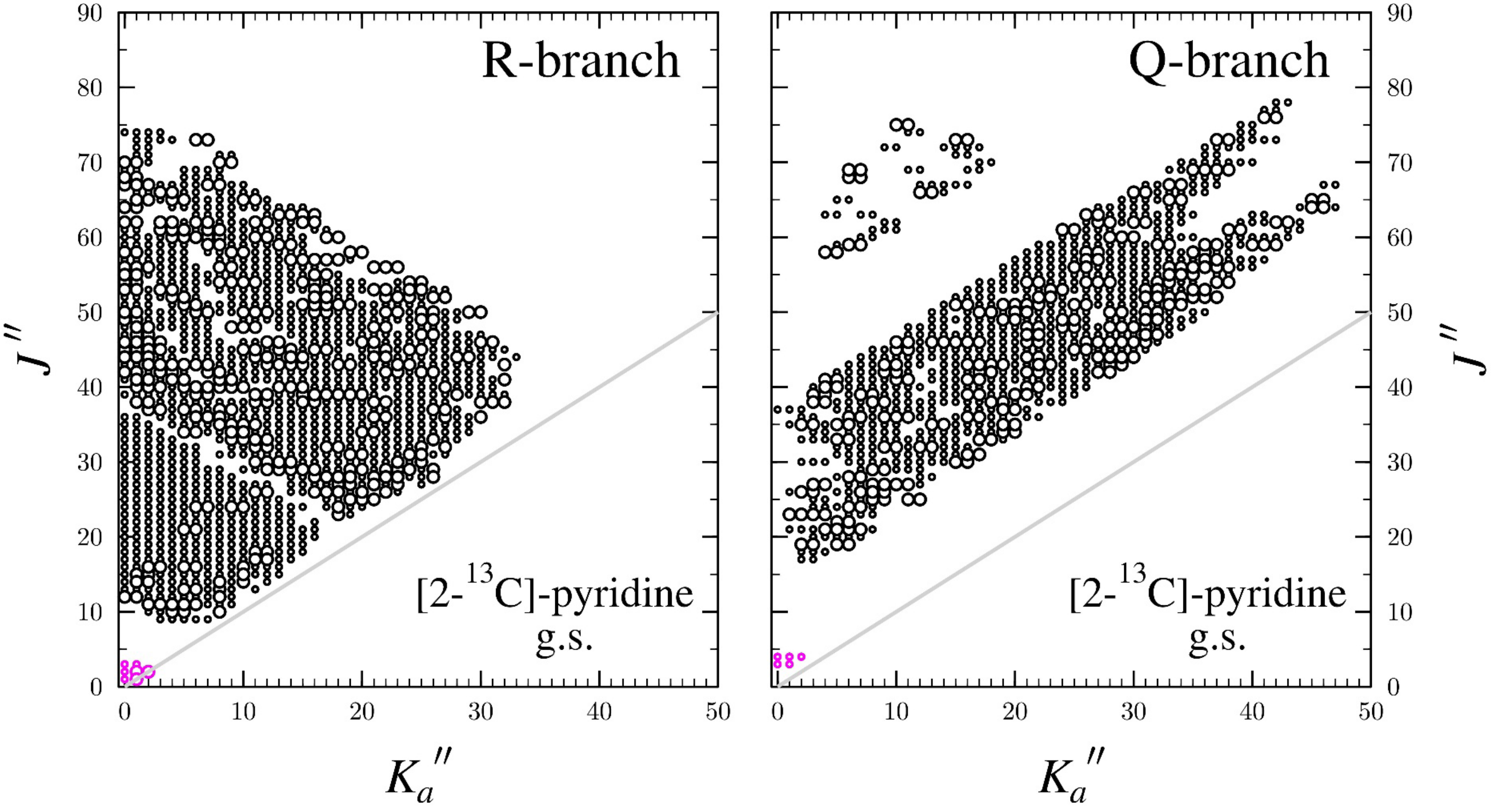

Figure S3. Data distribution plot for the least-squares fit of millimeter-wave spectroscopic data for [2-^{13}C]-pyridine, ground vibrational state. Black circles are measurements from the current work, while magenta circles are from previous work.[8] The size of the outlined circle is proportional to the value of $|(f_{obs.} - f_{calc.})/\delta f|$, where δf is the frequency measurement uncertainty (50 kHz), and no quotient values are larger than three.

Table S4. Experimental and computed spectroscopic constants for [3-^{13}C]-pyridine

	S Reduction, I^r representation			A Reduction, I^r representation	
	Experimental [a]	CCSD(T) [b]		Experimental [a]	CCSD(T) [b]
A_0 (MHz)	5956.582 38 (17)	5936	A_0 (MHz)	5956.581 34 (17)	5936
B_0 (MHz)	5756.031 72 (11)	5723	B_0 (MHz)	5756.033 14 (10)	5723
C_0 (MHz)	2926.629 383 (70)	2913	C_0 (MHz)	2926.628 805 (68)	2913
D_J (kHz)	0.497 265 (64)	0.493	Δ_J (kHz)	0.725 633 (43)	0.714
D_{JK} (kHz)	0.926 84 (42)	0.881	Δ_{JK} (kHz)	−0.443 79 (24)	−0.445
D_K (kHz)	−0.101 26 (44)	−0.057 8	Δ_K (kHz)	1.041 34 (28)	1.047
d_1 (kHz)	−0.307 745 (20)	−0.303	δ_J (kHz)	0.307 722 (19)	0.303
d_2 (kHz)	−0.114 206 (17)	−0.111	δ_K (kHz)	0.521 432 (79)	0.510
H_J (Hz)	−0.000 061 8 (27)	−0.000 0677	Φ_J (Hz)	0.000 310 8 (26)	0.000 305
H_{JK} (Hz)	0.001 758 (26)	0.001 52	Φ_{JK} (Hz)	[−0.000 012 8]	−0.000 012 8
H_{KJ} (Hz)	−0.001 785 (84)	−0.001 47	Φ_{KJ} (Hz)	−0.001 805 (70)	−0.001 94
H_K (Hz)	0.000 405 (73)	0.000 631	Φ_K (Hz)	0.001 988 (71)	0.002 27
h_1 (Hz)	[0.000 114]	0.000 114	ϕ_J (Hz)	[0.000 152]	0.000 152
h_2 (Hz)	[0.000 186]	0.000 186	ϕ_{JK} (Hz)	[0.000 278]	0.000 278
h_3 (Hz)	[0.000 037 9]	0.000 037 9	ϕ_K (Hz)	[0.000 936]	0.000 936
N_{lines} [c]	1569		N_{lines} [c]	1569	
σ_{fit} (MHz)	0.039		σ_{fit} (MHz)	0.039	

[a] Includes transitions from previous work.[8] [b] Evaluated using the cc-pCVTZ basis set. [c] Number of independent transitions.

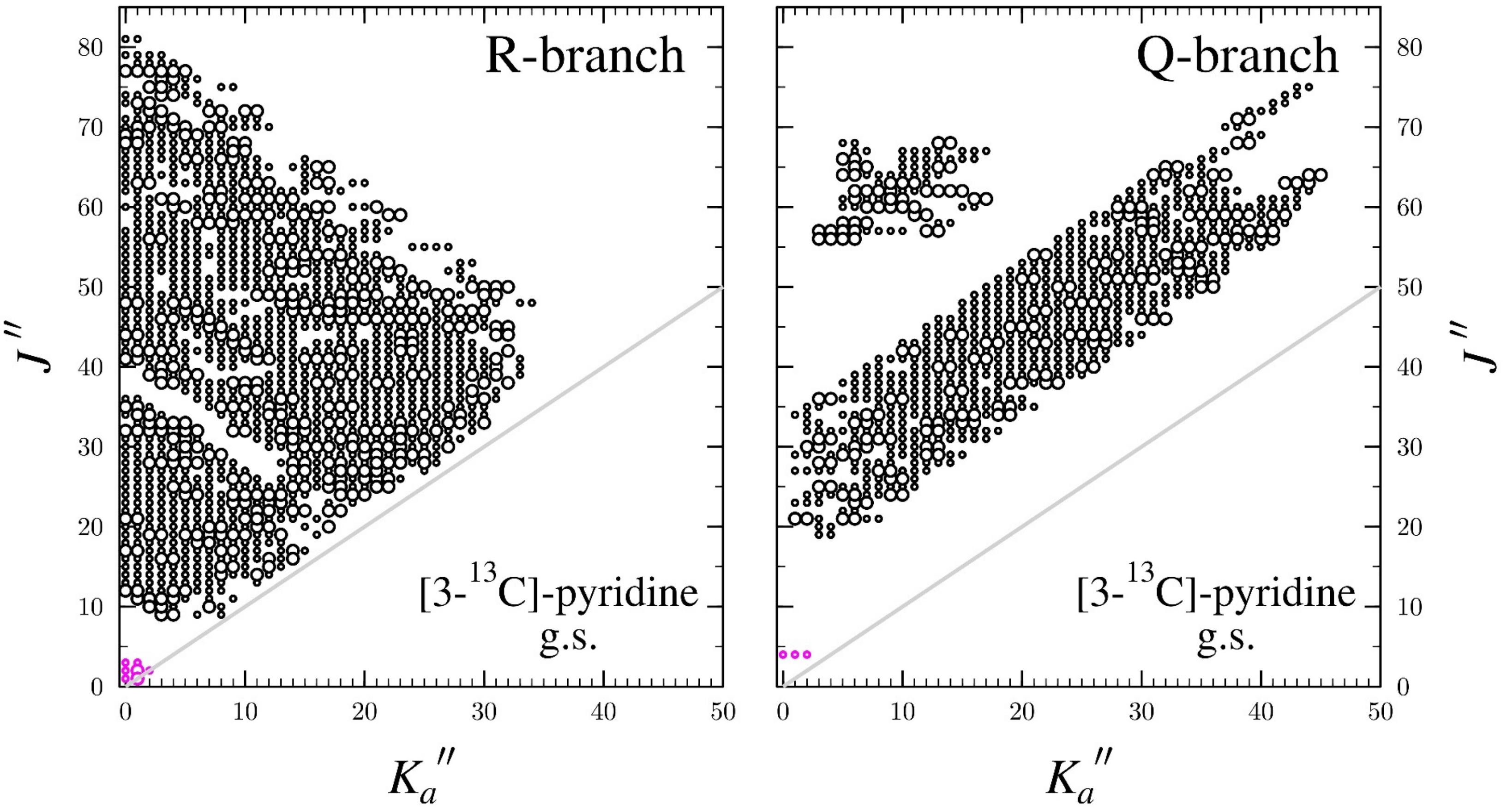

Figure S4. Data distribution plot for the least-squares fit of millimeter-wave spectroscopic data for [3-^{13}C]-pyridine, ground vibrational state. Black circles are measurements from the current work, while magenta circles are from previous work.[8] The size of the outlined circle is proportional to the value of $|(f_{obs.} - f_{calc.})/\delta f|$, where δf is the frequency measurement uncertainty (50 kHz), and no quotient values are larger than three.

Table S5. Experimental and computed spectroscopic constants for [4-^{13}C]-pyridine

	S Reduction, I^r representation			A Reduction, I^r representation	
	Experimental [a]	CCSD(T) [b]		Experimental [a]	CCSD(T) [b]
A_0 (MHz)	6039.477 20 (29)	6020	A_0 (MHz)	6039.476 21 (29)	6020
B_0 (MHz)	5676.042 72 (22)	5642	B_0 (MHz)	5676.044 19 (21)	5642
C_0 (MHz)	2925.395 796 (83)	2912	C_0 (MHz)	2925.395 226 (80)	2912
D_J (kHz)	0.540 782 (100)	0.531	Δ_J (kHz)	0.736 009 (84)	0.721
D_{JK} (kHz)	0.576 15 (64)	0.564	Δ_{JK} (kHz)	−0.594 93 (39)	−0.578
D_K (kHz)	0.259 51 (76)	0.274	Δ_K (kHz)	1.235 22 (50)	1.225
d_1 (kHz)	−0.312 763 (41)	−0.307	δ_J (kHz)	0.312 779 (39)	0.307
d_2 (kHz)	−0.097 598 (28)	−0.0951	δ_K (kHz)	0.493 58 (14)	0.487
H_J (Hz)	0.000 045 8 (36)	0.000 050 7	Φ_J (Hz)	0.000 323 5 (35)	0.000 330
H_{JK} (Hz)	0.001 046 (36)	0.001 09	Φ_{JK} (Hz)	[−0.000 064 8]	−0.000 064 8
H_{KJ} (Hz)	−0.002 42 (18)	−0.002 07	Φ_{KJ} (Hz)	−0.002 81 (17)	−0.002 39
H_K (Hz)	0.002 07 (22)	0.001 64	Φ_K (Hz)	0.003 28 (21)	0.002 85
h_1 (Hz)	[0.000 139]	0.000 139	ϕ_J (Hz)	[0.000 164]	0.000 164
h_2 (Hz)	[0.000 139]	0.000 139	ϕ_{JK} (Hz)	[0.000 286]	0.000 286
h_3 (Hz)	[0.000 0252]	0.000 025 2	ϕ_K (Hz)	[0.000 858]	0.000 858
N_{lines} [c]	1214		N_{lines} [c]	1214	
σ_{fit} (MHz)	0.039		σ_{fit} (MHz)	0.039	

[a] Includes transitions from previous work.[8] [b] Evaluated using the cc-pCVTZ basis set. [c] Number of independent transitions.

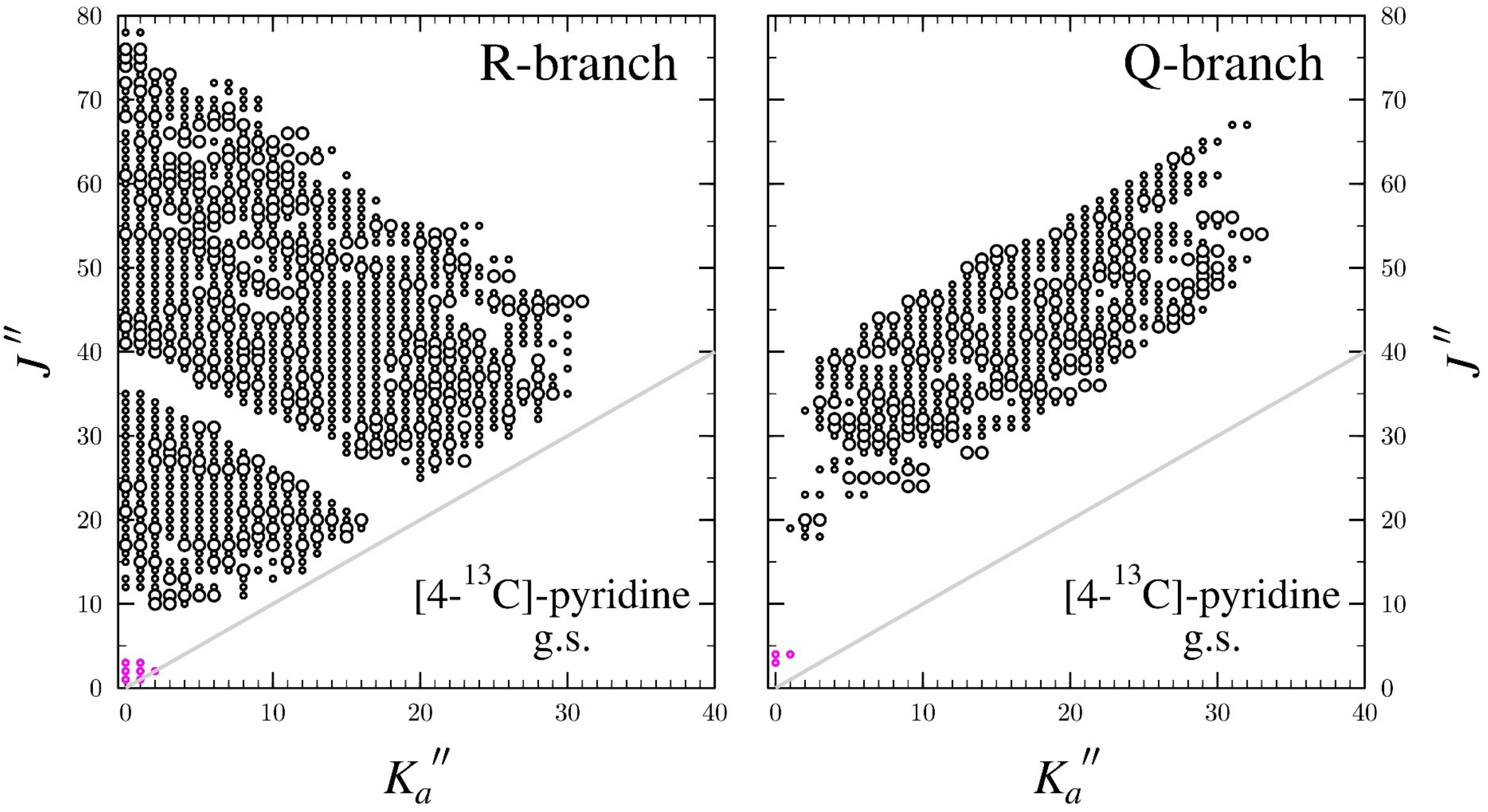

Figure S5. Data distribution plot for the least-squares fit of millimeter-wave spectroscopic data for [4-^{13}C]-pyridine, ground vibrational state. Black circles are measurements from the current work, while magenta circles are from previous work.[8] The size of the outlined circle is proportional to the value of $|(f_{obs.} - f_{calc.})/\delta f|$, where δf is the frequency measurement uncertainty (50 kHz), and no quotient values are larger than three.

Table S6. Experimental and computed spectroscopic constants for [2-^{13}C, ^{15}N]-pyridine

	S Reduction, I^r representation			A Reduction, I^r representation	
	Experimental	CCSD(T) [a]		Experimental	CCSD(T) [a]
A_0 (MHz)	5956.702 52 (14)	5937	A_0 (MHz)	5956.701 44 (16)	5937
B_0 (MHz)	5643.743 04 (11)	5610	B_0 (MHz)	5643.744 42 (12)	5610
C_0 (MHz)	2897.339 457 (58)	2884	C_0 (MHz)	2897.338 80 (12)	2884
D_J (kHz)	0.514 560 (55)	0.506	Δ_J (kHz)	0.717 784 (76)	0.704
D_{JK} (kHz)	0.699 46 (22)	0.675	Δ_{JK} (kHz)	−0.520 06 (17)	−0.510
D_K (kHz)	0.116 27 (20)	0.142	Δ_K (kHz)	1.132 51 (13)	1.13
d_1 (kHz)	−0.304 705 (25)	−0.299	δ_J (kHz)	0.304 714 (26)	0.299
d_2 (kHz)	−0.101 622 2 (76)	−0.098 8	δ_K (kHz)	0.499 169 (37)	0.491
H_J (Hz)	[0.000 028 7]	0.000 028 7	Φ_J (Hz)	0.000 305 (12)	0.000 311
H_{JK} (Hz)	[0.001 10]	0.001 10	Φ_{JK} (Hz)	[−0.000 027 9]	−0.000 027 9
H_{KJ} (Hz)	[−0.001 73]	−0.001 73	Φ_{KJ} (Hz)	[−0.002 21]	−0.002 21
H_K (Hz)	[0.001 263]	0.001 26	Φ_K (Hz)	[0.002 59]	0.002 59
h_1 (Hz)	[0.000 127]	0.000 127	ϕ_J (Hz)	[0.000 155]	0.000 155
h_2 (Hz)	[0.000 141]	0.000 141	ϕ_{JK} (Hz)	[0.000 284]	0.000 284
h_3 (Hz)	[0.000 028 5]	0.000 028 5	ϕ_K (Hz)	[0.000 860]	0.000 860
N_{lines} [b]	940		N_{lines} [b]	940	
σ_{fit} (MHz)	0.037		σ_{fit} (MHz)	0.037	

[a] Evaluated using the cc-pCVTZ basis set. [b] Number of independent transitions.

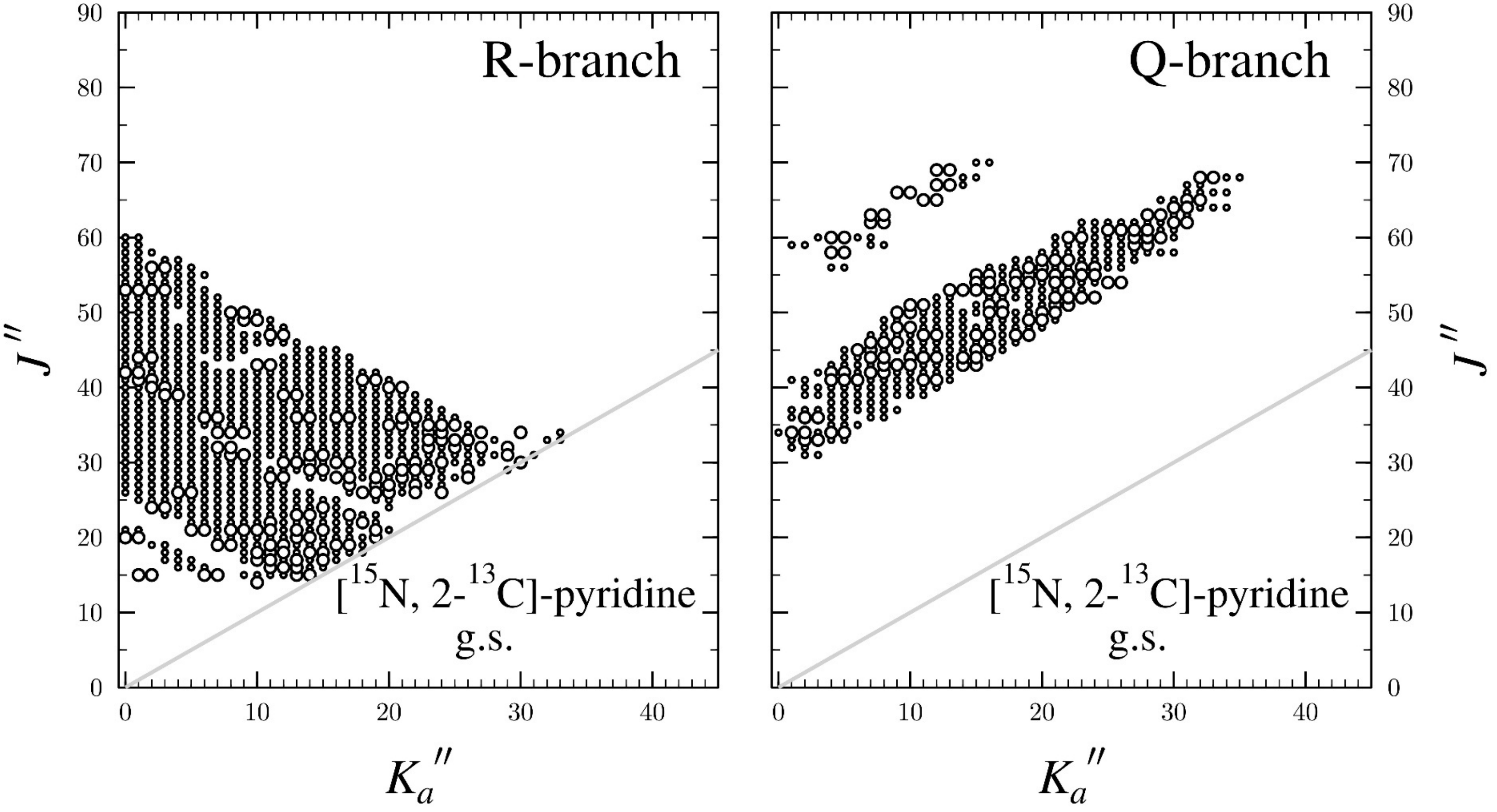

Figure S6. Data distribution plot for the least-squares fit of millimeter-wave spectroscopic data for [2-^{13}C, ^{15}N]-pyridine, ground vibrational state. Black (all) circles are measurements from the current work. The size of the outlined circle is proportional to the value of $|(f_{obs.} - f_{calc.})/\delta f|$, where δf is the frequency measurement uncertainty (50 kHz), and no quotient values are larger than three.

Table S7. Experimental and computed spectroscopic constants for [3-^{13}C, ^{15}N]-pyridine

	S Reduction, I^r representation			A Reduction, I^r representation	
	Experimental	CCSD(T) [a]		Experimental	CCSD(T) [a]
A_0 (MHz)	5950.002 78 (14)	5930	A_0 (MHz)	5950.001 73 (14)	5930
B_0 (MHz)	5638.075 65 (11)	5605	B_0 (MHz)	5638.077 07 (11)	5605
C_0 (MHz)	2894.259 547 (56)	2881	C_0 (MHz)	2894.258 939 (56)	2881
D_J (kHz)	0.511 364 (57)	0.503	Δ_J (kHz)	0.715 553 (53)	0.702
D_{JK} (kHz)	0.716 59 (30)	0.693	Δ_{JK} (kHz)	−0.508 55 (19)	−0.498
D_K (kHz)	0.102 25 (34)	0.126	Δ_K (kHz)	1.123 16 (22)	1.12
d_1 (kHz)	−0.303 586 (26)	−0.298	δ_J (kHz)	0.303 590 (26)	0.298
d_2 (kHz)	−0.102 090 (12)	−0.099 3	δ_K (kHz)	0.501 254 (60)	0.493
H_J (Hz)	[0.000 026 1]	0.000 026 1	Φ_J (Hz)	[0.000 309]	0.000 309
H_{JK} (Hz)	[0.001 08]	0.001 08	Φ_{JK} (Hz)	[−0.000 031 5]	−0.000 031 5
H_{KJ} (Hz)	[−0.001 62]	−0.001 62	Φ_{KJ} (Hz)	[−0.002 15]	−0.002 15
H_K (Hz)	[0.001 17]	0.001 17	Φ_K (Hz)	[0.002 53]	0.002 53
h_1 (Hz)	[0.000 125]	0.000125	ϕ_J (Hz)	[0.000 154]	0.000 154
h_2 (Hz)	[0.000 142]	0.000 142	ϕ_{JK} (Hz)	[0.000 280]	0.000 280
h_3 (Hz)	[0.000 029 4]	0.000 029 4	ϕ_K (Hz)	[0.000 875]	0.000 875
N_{lines} [b]	962		N_{lines} [b]	962	
σ_{fit} (MHz)	0.037		σ_{fit} (MHz)	0.037	

[a] Evaluated using the cc-pCVTZ basis set. [b] Number of independent transitions.

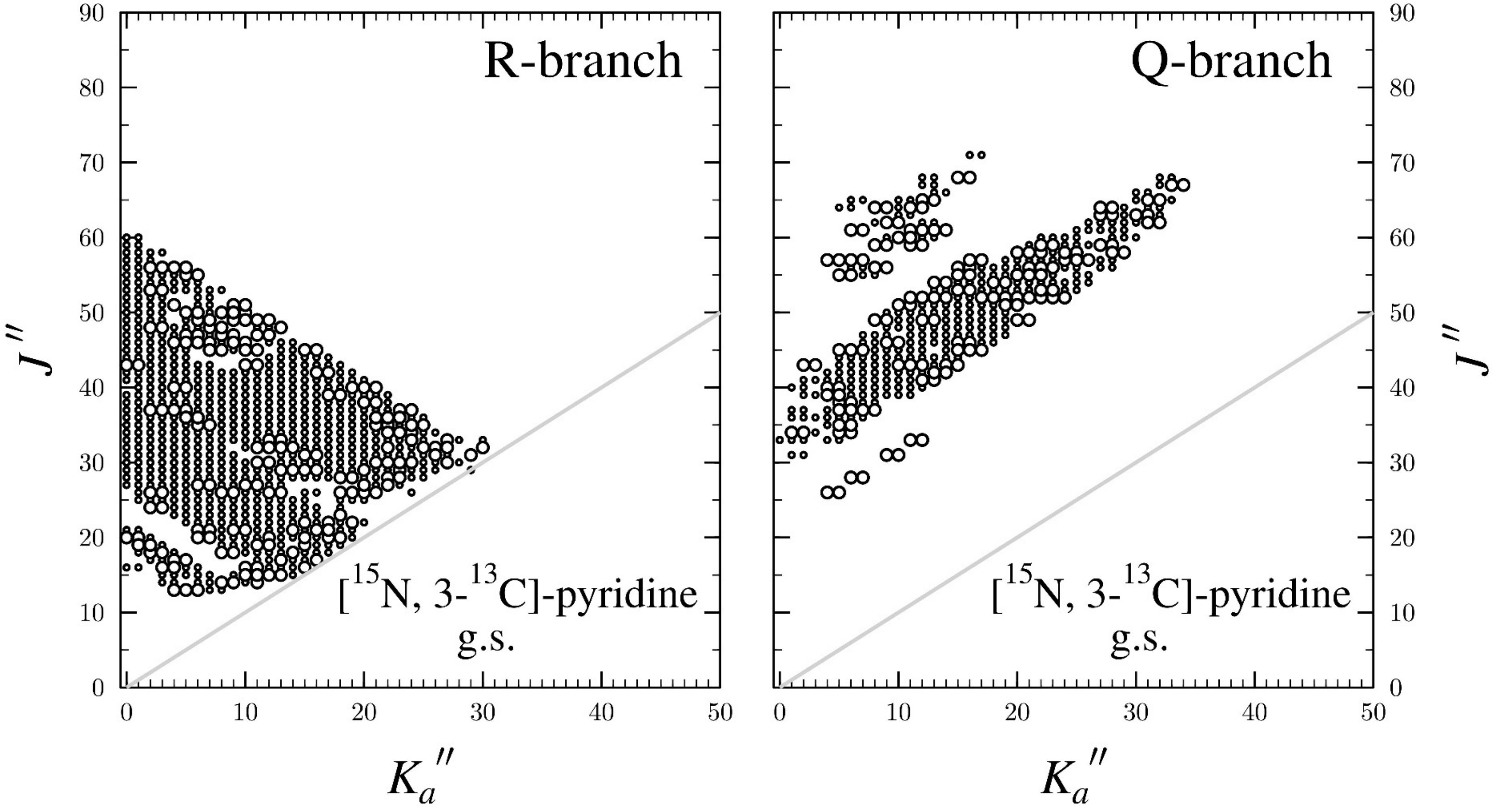

Figure S7. Data distribution plot for the least-squares fit of millimeter-wave spectroscopic data for [3-^{13}C, ^{15}N]-pyridine, ground vibrational state. Black (all) circles are measurements from the current work. The size of the outlined circle is proportional to the value of $|(f_{obs.} - f_{calc.})/\delta f|$, where δf is the frequency measurement uncertainty (50 kHz), and no quotient values are larger than three.

Table S8. Experimental and computed spectroscopic constants for [4-^{13}C, ^{15}N]-pyridine

	S Reduction, I^r representation			A Reduction, I^r representation	
	Experimental [a]	CCSD(T) [b]		Experimental [a]	CCSD(T) [b]
A_0 (MHz)	6039.650 45 (41)	6020	A_0 (MHz)	6039.649 49 (41)	6020
B_0 (MHz)	5553.927 52 (29)	5520	B_0 (MHz)	5553.928 89 (29)	5520
C_0 (MHz)	2892.646 934 (65)	2879	C_0 (MHz)	2892.646 293 (65)	2879
D_J (kHz)	0.533 80 (17)	0.524	Δ_J (kHz)	0.715 82 (16)	0.701
D_{JK} (kHz)	0.507 0 (13)	0.493	Δ_{JK} (kHz)	−0.585 06 (71)	−0.570
D_K (kHz)	0.333 5 (19)	0.352	Δ_K (kHz)	1.243 4 (13)	1.24
d_1 (kHz)	−0.303 501 (80)	−0.297	δ_J (kHz)	0.303 508 (80)	0.297
d_2 (kHz)	−0.091 005 (60)	−0.089	δ_K (kHz)	0.496 96 (33)	0.490
H_J (Hz)	[0.000 0697]	0.000 069 7	Φ_J (Hz)	[0.000 323]	0.000 323
H_{JK} (Hz)	[0.000 9]	0.000 9	Φ_{JK} (Hz)	[−0.000 085 5]	−0.000 085 5
H_{KJ} (Hz)	[−0.001 82]	−0.001 82	Φ_{KJ} (Hz)	[−0.002 33]	−0.002 33
H_K (Hz)	[0.001 57]	0.001 57	Φ_K (Hz)	[0.002 81]	0.002 81
h_1 (Hz)	[0.000 137]	0.000 137	ϕ_J (Hz)	[0.000 161]	0.000 161
h_2 (Hz)	[0.000 126]	0.000 126	ϕ_{JK} (Hz)	[0.000 277]	0.000 277
h_3 (Hz)	[0.000 024]	0.000 024	ϕ_K (Hz)	[0.000 902]	0.000 902
N_{lines} [c]	665		N_{lines} [c]	665	
σ_{fit} (MHz)	0.039		σ_{fit} (MHz)	0.040	

[a] Includes transitions from previous work.[9] [b] Evaluated using the cc-pCVTZ basis set. [c] Number of independent transitions.

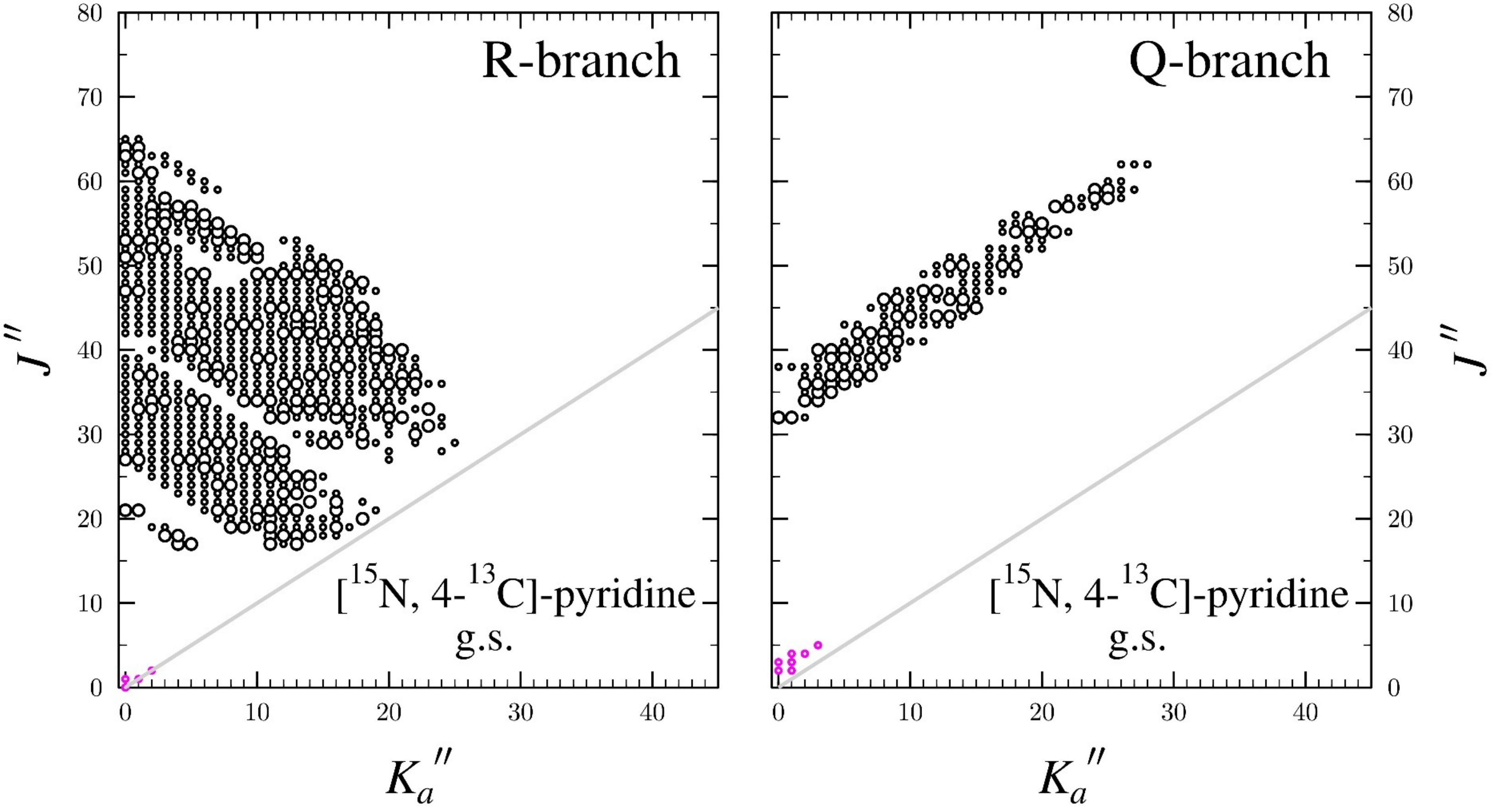

Figure S8. Data distribution plot for the least-squares fit of millimeter-wave spectroscopic data for [4-^{13}C, ^{15}N]-pyridine, ground vibrational state. Black circles are measurements from the current work, while magenta circles are from previous work.[9] The size of the outlined circle is proportional to the value of $|(f_{obs.} - f_{calc.})/\delta f|$, where δf is the frequency measurement uncertainty (50 kHz), and no quotient values are larger than three.

Table S9. Experimental and computed spectroscopic constants for [2-^{2}H]-pyridine

	S Reduction, I^r representation			A Reduction, I^r representation	
	Experimental [a]	CCSD(T) [b]		Experimental [a]	CCSD(T) [b]
A_0 (MHz)	5900.879 02 (16)	5875.1	A_0 (MHz)	5900.878 036 (93)	5875.1
B_0 (MHz)	5558.517 215 (94)	5532.4	B_0 (MHz)	5558.518 673 (85)	5532.4
C_0 (MHz)	2861.711 19 (10)	2848.5	C_0 (MHz)	2861.710 557 (93)	2848.5
D_J (kHz)	0.395 669 (92)	0.389	Δ_J (kHz)	0.616 141 (66)	0.607
D_{JK} (kHz)	1.188 50 (50)	1.183	Δ_{JK} (kHz)	−0.134 65 (24)	−0.126
D_K (kHz)	−0.307 93 (47)	−0.317	Δ_K (kHz)	0.795 33 (30)	0.773
d_1 (kHz)	−0.256 534 (35)	−0.253	δ_J (kHz)	0.256 471 (23)	0.253
d_2 (kHz)	−0.110 283 (18)	−0.109	δ_K (kHz)	0.552 970 (83)	0.548
H_J (Hz)	[−0.000 144]	−0.000 144	Φ_J (Hz)	[0.000 225]	0.000 225
H_{JK} (Hz)	0.001 488 (81)	0.001 34	Φ_{JK} (Hz)	[0.000 049 9]	0.000 049 9
H_{KJ} (Hz)	[0.000 045 2]	0.000 045 2	Φ_{KJ} (Hz)	[−0.001 20]	−0.001 20
H_K (Hz)	−0.001 407 (67)	−0.000 727	Φ_K (Hz)	[0.001 43]	0.001 43
h_1 (Hz)	[0.000 065 9]	0.000 065 9	ϕ_J (Hz)	[0.000 112]	0.000 112
h_2 (Hz)	[0.000 184]	0.000 184	ϕ_{JK} (Hz)	[0.000 246]	0.000 246
h_3 (Hz)	[0.000 046]	0.000 046	ϕ_K (Hz)	[0.001 04]	0.001 04
N_{lines} [c]	998		N_{lines} [c]	998	
σ_{fit} (MHz)	0.033		σ_{fit} (MHz)	0.033	

[a] Includes transitions from previous work.[10] [b] Evaluated using the cc-pCVTZ basis set. [c] Number of independent transitions.

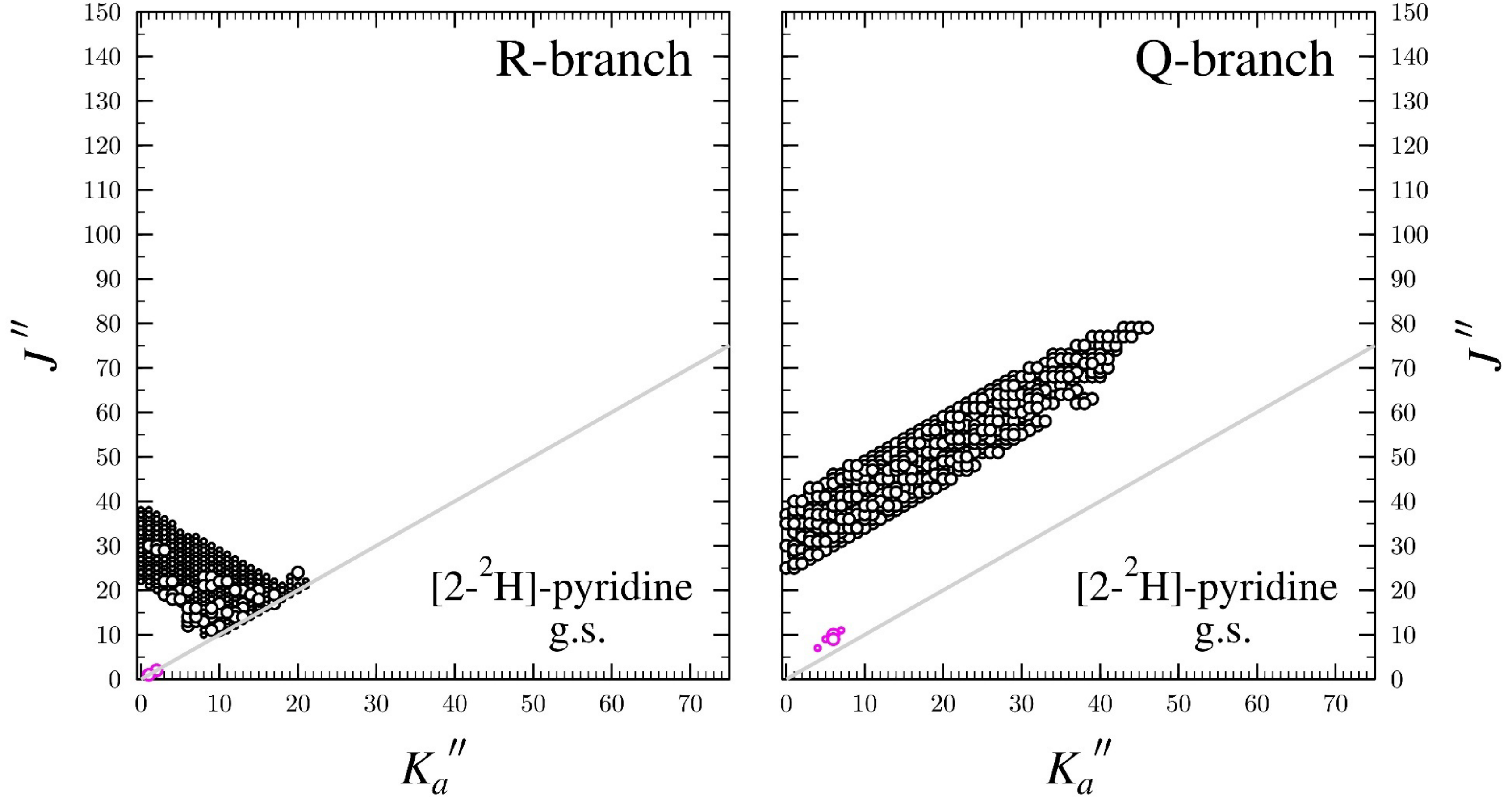

Figure S9. Data distribution plot for the least-squares fit of millimeter-wave spectroscopic data for [2-^{2}H]-pyridine, ground vibrational state. Black circles are measurements from the current work, while magenta circles are from previous work.[10] The size of the outlined circle is proportional to the value of $|(f_{obs.} - f_{calc.})/\delta f|$, where δf is the frequency measurement uncertainty (50 kHz), and no quotient values are larger than three.

Table S10. Experimental and computed spectroscopic constants for [3-^{2}H]-pyridine

	S Reduction, I^r representation			A Reduction, I^r representation	
	Experimental [a]	CCSD(T) [b]		Experimental [a]	CCSD(T) [b]
A_0 (MHz)	5889.188 821 (70)	5863	A_0 (MHz)	5889.187 708 (70)	5863
B_0 (MHz)	5555.047 395 (57)	5529	B_0 (MHz)	5555.048 951 (57)	5529
C_0 (MHz)	2858.029 053 (57)	2845	C_0 (MHz)	2858.028 385 (57)	2845
D_J (kHz)	0.389 377 (41)	0.383	Δ_J (kHz)	0.612 258 (28)	0.604
D_{JK} (kHz)	1.224 02 (28)	1.22	Δ_{JK} (kHz)	−0.113 32 (16)	−0.103
D_K (kHz)	−0.336 44 (30)	−0.352	Δ_K (kHz)	0.778 05 (21)	0.752
d_1 (kHz)	−0.254 589 (13)	−0.251	δ_J (kHz)	0.254 591 (13)	0.251
d_2 (kHz)	−0.111 439 (11)	−0.110	δ_K (kHz)	0.556 184 (57)	0.552
H_J (Hz)	−0.000 185 (23)	−0.000 169	Φ_J (Hz)	0.000 148 8 (91)	0.000 215
H_{JK} (Hz)	0.001 69 (19)	0.001 61	Φ_{JK} (Hz)	0.000 65 (10)	0.000 182
H_{KJ} (Hz)	−0.000 81 (20)	−0.000 551	Φ_{KJ} (Hz)	−0.002 46 (17)	−0.001 54
H_K (Hz)	[−0.000 361]	−0.000 361	Φ_K (Hz)	0.002 04 (11)	0.001 67
h_1 (Hz)	0.000 032 7 (46)	0.000 063 3	ϕ_J (Hz)	0.000 078 2 (46)	0.000 107
h_2 (Hz)	0.000 166 9 (73)	0.000 192	ϕ_{JK} (Hz)	0.000 448 (38)	0.000 298
h_3 (Hz)	0.000 045 69 (29)	0.000 043 6	ϕ_K (Hz)	0.000 924 (38)	0.000 951
N_{lines} [c]	2437		N_{lines} [c]	2437	
σ_{fit} (MHz)	0.036		σ_{fit} (MHz)	0.036	

[a] Includes transitions from previous work.[11] [b] Evaluated using the cc-pCVTZ basis set. [c] Number of independent transitions.

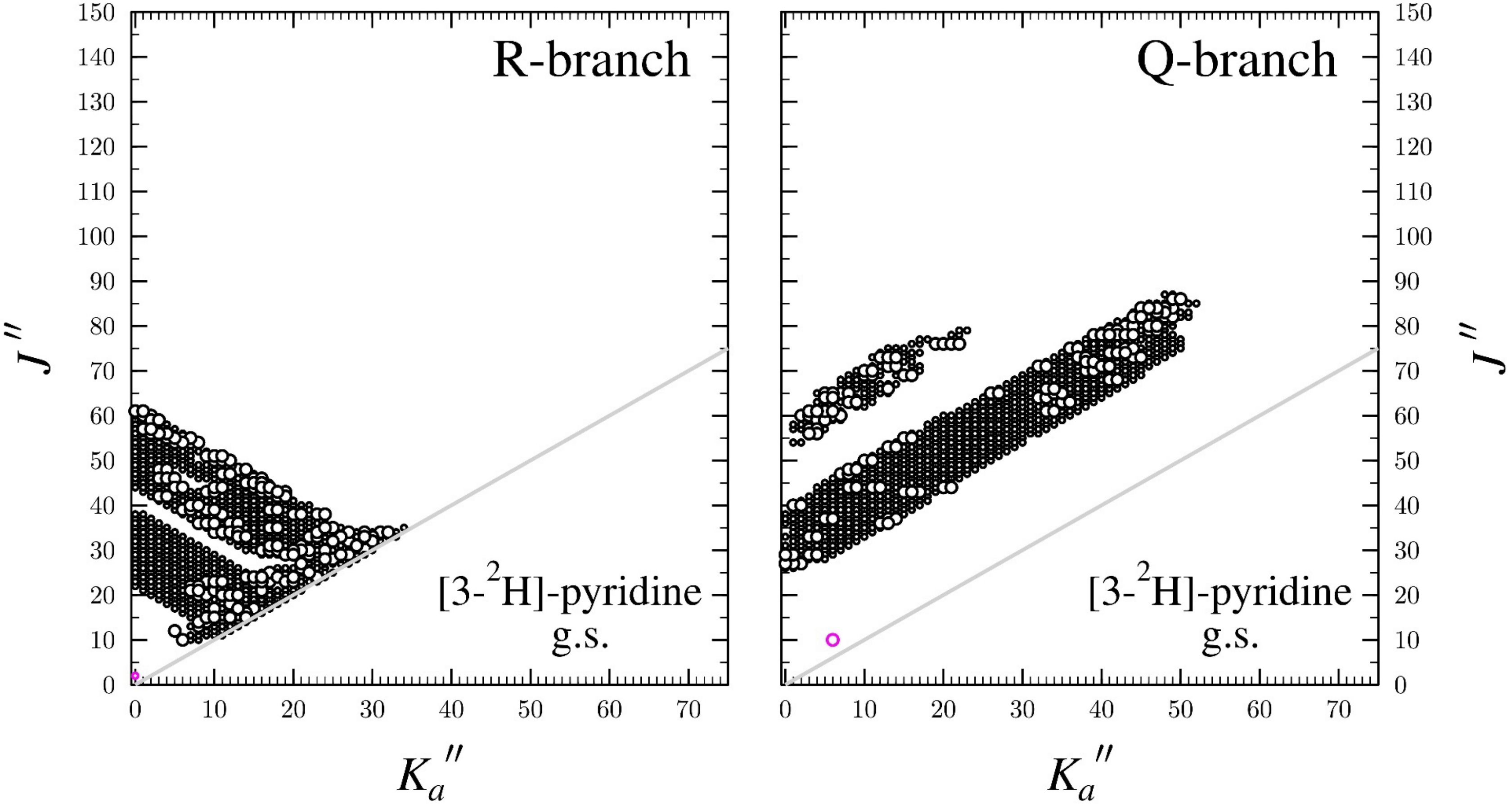

Figure S10. Data distribution plot for the least-squares fit of millimeter-wave spectroscopic data for [3-^{2}H]-pyridine, ground vibrational state. Black circles are measurements from the current work, while magenta circles are from previous work.[11] The size of the outlined circle is proportional to the value of $|(f_{obs.} - f_{calc.})/\delta f|$, where δf is the frequency measurement uncertainty (50 kHz), and no quotient values are larger than three.

Table S11. Experimental and computed spectroscopic constants for [4-^{2}H]-pyridine

	S Reduction, I^r representation			A Reduction, I^r representation	
	Experimental [a]	CCSD(T) [b]		Experimental [a]	CCSD(T) [b]
A_0 (MHz)	6038.998 56 (20)	6020	A_0 (MHz)	6038.997 82 (19)	6020
B_0 (MHz)	5420.072 68 (14)	5388	B_0 (MHz)	5420.074 00 (14)	5388
C_0 (MHz)	2855.823 33 (21)	2843	C_0 (MHz)	2855.822 72 (21)	2843
D_J (kHz)	0.487 839 (53)	0.479	Δ_J (kHz)	0.649 594 (56)	0.637
D_{JK} (kHz)	0.482 104 (92)	0.467	Δ_{JK} (kHz)	−0.488 321 (64)	−0.477
D_K (kHz)	0.404 80 (15)	0.422	Δ_K (kHz)	1.213 53 (15)	1.209
d_1 (kHz)	−0.272 830 7 (89)	−0.268	δ_J (kHz)	0.272 832 7 (88)	0.268
d_2 (kHz)	−0.080 868 1 (65)	−0.078 7	δ_K (kHz)	0.479 701 (24)	0.472
H_J (Hz)	0.000 078 9 (53)	0.000 060 1	Φ_J (Hz)	0.000 287 4 (51)	0.000267
H_{JK} (Hz)	0.000 702 (25)	0.000 698	Φ_{JK} (Hz)	[−0.000 034 7]	−0.0000347
H_{KJ} (Hz)	−0.001 450 (61)	−0.001 39	Φ_{KJ} (Hz)	−0.002 072 (28)	−0.00205
H_K (Hz)	0.001 470 (58)	0.001 35	Φ_K (Hz)	0.002 633 (56)	0.00254
h_1 (Hz)	[0.000 112]	0.000 112	ϕ_J (Hz)	[0.000 133]	0.000133
h_2 (Hz)	[0.000 103]	0.000 103	ϕ_{JK} (Hz)	[0.000 252]	0.000252
h_3 (Hz)	[0.000 021 1]	0.000 021 1	ϕ_K (Hz)	[0.000 885]	0.000885
N_{lines} [c]	830		N_{lines} [c]	830	
σ_{fit} (MHz)	0.032		σ_{fit} (MHz)	0.032	

[a] Includes transitions from previous work.[12] [b] Evaluated using the cc-pCVTZ basis set. [c] Number of independent transitions.

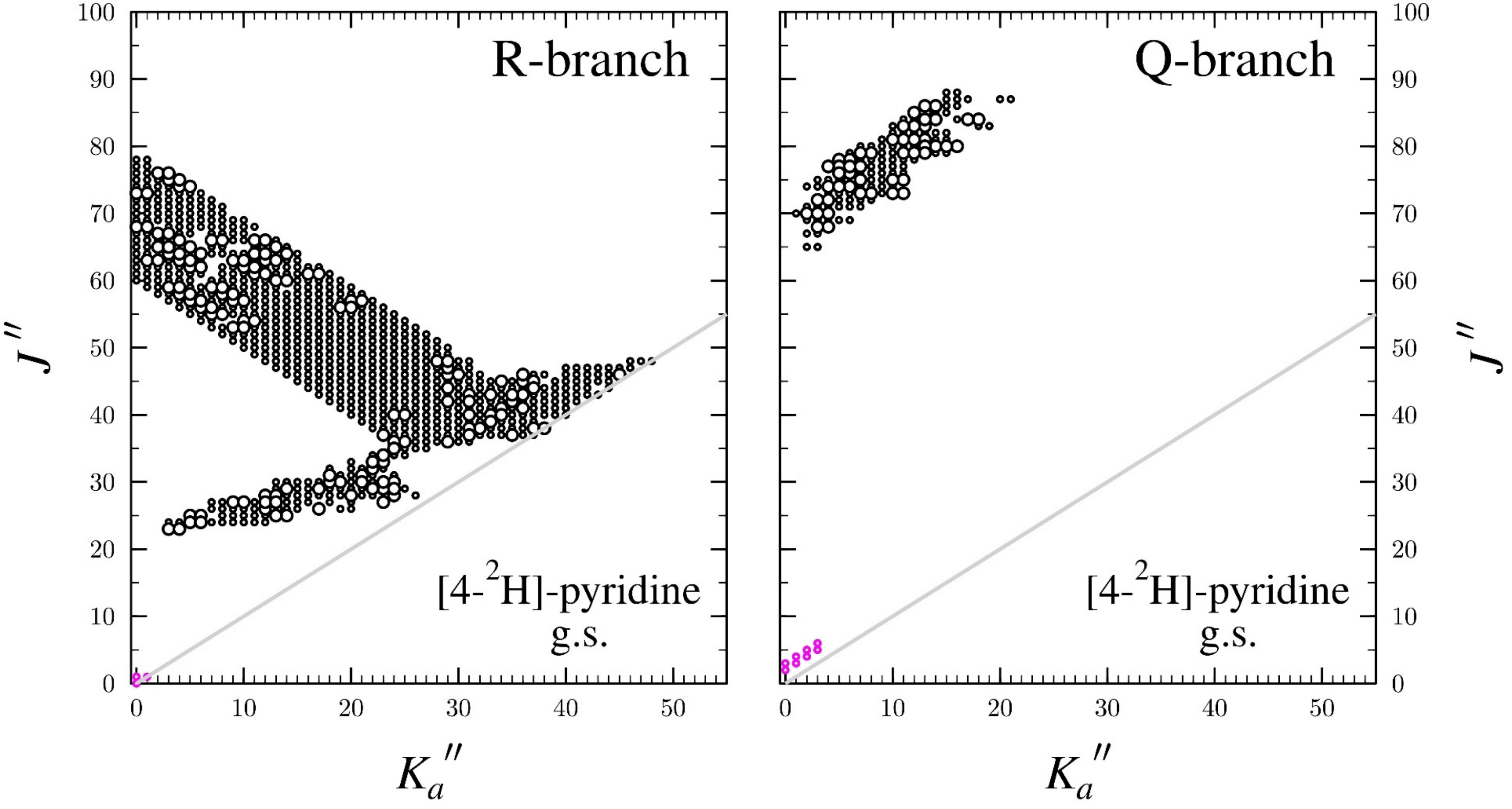

Figure S11. Data distribution plot for the least-squares fit of millimeter-wave spectroscopic data for [4-^{2}H]-pyridine, ground vibrational state. Black circles are measurements from the current work, while magenta circles are from previous work.[12] The size of the outlined circle is proportional to the value of $|(f_{obs.} - f_{calc.})/\delta f|$, where δf is the frequency measurement uncertainty (50 kHz), and no quotient values are larger than three.

Table S12. Experimental and computed spectroscopic constants for [3-^{2}H, 2-^{13}C]-pyridine

	S Reduction, I^{r} representation			A Reduction, I^{r} representation	
	Experimental	CCSD(T) [a]		Experimental	CCSD(T) [a]
A_0 (MHz)	5778.40 (14)	5752	A_0 (MHz)	5778.39 (14)	5752
B_0 (MHz)	5550.26 (13)	5525	B_0 (MHz)	5550.27 (13)	5525
C_0 (MHz)	2830.427 22 (62)	2817	C_0 (MHz)	2830.426 68 (62)	2817
D_J (kHz)	0.380 55 (27)	0.378	Δ_J (kHz)	0.600 98 (27)	0.599
D_{JK} (kHz)	1.186 3 (35)	1.18	Δ_{JK} (kHz)	−0.134 3 (35)	−0.144
D_K (kHz)	−0.354 (17)	−0.324	Δ_K (kHz)	0.744 (17)	0.778
d_1 (kHz)	[−0.250]	−0.250	δ_J (kHz)	[0.250]	0.250
d_2 (kHz)	[−0.110]	−0.110	δ_K (kHz)	[0.515]	0.515
H_J (Hz)	[−0.000 184]	−0.000 184	Φ_J (Hz)	[0.000 205]	0.000 205
H_{JK} (Hz)	[0.001 88]	0.001 88	Φ_{JK} (Hz)	[0.000 259]	0.000 259
H_{KJ} (Hz)	[−0.001 41]	−0.001 41	Φ_{KJ} (Hz)	[−0.001 83]	−0.001 83
H_K (Hz)	[0.000 274]	0.000 274	Φ_K (Hz)	[0.001 93]	0.001 93
h_1 (Hz)	[0.000 064 2]	0.000 064 2	ϕ_J (Hz)	[0.000 102]	0.000 102
h_2 (Hz)	[0.000 194]	0.000 194	ϕ_{JK} (Hz)	[0.000 32]	0.000 32
h_3 (Hz)	[0.000 037 9]	0.000 037 9	ϕ_K (Hz)	[0.000 774]	0.000 774
N_{lines} [b]	84		N_{lines} [b]	84	
σ_{fit} (MHz)	0.044		σ_{fit} (MHz)	0.044	

[a] Evaluated using the cc-pCVTZ basis set. [b] Number of independent transitions.

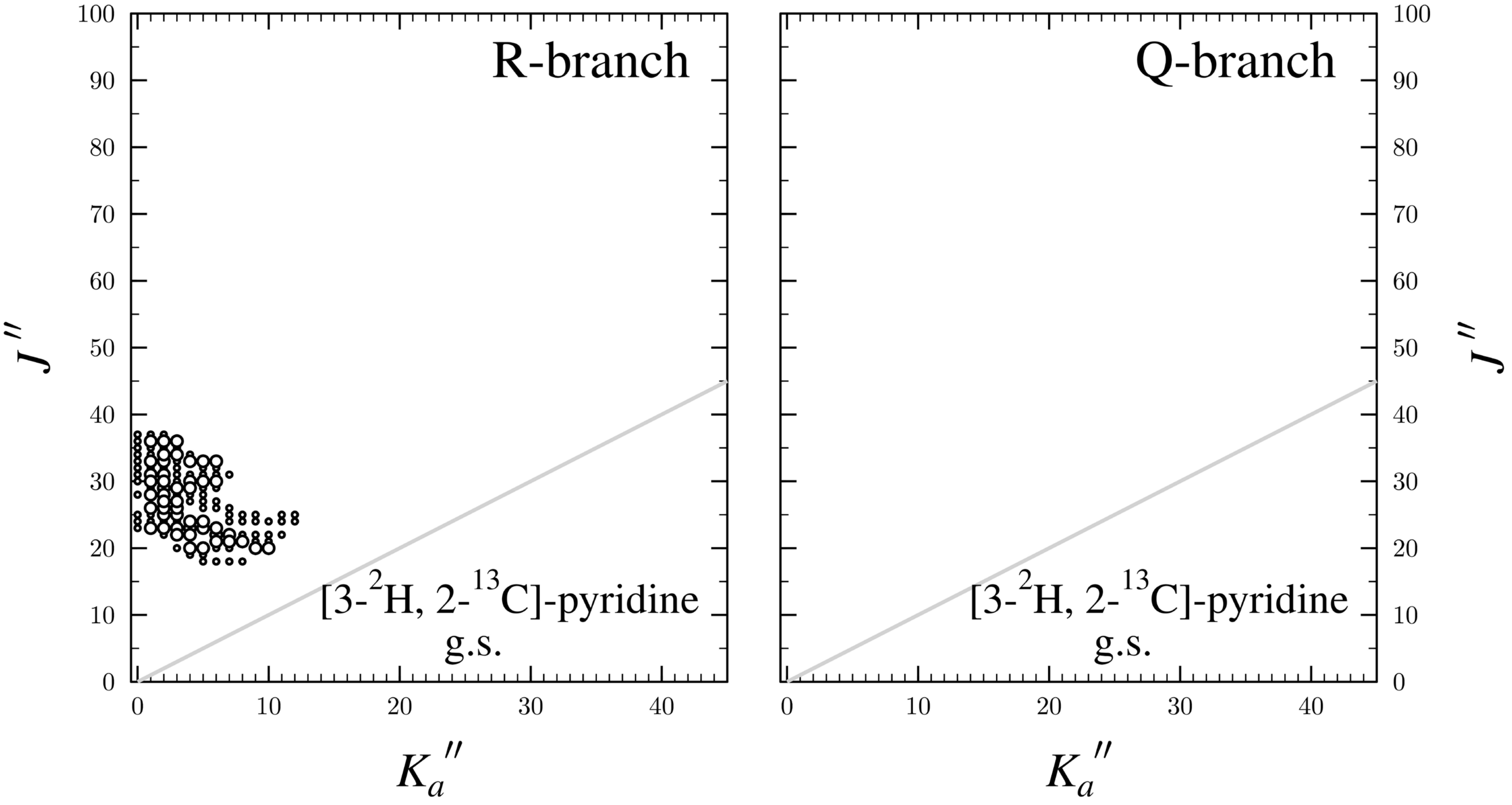

Figure S12. Data distribution plot for the least-squares fit of millimeter-wave spectroscopic data for [3-^{2}H, 2-^{13}C]-pyridine, ground vibrational state. Black (all) circles are measurements from the current work. The size of the outlined circle is proportional to the value of $|(f_{obs.} - f_{calc.})/\delta f|$, where δf is the frequency measurement uncertainty (50 kHz), and no quotient values are larger than three.

Table S13. Experimental and computed spectroscopic constants for [3-^{2}H, 3-^{13}C]-pyridine

	S Reduction, I^r representation			A Reduction, I^r representation	
	Experimental	CCSD(T) [a]		Experimental	CCSD(T) [a]
A_0 (MHz)	5882.13 (11)	5855	A_0 (MHz)	5881.932 (92)	5855
B_0 (MHz)	5452.449 (95)	5428	B_0 (MHz)	5452.613 (85)	5428
C_0 (MHz)	2828.976 88 (83)	2816	C_0 (MHz)	2828.977 02 (84)	2816
D_J (kHz)	0.383 89 (37)	0.382	Δ_J (kHz)	0.589 44 (39)	0.588
D_{JK} (kHz)	1.136 1 (46)	1.13	Δ_{JK} (kHz)	−0.087 8 (42)	−0.107
D_K (kHz)	−0.193 (15)	−0.243	Δ_K (kHz)	[0.784]	0.784
d_1 (kHz)	[−0.244]	−0.244	δ_J (kHz)	[0.244]	0.244
d_2 (kHz)	[−0.103]	−0.103	δ_K (kHz)	[0.546]	0.546
H_J (Hz)	[−0.000 140]	−0.000 140	Φ_J (Hz)	[0.000 203]	0.000 203
H_{JK} (Hz)	[0.001 63]	0.001 63	Φ_{JK} (Hz)	[0.000 310]	0.000 310
H_{KJ} (Hz)	[−0.001 21]	−0.001 21	Φ_{KJ} (Hz)	[−0.001 97]	−0.001 97
H_K (Hz)	[0.000 287]	0.000 287	Φ_K (Hz)	[0.002 02]	0.002 02
h_1 (Hz)	[0.000 066 3]	0.000 066 3	ϕ_J (Hz)	[0.000 101]	0.000 101
h_2 (Hz)	[0.000 172]	0.000 172	ϕ_{JK} (Hz)	[0.000 351]	0.000 351
h_3 (Hz)	[0.000 035]	0.000 035	ϕ_K (Hz)	[0.000 852]	0.000 852
N_{lines} [b]	76		N_{lines} [b]	76	
σ_{fit} (MHz)	0.043		σ_{fit} (MHz)	0.046	

[a] Evaluated using the cc-pCVTZ basis set. [b] Number of independent transitions.

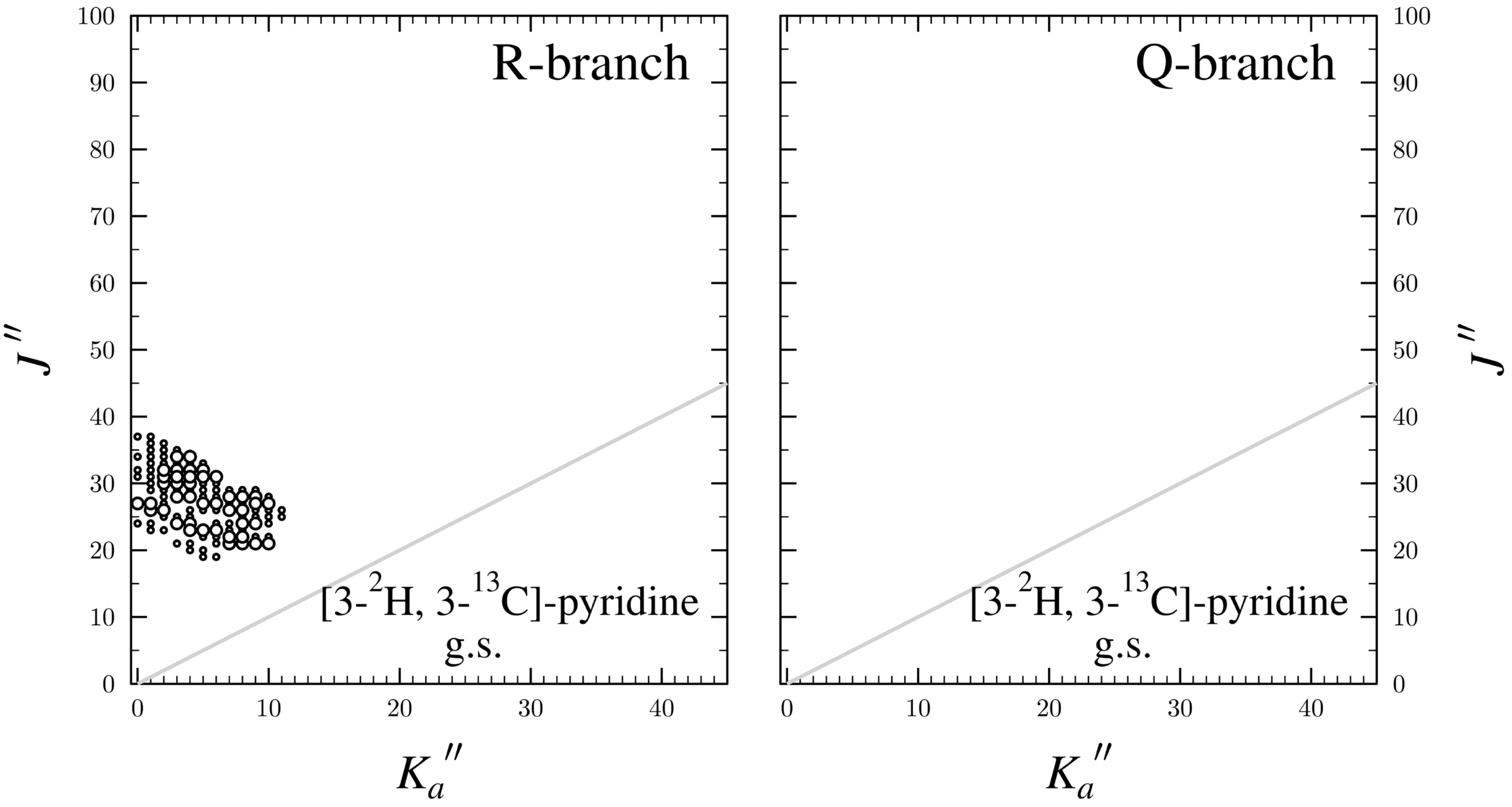

Figure S13. Data distribution plot for the least-squares fit of millimeter-wave spectroscopic data for [3-^{2}H, 3-^{13}C]-pyridine, ground vibrational state. Black (all) circles are measurements from the current work. The size of the outlined circle is proportional to the value of $|(f_{obs.} - f_{calc.})/\delta f|$, where δf is the frequency measurement uncertainty (50 kHz), and no quotient values are larger than three.

Table S14. Experimental and computed spectroscopic constants for [3-^{2}H, 4-^{13}C]-pyridine

	S Reduction, I^r representation			A Reduction, I^r representation	
	Experimental	CCSD(T) [a]		Experimental	CCSD(T) [a]
A_0 (MHz)	5835.742 (16)	5812	A_0 (MHz)	5835.739 (16)	5812
B_0 (MHz)	5485.943 (17)	5458	B_0 (MHz)	5485.946 (17)	5458
C_0 (MHz)	2827.127 76 (66)	2814	C_0 (MHz)	2827.127 15 (66)	2814
D_J (kHz)	0.383 90 (28)	0.382	Δ_J (kHz)	0.601 52 (28)	0.600
D_{JK} (kHz)	1.201 2 (25)	1.195	Δ_{JK} (kHz)	−0.097 4 (25)	−0.110
D_K (kHz)	−0.330 3 (86)	−0.374	Δ_K (kHz)	0.745 2 (86)	0.714
d_1 (kHz)	[−0.250]	−0.250	δ_J (kHz)	[0.250]	0.250
d_2 (kHz)	[−0.109]	−0.109	δ_K (kHz)	[0.553]	0.553
H_J (Hz)	[−0.000 152]	−0.000 152	Φ_J (Hz)	[0.000 220]	0.000 220
H_{JK} (Hz)	[0.001 28]	0.001 28	Φ_{JK} (Hz)	[0.000 039]	0.000 039
H_{KJ} (Hz)	[0.000 418]	0.000 418	Φ_{KJ} (Hz)	[−0.001 02]	−0.001 02
H_K (Hz)	[−0.001 11]	−0.001 11	Φ_K (Hz)	[0.001 20]	0.001 20
h_1 (Hz)	[0.000 060 9]	0.000 060 9	ϕ_J (Hz)	[0.000 110]	0.000 110
h_2 (Hz)	[0.000 186]	0.000 186	ϕ_{JK} (Hz)	[0.000 233]	0.000 233
h_3 (Hz)	[0.000 048 6]	0.000 048 6	ϕ_K (Hz)	[0.001 09]	0.001 09
N_{lines} [b]	108		N_{lines} [b]	108	
σ_{fit} (MHz)	0.043		σ_{fit} (MHz)	0.043	

[a] Evaluated using the cc-pCVTZ basis set. [b] Number of independent transitions.

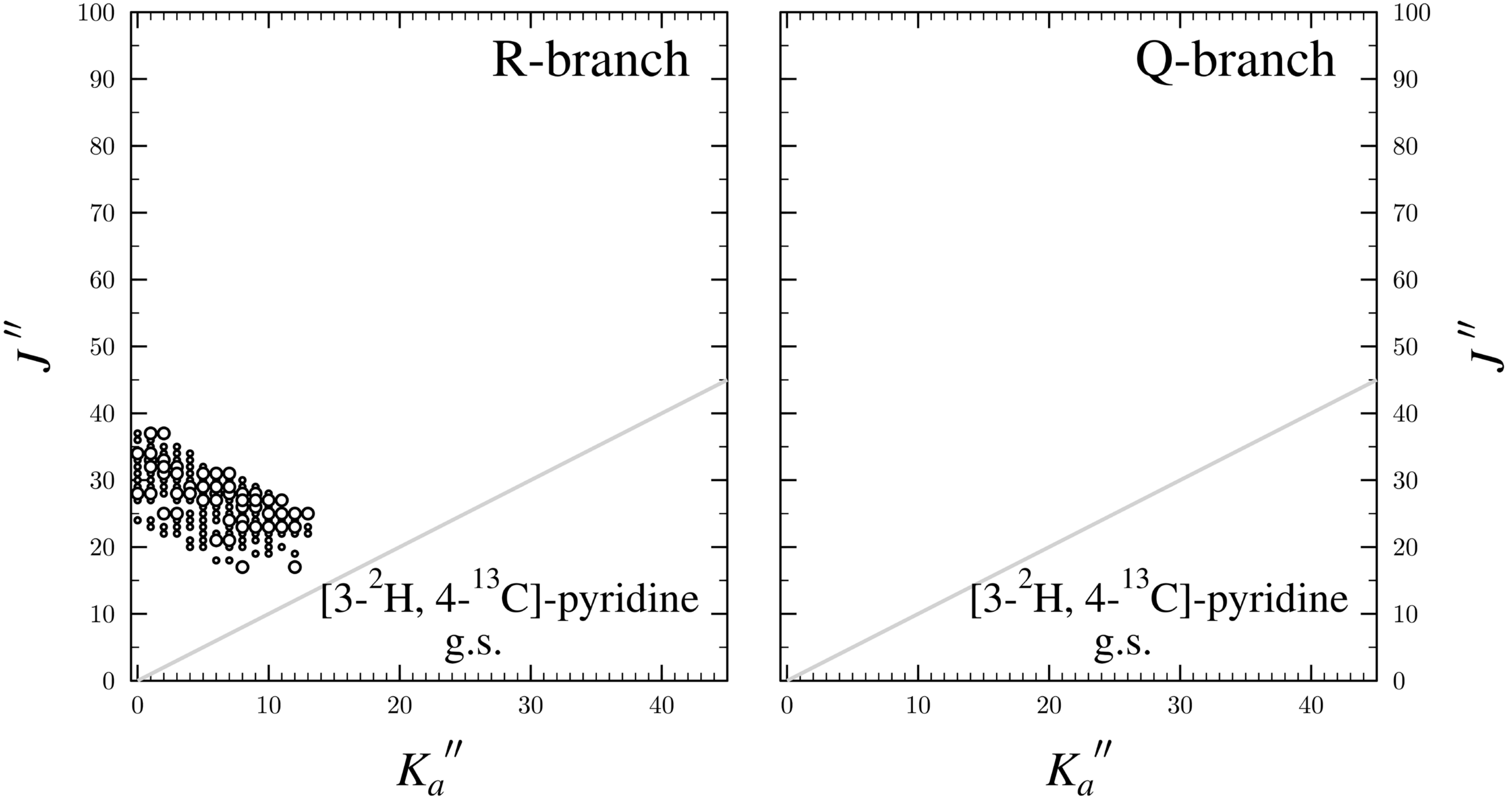

Figure S14. Data distribution plot for the least-squares fit of millimeter-wave spectroscopic data for [3-^{2}H, 4-^{13}C]-pyridine, ground vibrational state. Black (all) circles are measurements from the current work. The size of the outlined circle is proportional to the value of $|(f_{obs.} - f_{calc.})/\delta f|$, where δf is the frequency measurement uncertainty (50 kHz), and no quotient values are larger than three.

Table S15. Experimental and computed spectroscopic constants for [3-^{2}H, 5-^{13}C]-pyridine

	S Reduction, I^{r} representation			A Reduction, I^{r} representation	
	Experimental	CCSD(T) [a]		Experimental	CCSD(T) [a]
A_0 (MHz)	5770.615 (53)	5743	A_0 (MHz)	5770.607 (53)	5743
B_0 (MHz)	5543.966 (55)	5520	B_0 (MHz)	5543.974 (55)	5520
C_0 (MHz)	2826.924 86 (63)	2814	C_0 (MHz)	2826.924 35 (64)	2814
D_J (kHz)	0.379 70 (31)	0.378	Δ_J (kHz)	0.599 18 (31)	0.597
D_{JK} (kHz)	1.180 5 (32)	1.172	Δ_{JK} (kHz)	−0.136 8 (33)	−0.145
D_K (kHz)	[−0.311]	−0.311	Δ_K (kHz)	[0.786]	0.786
d_1 (kHz)	[−0.249]	−0.249	δ_J (kHz)	[0.249]	0.249
d_2 (kHz)	[−0.110]	−0.110	δ_K (kHz)	[0.511]	0.511
H_J (Hz)	[−0.000 188]	−0.000 188	Φ_J (Hz)	[0.000 201]	0.000 201
H_{JK} (Hz)	[0.002 01]	0.002 01	Φ_{JK} (Hz)	[0.000 327]	0.000 327
H_{KJ} (Hz)	[−0.001 82]	−0.001 82	Φ_{KJ} (Hz)	[−0.002 05]	−0.002 05
H_K (Hz)	[0.000 578]	0.000 578	Φ_K (Hz)	[0.002 10]	0.002 10
h_1 (Hz)	[0.000 065 2]	0.000 065 2	ϕ_J (Hz)	[0.000 100]	0.000 100
h_2 (Hz)	[0.000 194]	0.000 194	ϕ_{JK} (Hz)	[0.000 349]	0.000 349
h_3 (Hz)	[0.000 035]	0.000 035	ϕ_K (Hz)	[0.000 712]	0.000 712
N_{lines} [b]	91		N_{lines} [b]	91	
σ_{fit} (MHz)	0.044		σ_{fit} (MHz)	0.044	

[a] Evaluated using the cc-pCVTZ basis set. [b] Number of independent transitions.

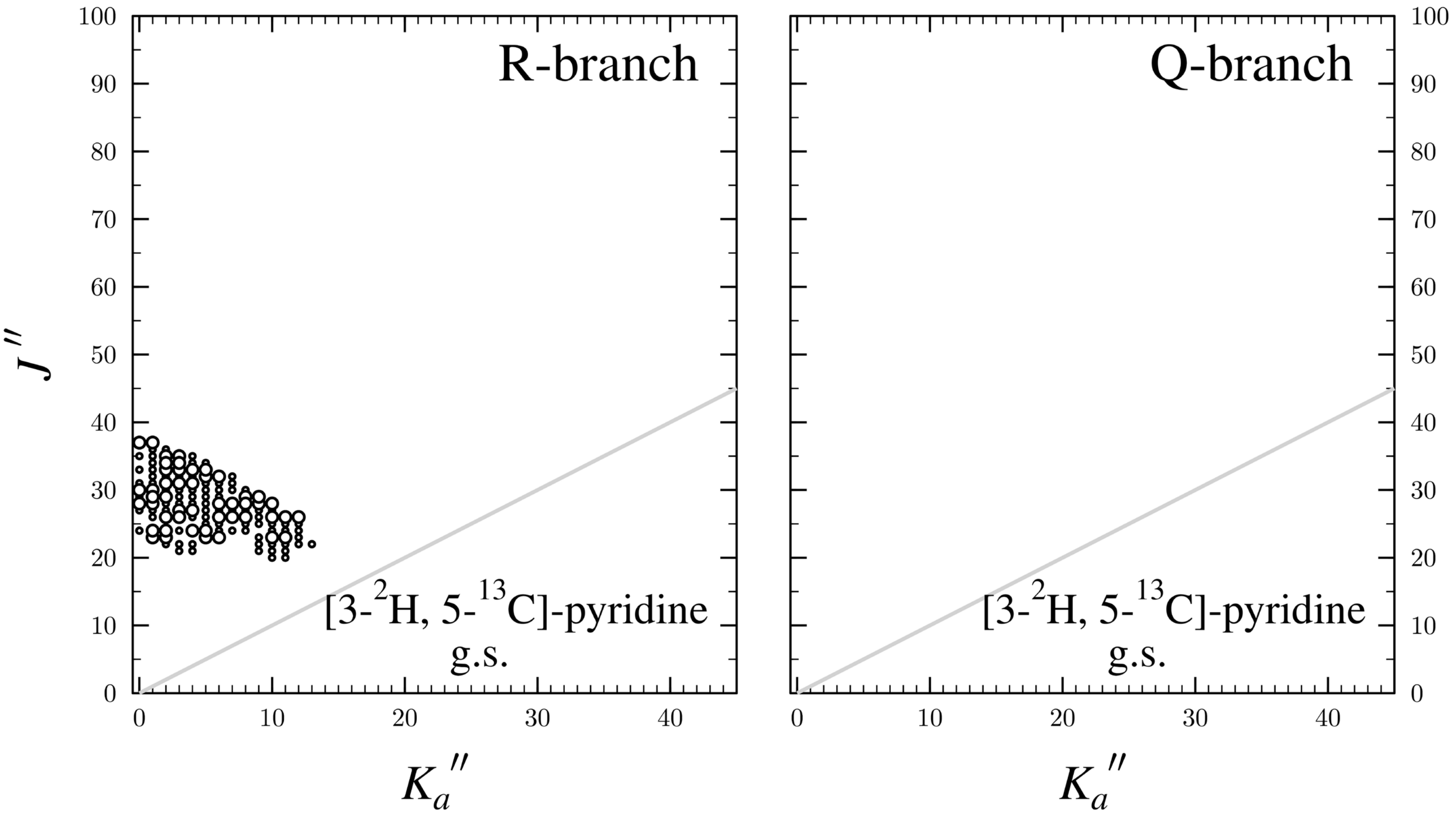

Figure S15. Data distribution plot for the least-squares fit of millimeter-wave spectroscopic data for [3-^{2}H, 5-^{13}C]-pyridine, ground vibrational state. Black (all) circles are measurements from the current work. The size of the outlined circle is proportional to the value of $|(f_{obs.} - f_{calc.})/\delta f|$, where δf is the frequency measurement uncertainty (50 kHz), and no quotient values are larger than three.

Table S16. Experimental and computed spectroscopic constants for [3-^{2}H, 6-^{13}C]-pyridine

	S Reduction, I^r representation			A Reduction, I^r representation	
	Experimental	CCSD(T) [a]		Experimental	CCSD(T) [a]
A_0 (MHz)	5883.190 (32)	5855	A_0 (MHz)	5883.185 (32)	5855
B_0 (MHz)	5449.683 (27)	5426	B_0 (MHz)	5449.688 (27)	5426
C_0 (MHz)	2828.485 47 (30)	2815	C_0 (MHz)	2828.484 87 (30)	2815
D_J (kHz)	0.383 441 (85)	0.381	Δ_J (kHz)	0.587 910 (85)	0.585
D_{JK} (kHz)	1.111 3 (23)	1.12	Δ_{JK} (kHz)	−0.114 8 (23)	−0.107
D_K (kHz)	−0.160 (12)	−0.236	Δ_K (kHz)	0.860 (12)	0.786
d_1 (kHz)	[−0.243]	−0.243	δ_J (kHz)	[0.243]	0.243
d_2 (kHz)	[−0.102]	−0.102	δ_K (kHz)	[0.544]	0.544
H_J (Hz)	[−0.000 137]	−0.000 137	Φ_J (Hz)	[0.000 203]	0.000 203
H_{JK} (Hz)	[0.001 59]	0.001 59	Φ_{JK} (Hz)	[0.000 296]	0.000 296
H_{KJ} (Hz)	[−0.001 15]	−0.001 15	Φ_{KJ} (Hz)	[−0.001 93]	−0.001 93
H_K (Hz)	[0.000 261]	0.000 261	Φ_K (Hz)	[0.002 00]	0.002 00
h_1 (Hz)	[0.000 066 3]	0.000 066 3	ϕ_J (Hz)	[0.000 101]	0.000 101
h_2 (Hz)	[0.000 170]	0.000 170	ϕ_{JK} (Hz)	[0.000 344]	0.000 344
h_3 (Hz)	[0.000 034 9]	0.000 034 9	ϕ_K (Hz)	[0.000 856]	0.000 856
N_{lines} [b]	98		N_{lines} [b]	98	
σ_{fit} (MHz)	0.042		σ_{fit} (MHz)	0.042	

[a] Evaluated using the cc-pCVTZ basis set. [b] Number of independent transitions.

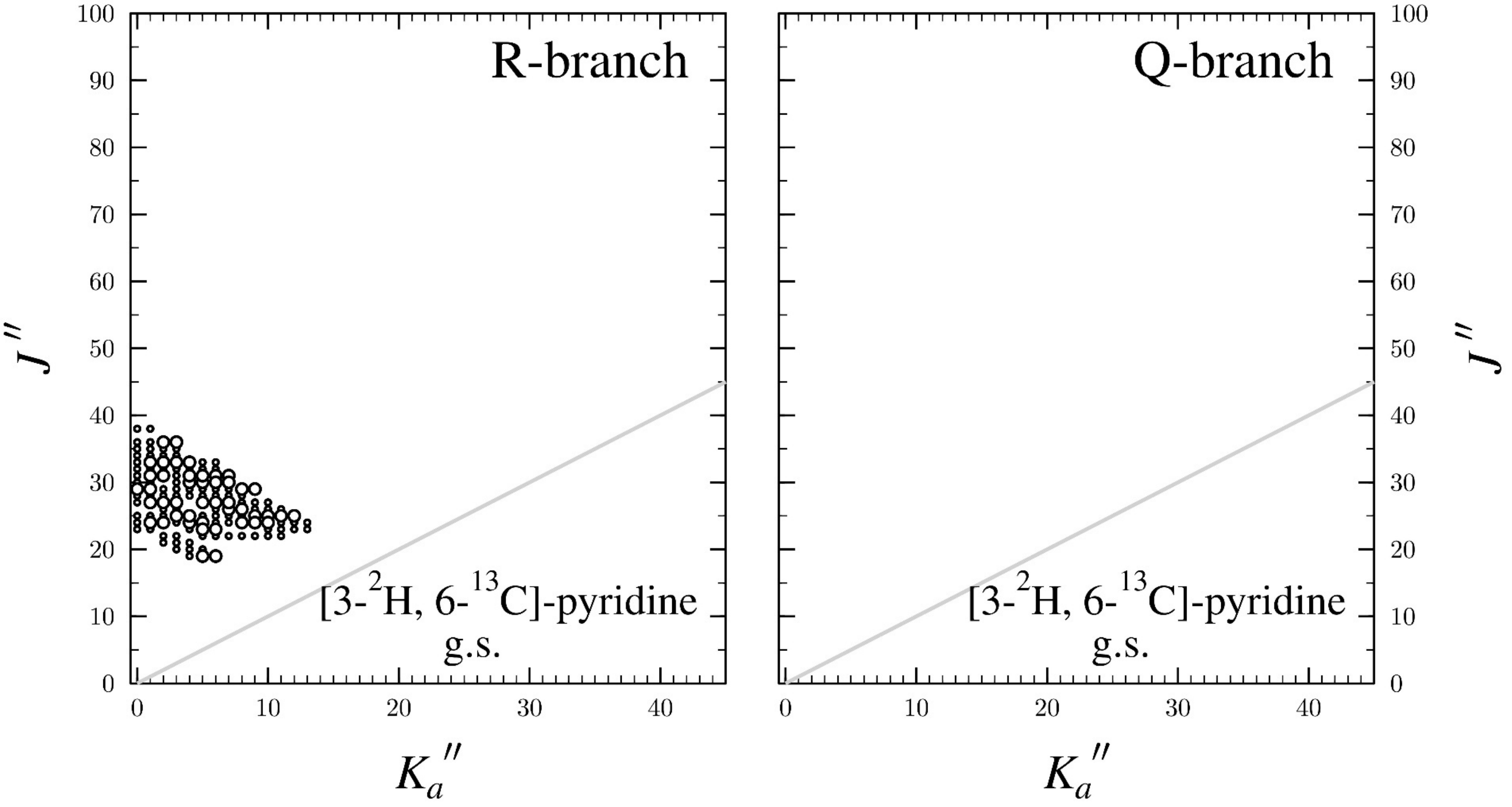

Figure S16. Data distribution plot for the least-squares fit of millimeter-wave spectroscopic data for [3-^{2}H, 6-^{13}C]-pyridine, ground vibrational state. Black (all) circles are measurements from the current work. The size of the outlined circle is proportional to the value of $|(f_{obs.} - f_{calc.})/\delta f|$, where δf is the frequency measurement uncertainty (50 kHz), and no quotient values are larger than three.

Table S17. Experimental and Computed Spectroscopic Constants for [2,3,4,5,6-^{2}H]-Pyridine

	S Reduction, I^r representation			A Reduction, I^r representation	
	Experimental [a]	CCSD(T) [b]		Experimental [a]	CCSD(T) [b]
A_0 (MHz)	5080.252 536 (75)	5052	A_0 (MHz)	5080.251 751 (80)	5052
B_0 (MHz)	4979.016 200 (66)	4963	B_0 (MHz)	4979.017 174 (70)	4963
C_0 (MHz)	2514.234 472 (49)	2503	C_0 (MHz)	2514.234 133 (49)	2503
D_J (kHz)	0.288 726 (26)	0.287	Δ_J (kHz)	0.434 773 (29)	0.431
D_{JK} (kHz)	0.694 792 (90)	0.675	Δ_{JK} (kHz)	−0.181 59 (10)	−0.189
D_K (kHz)	−0.054 455 (75)	−0.050 1	Δ_K (kHz)	0.675 948 (66)	0.670
d_1 (kHz)	−0.180 270 (12)	−0.179	δ_J (kHz)	0.180 303 (14)	0.179
d_2 (kHz)	−0.072 990 4 (32)	−0.072 0	δ_K (kHz)	0.315 792 (19)	0.308
H_J (Hz)	−0.000 087 6 (46)	−0.000 036 6	Φ_J (Hz)	0.000 112 6 (57)	0.000 135
H_{JK} (Hz)	0.000 777 (15)	0.000 410	Φ_{JK} (Hz)	−0.000 108 (19)	−0.000 202
H_{KJ} (Hz)	−0.000 132 9 (87)	0.000 305	Φ_{KJ} (Hz)	−0.000 363 (51)	−0.000 226
H_K (Hz)	−0.000 284 3 (71)	−0.000 354	Φ_K (Hz)	0.000 704 (71)	0.000 617
h_1 (Hz)	0.000 025 3 (22)	0.000 042 9	ϕ_J (Hz)	0.000 064 0 (28)	0.000 067 2
h_2 (Hz)	[0.000 085 7]	0.000 085 7	ϕ_{JK} (Hz)	[0.000 034 8]	0.000 034 8
h_3 (Hz)	[0.000 024 3]	0.000 024 3	ϕ_K (Hz)	0.000 553 (25)	0.000 549 8
N_{lines} [c]	3622		N_{lines} [e]	3622	
σ_{fit} (MHz)	0.038		σ_{fit} (MHz)	0.038	

[a] Includes transitions from Spycher *et al.*[74] [b] Evaluated using the cc-pCVTZ basis set. [c] Number of independent transitions.

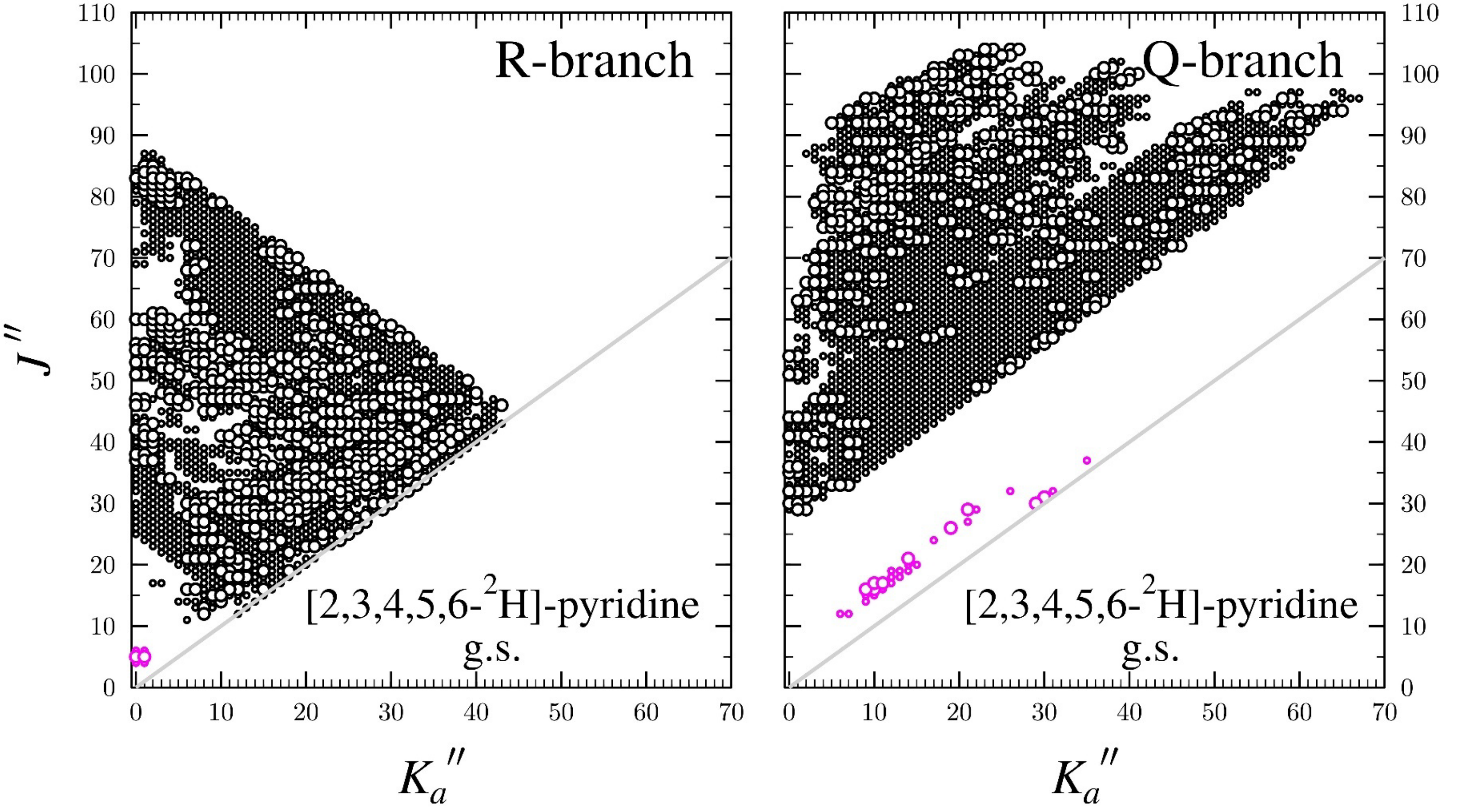

Figure S17. Data distribution plot for the least-squares fit of millimeter-wave spectroscopic data for [2,3,4,5,6-^{2}H]-pyridine, ground vibrational state. Black circles are measurements from the current work, while maroon circles are from previous work.[74] The size of the outlined circle is proportional to the value of $|(f_{\text{obs.}} - f_{\text{calc.}})/\delta f|$, where δf is the frequency measurement uncertainty (50 kHz), and no quotient values are larger than three.

Table S18. Experimental and Computed Spectroscopic Constants for [2,3,4,5,6-^{2}H, ^{15}N]-Pyridine

	S Reduction, I^r representation			A Reduction, I^r representation	
	Experimental	CCSD(T) [a]		Experimental	CCSD(T) [a]
A_0 (MHz)	4979.104 (30)	4963.1	A_0 (MHz)	4979.106 (30)	4963.1
B_0 (MHz)	4980.743 (30)	4952.4	B_0 (MHz)	4980.740 (30)	4952.4
C_0 (MHz)	2489.634 27 (15)	2478.4	C_0 (MHz)	2489.634 07 (15)	2478.4
D_J (kHz)	0.336 497 (93)	0.337	Δ_J (kHz)	0.481 604 (22)	0.480
D_{JK} (kHz)	0.511 28 (75)	0.488	Δ_{JK} (kHz)	−0.352 05 (47)	−0.367
D_K (kHz)	[−0.036 1]	−0.036 1	Δ_K (kHz)	[0.677]	0.677
d_1 (kHz)	[−0.204]	−0.204	δ_J (kHz)	[0.204]	0.204
d_2 (kHz)	−0.072 545 (49)	−0.071 3	δ_K (kHz)	0.293 20 (34)	0.288
H_J (Hz)	[−0.000 006 4]	−0.000 006 4	Φ_J (Hz)	[0.000 159]	0.000 159
H_{JK} (Hz)	[0.000 655]	0.000 655	Φ_{JK} (Hz)	[−0.000 092 5]	−0.000 092 5
H_{KJ} (Hz)	[−0.000 877]	−0.000 877	Φ_{KJ} (Hz)	[−0.000 870]	−0.000 870
H_K (Hz)	[0.000 498]	0.000 498	Φ_K (Hz)	[0.001 07]	0.001 07
h_1 (Hz)	[0.000 064 2]	0.000 064 2	ϕ_J (Hz)	[0.000 079 4]	0.000 079 4
h_2 (Hz)	[0.000 082 8]	0.000 082 8	ϕ_{JK} (Hz)	[0.000 099 6]	0.000 099 6
h_3 (Hz)	[0.000 015 2]	0.000 015 2	ϕ_K (Hz)	[0.000 351]	0.000 351
N_{lines} [b]	163		N_{lines} [b]	163	
σ_{fit} (MHz)	0.046		σ_{fit} (MHz)	0.046	

[a] Evaluated using the cc-pCVTZ basis set. [b] Number of independent transitions.

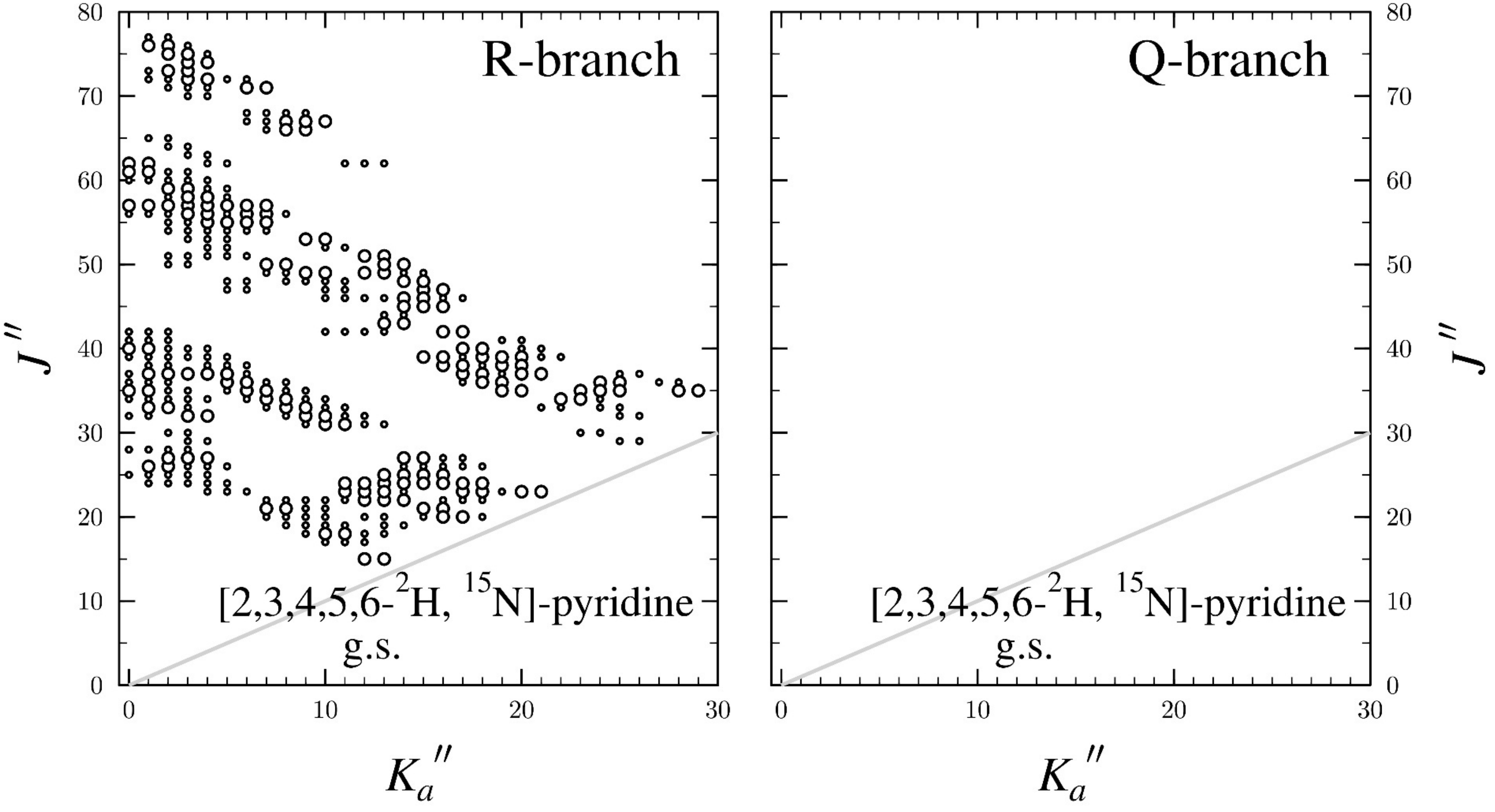

Figure S18. Data distribution plot for the least-squares fit of millimeter-wave spectroscopic data for [2,3,4,5,6-^{2}H, ^{15}N]-pyridine, ground vibrational state. Black (all) circles are measurements from the current work. The size of the outlined circle is proportional to the value of $|(f_{obs.} - f_{calc.})/\delta f|$, where δf is the frequency measurement uncertainty (50 kHz), and no quotient values are larger than three.

Table S19. Experimental and computed spectroscopic constants for [2,3,4,5,6-^{2}H , 2-^{13}C]-pyridine

	S Reduction, I^r representation			A Reduction, I^r representation	
	Experimental	CCSD(T) [a]		Experimental	CCSD(T) [a]
A_0 (MHz)	5065.454 22 (19)	5038	A_0 (MHz)	5065.453 54 (19)	5038
B_0 (MHz)	4905.949 29 (21)	4889	B_0 (MHz)	4905.950 24 (21)	4889
C_0 (MHz)	2491.885 226 (78)	2481	C_0 (MHz)	2491.884 880 (77)	2481
D_J (kHz)	0.284 39 (14)	0.283	Δ_J (kHz)	0.426 906 (97)	0.424
D_{JK} (kHz)	0.688 11 (81)	0.673	Δ_{JK} (kHz)	−0.167 22 (50)	−0.173
D_K (kHz)	−0.060 47 (74)	−0.062 2	Δ_K (kHz)	0.652 54 (47)	0.643
d_1 (kHz)	−0.176 895 (48)	−0.176	δ_J (kHz)	0.176 891 (48)	0.176
d_2 (kHz)	−0.071 262 (30)	−0.070 5	δ_K (kHz)	0.322 58 (13)	0.316
H_J (Hz)	−0.000 0461 (26)	−0.000 050 7	Φ_J (Hz)	0.000 126 4 (25)	0.000 122
H_{JK} (Hz)	0.000 856 (21)	0.000 682	Φ_{JK} (Hz)	[−0.000 018 7]	−0.000 018 7
H_{KJ} (Hz)	−0.000 800 (50)	−0.000 446	Φ_{KJ} (Hz)	−0.000 777 (81)	−0.000 702
H_K (Hz)	[0.000 144]	0.000 144	Φ_K (Hz)	0.000 968 (95)	0.000 929
h_1 (Hz)	[0.000 042 1]	0.000 042 1	ϕ_J (Hz)	[0.000 060 8]	0.000 060 8
h_2 (Hz)	[0.000 086 4]	0.000 086 4	ϕ_{JK} (Hz)	[0.000 109]	0.000 109
h_3 (Hz)	[0.000 018 7]	0.000 018 7	ϕ_K (Hz)	[0.000 440]	0.000 440
N_{lines} [b]	1208		N_{lines} [b]	1208	
σ_{fit} (MHz)	0.042		σ_{fit} (MHz)	0.042	

[a] Evaluated using the cc-pCVTZ basis set. [b] Number of independent transitions.

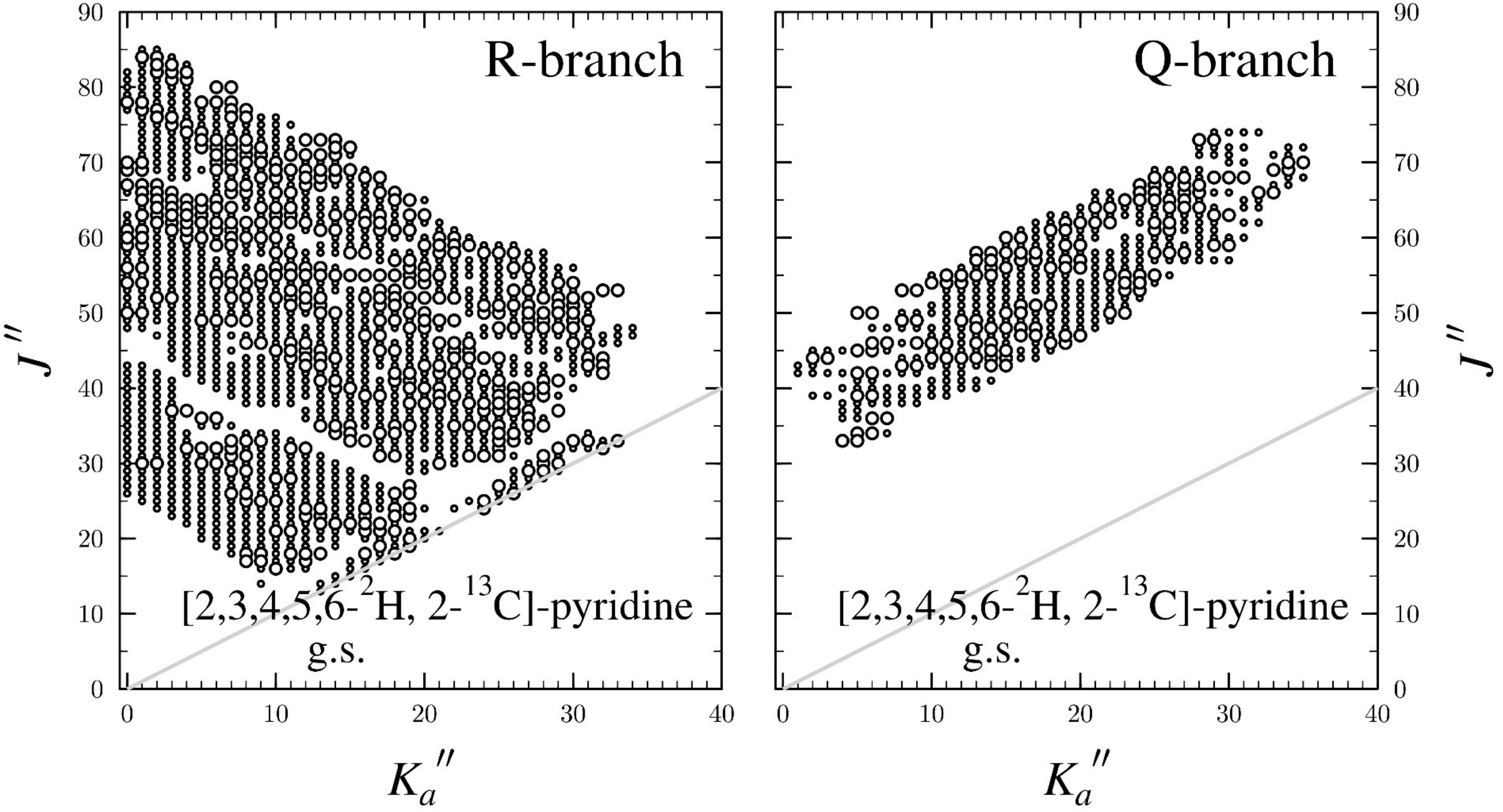

Figure S19. Data distribution plot for the least-squares fit of millimeter-wave spectroscopic data for [2,3,4,5,6-^{2}H, 2-^{13}C]-pyridine, ground vibrational state. Black (all) circles are measurements from the current work. The size of the outlined circle is proportional to the value of $|(f_{obs.} - f_{calc.})/\delta f|$, where δf is the frequency measurement uncertainty (50 kHz), and no quotient values are larger than three.

Table S20. Experimental and computed spectroscopic constants for [2,3,4,5,6-^{2}H , 3-^{13}C]-pyridine

	S Reduction, I^r representation			A Reduction, I^r representation	
	Experimental	CCSD(T) [a]		Experimental	CCSD(T) [a]
A_0 (MHz)	5067.756 31 (16)	5040	A_0 (MHz)	5067.755 74 (16)	5040
B_0 (MHz)	4900.961 41 (16)	4884	B_0 (MHz)	4900.962 42 (16)	4884
C_0 (MHz)	2491.154 682 (73)	2480	C_0 (MHz)	2491.154 439 (72)	2480
D_J (kHz)	0.286 80 (10)	0.284	Δ_J (kHz)	0.428 108 (63)	0.424
D_{JK} (kHz)	0.673 46 (63)	0.662	Δ_{JK} (kHz)	−0.174 46 (36)	−0.177
D_K (kHz)	−0.046 85 (59)	−0.050 1	Δ_K (kHz)	0.659 82 (36)	0.649
d_1 (kHz)	−0.177 506 (29)	−0.176	δ_J (kHz)	0.177 476 (29)	0.176
d_2 (kHz)	−0.070 690 (23)	−0.069 9	δ_K (kHz)	0.321 55 (11)	0.316
H_J (Hz)	−0.000 035 1 (25)	−0.000 045 3	Φ_J (Hz)	0.000 127 7 (24)	0.000 124
H_{JK} (Hz)	[0.000 627]	0.000 627	Φ_{JK} (Hz)	[−0.000 042]	−0.000 042
H_{KJ} (Hz)	[−0.000 344]	−0.000 344	Φ_{KJ} (Hz)	[−0.000 648]	−0.000 648
H_K (Hz)	[0.000 091 5]	0.000 091 5	Φ_K (Hz)	[0.000 895]	0.000 895
h_1 (Hz)	[0.000 042 5]	0.000 042 5	ϕ_J (Hz)	[0.000 061 6]	0.000 061 6
h_2 (Hz)	[0.000 084 5]	0.000 084 5	ϕ_{JK} (Hz)	[0.000 099 7]	0.000 099 7
h_3 (Hz)	[0.000 019 1]	0.000 019 1	ϕ_K (Hz)	[0.000 455]	0.000 455
N_{lines} [b]	1225		N_{lines} [b]	1225	
σ_{fit} (MHz)	0.040		σ_{fit} (MHz)	0.040	

[a] Evaluated using the cc-pCVTZ basis set. [b] Number of independent transitions.

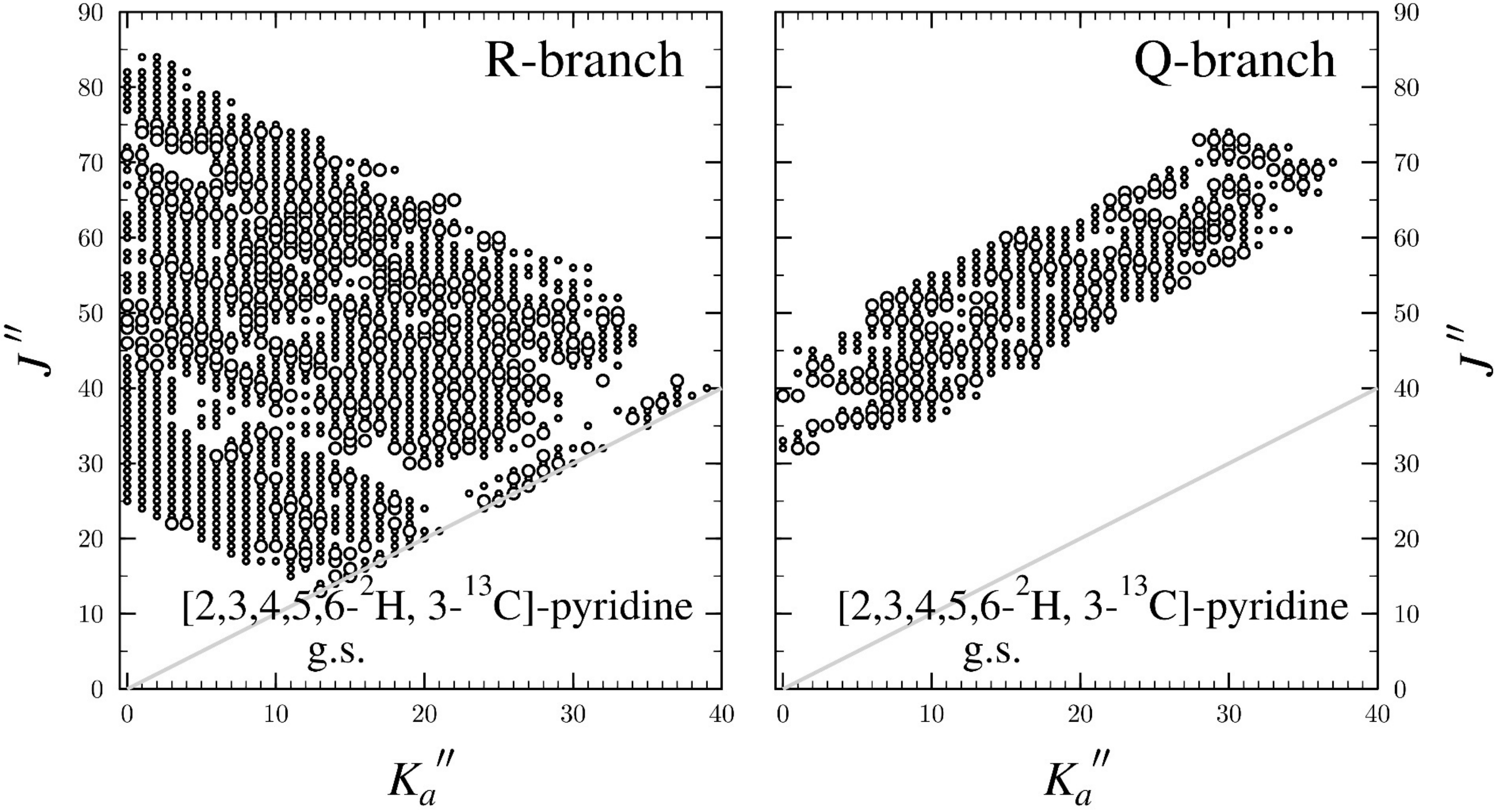

Figure S20. Data distribution plot for the least-squares fit of millimeter-wave spectroscopic data for [2,3,4,5,6-^{2}H, 3-^{13}C]-pyridine, ground vibrational state. Black (all) circles are measurements from the current work. The size of the outlined circle is proportional to the value of $|(f_{obs.} - f_{calc.})/\delta f|$, where δf is the frequency measurement uncertainty (50 kHz), and no quotient values are larger than three.

Table S21. Experimental and computed spectroscopic constants for [2,3,4,5,6-^{2}H , 4-^{13}C]-pyridine

	S Reduction, I^r representation			A Reduction, I^r representation	
	Experimental	CCSD(T) [a]		Experimental	CCSD(T) [a]
A_0 (MHz)	4985.145 (21)	4963	A_0 (MHz)	4985.168 (14)	4963
B_0 (MHz)	4979.141 (21)	4957	B_0 (MHz)	4979.119 (14)	4957
C_0 (MHz)	2490.741 902 (64)	2480	C_0 (MHz)	2490.741 709 (63)	2480
D_J (kHz)	0.351 0 (100)	0.338	Δ_J (kHz)	0.484 9 (39)	0.481
D_{JK} (kHz)	0.474 (30)	0.493	Δ_{JK} (kHz)	[−0.368]	−0.368
D_K (kHz)	[−0.040 9]	−0.040 9	Δ_K (kHz)	0.694 (20)	0.677
d_1 (kHz)	−0.211 8 (50)	−0.205	δ_J (kHz)	0.205 8 (20)	0.205
d_2 (kHz)	−0.072 826 5 (88)	−0.071 8	δ_K (kHz)	0.290 6 (60)	0.289
H_J (Hz)	[−0.000 008 5]	−0.000 008 5	Φ_J (Hz)	[0.000 160]	0.000 160
H_{JK} (Hz)	[0.000 677]	0.000 677	Φ_{JK} (Hz)	[−0.000 085 5]	−0.000 085 5
H_{KJ} (Hz)	[−0.000 913]	−0.000 913	Φ_{KJ} (Hz)	[−0.000 890]	−0.000 890
H_K (Hz)	[0.000 514]	0.000 514	Φ_K (Hz)	[0.001 08]	0.001 08
h_1 (Hz)	[0.000 064 3]	0.000 064 3	ϕ_J (Hz)	[0.000 079 5]	0.000 079 5
h_2 (Hz)	[0.000 084]	0.000 084	ϕ_{JK} (Hz)	[0.000 103]	0.000 103
h_3 (Hz)	[0.000 015 2]	0.000 015 2	ϕ_K (Hz)	[0.000 348]	0.000 348
N_{lines} [b]	545		N_{lines} [b]	545	
σ_{fit} (MHz)	0.043		σ_{fit} (MHz)	0.042	

[a] Evaluated using the cc-pCVTZ basis set. [b] Number of independent transitions.

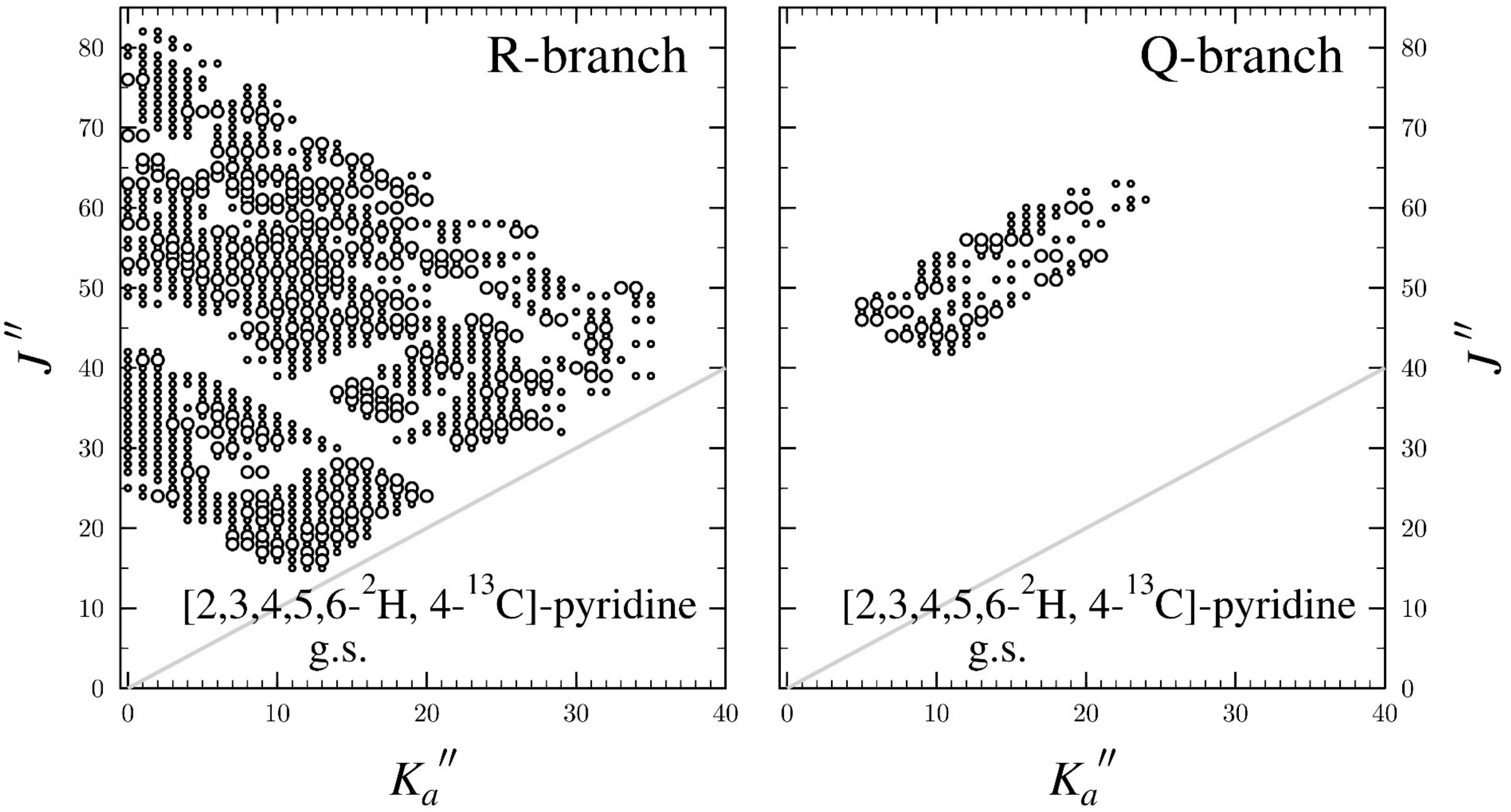

Figure S21. Data distribution plot for the least-squares fit of millimeter-wave spectroscopic data for [2,3,4,5,6-^{2}H, 4-^{13}C]-pyridine, ground vibrational state. Black (all) circles are measurements from the current work. The size of the outlined circle is proportional to the value of $|(f_{obs.} - f_{calc.})/\delta f|$, where δf is the frequency measurement uncertainty (50 kHz), and no quotient values are larger than three.

Table S22. Experimental and computed spectroscopic constants for [3,4,5,6-^{2}H]-pyridine

	S Reduction, I^r representation			A Reduction, I^r representation	
	Experimental	CCSD(T) [a]		Experimental	CCSD(T) [a]
A_0 (MHz)	5335.162 8 (25)	5312	A_0 (MHz)	5335.161 1 (25)	5312
B_0 (MHz)	5042.982 1 (24)	5020	B_0 (MHz)	5042.984 2 (24)	5020
C_0 (MHz)	2592.091 232 (67)	2580	C_0 (MHz)	2592.090 92 (13)	2580
D_J (kHz)	0.297 9 (19)	0.297	Δ_J (kHz)	0.465 9 (15)	0.462
D_{JK} (kHz)	0.923 (13)	0.905	Δ_{JK} (kHz)	−0.083 7 (74)	−0.088
D_K (kHz)	−0.246 (14)	−0.251	Δ_K (kHz)	0.592 4 (91)	0.577
d_1 (kHz)	−0.192 43 (78)	−0.192	δ_J (kHz)	0.192 62 (76)	0.192
d_2 (kHz)	−0.083 76 (53)	−0.082 8	δ_K (kHz)	0.414 7 (26)	0.410
H_J (Hz)	[−0.000 033 5]	−0.000 033 5	Φ_J (Hz)	0.000 143 7 (48)	0.000 136
H_{JK} (Hz)	[0.000 566]	0.000 566	Φ_{JK} (Hz)	[0.000 088 6]	0.000 088 6
H_{KJ} (Hz)	[0.000 054 4]	0.000 054 4	Φ_{KJ} (Hz)	[−0.000 897]	−0.000 897
H_K (Hz)	[−0.000 264]	−0.000 264	Φ_K (Hz)	[0.000 996]	0.000 996
h_1 (Hz)	[0.000 040 6]	0.000 040 6	ϕ_J (Hz)	[0.000 067 8]	0.000 067 8
h_2 (Hz)	[0.000 084 8]	0.000 084 8	ϕ_{JK} (Hz)	[0.000 175]	0.000 175
h_3 (Hz)	[0.000 027 2]	0.000 027 2	ϕ_K (Hz)	[0.000 597]	0.000 597
N_{lines} [b]	397		N_{lines} [b]	397	
σ_{fit} (MHz)	0.041		σ_{fit} (MHz)	0.041	

[a] Evaluated using the cc-pCVTZ basis set. [b] Number of independent transitions.

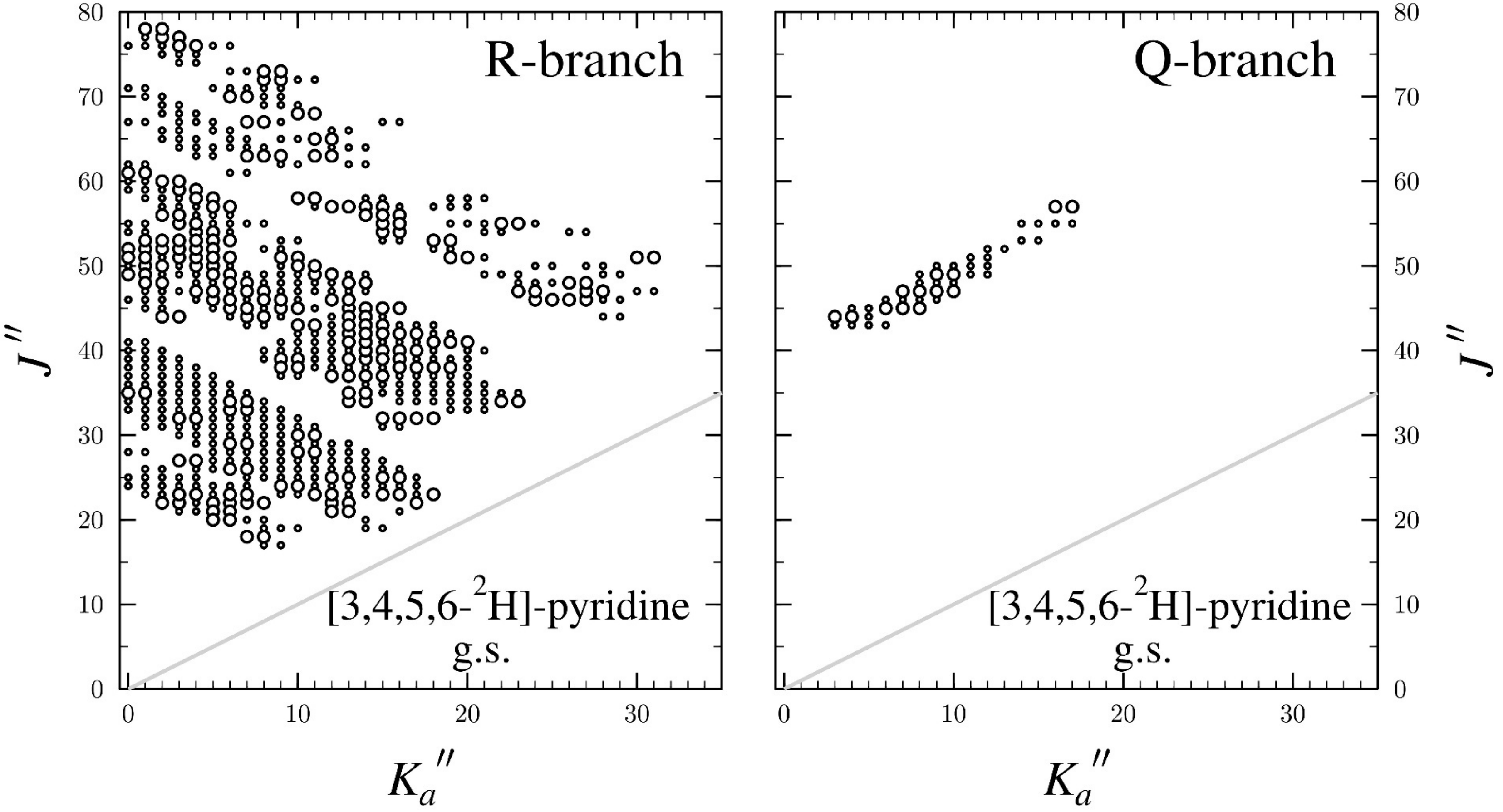

Figure S22. Data distribution plot for the least-squares fit of millimeter-wave spectroscopic data for [3,4,5,6-^{2}H]-pyridine, ground vibrational state. Black (all) circles are measurements from the current work. The size of the outlined circle is proportional to the value of $|(f_{obs.} - f_{calc.})/\delta f|$, where δf is the frequency measurement uncertainty (50 kHz), and no quotient values are larger than three.

Table S23. Experimental and computed spectroscopic constants for [2,4,5,6-^{2}H]-pyridine

	S Reduction, I^r representation			A Reduction, I^r representation	
	Experimental	CCSD(T) [a]		Experimental	CCSD(T) [a]
A_0 (MHz)	5332.937 40 (26)	5311	A_0 (MHz)	5332.936 56 (26)	5311
B_0 (MHz)	5049.508 00 (28)	5026	B_0 (MHz)	5049.509 15 (28)	5026
C_0 (MHz)	2593.296 665 (87)	2582	C_0 (MHz)	2593.296 175 (87)	2582
D_J (kHz)	0.306 50 (11)	0.302	Δ_J (kHz)	0.473 438 (82)	0.467
D_{JK} (kHz)	0.886 90 (62)	0.883	Δ_{JK} (kHz)	−0.114 70 (41)	−0.108
D_K (kHz)	−0.235 54 (50)	−0.242	Δ_K (kHz)	0.599 12 (31)	0.583
d_1 (kHz)	−0.196 541 (38)	−0.194	δ_J (kHz)	0.196 541 (38)	0.194
d_2 (kHz)	−0.083 467 (20)	−0.082 5	δ_K (kHz)	0.410 934 (98)	0.408
H_J (Hz)	−0.000 033 5 (24)	−0.000 025 8	Φ_J (Hz)	0.000 134 2 (24)	0.000 142
H_{JK} (Hz)	[0.000 453]	0.000 453	Φ_{JK} (Hz)	[0.000 016 3]	0.000 016 3
H_{KJ} (Hz)	[0.000 345]	0.000 345	Φ_{KJ} (Hz)	[−0.000 714]	−0.000 714
H_K (Hz)	[−0.000 459]	−0.000 459	Φ_K (Hz)	[0.000 869]	0.000 869
h_1 (Hz)	[0.000 041 8]	0.000 041 8	ϕ_J (Hz)	[0.000 070 6]	0.000 070 6
h_2 (Hz)	[0.000 083 8]	0.000 083 8	ϕ_{JK} (Hz)	[0.000 146]	0.000 146
h_3 (Hz)	[0.000 028 8]	0.000 028 8	ϕ_K (Hz)	[0.000 640]	0.000 640
N_{lines} [b]	848		N_{lines} [b]	848	
σ_{fit} (MHz)	0.043		σ_{fit} (MHz)	0.043	

[a] Evaluated using the cc-pCVTZ basis set. [b] Number of independent transitions.

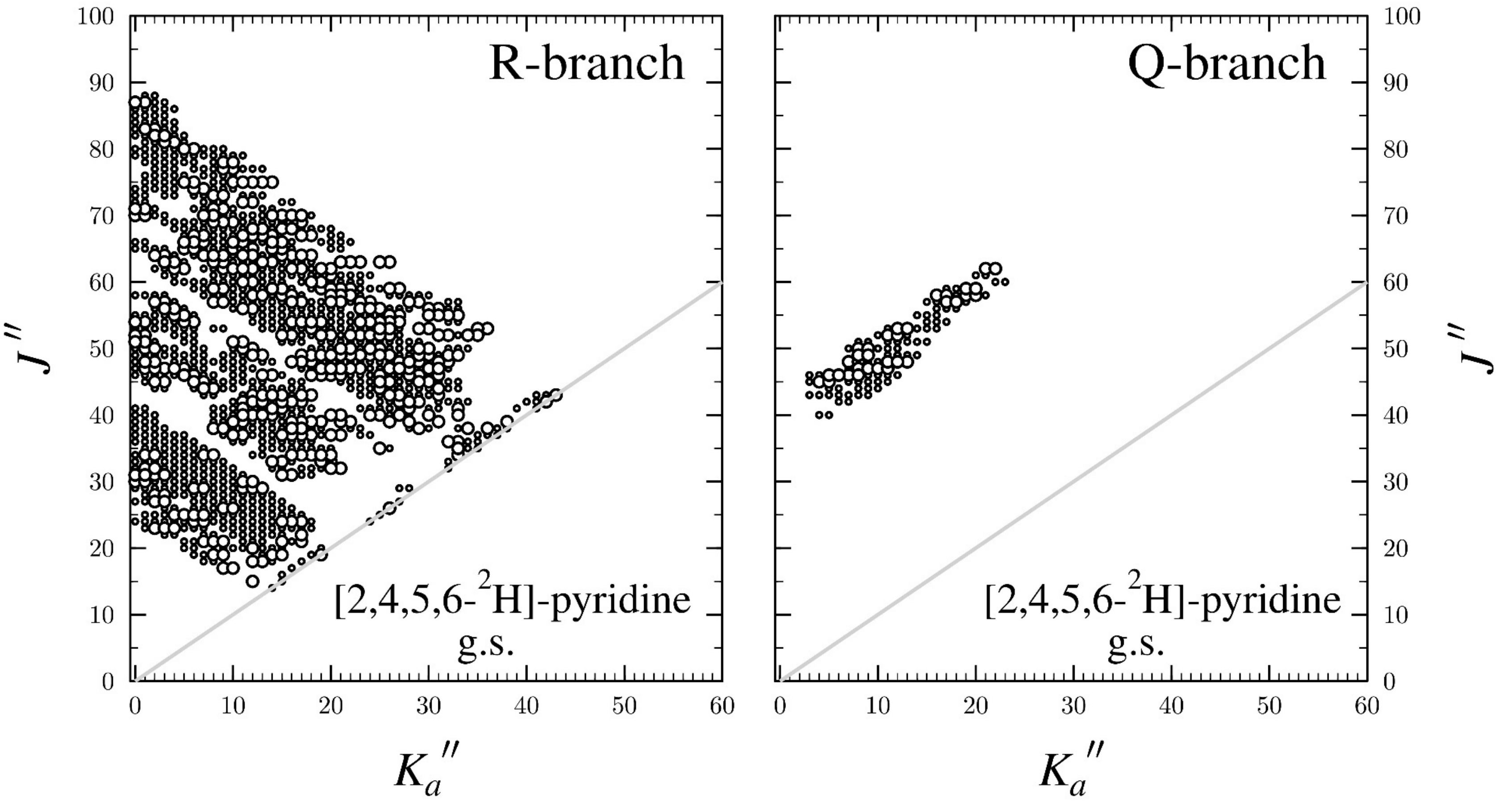

Figure S23. Data distribution plot for the least-squares fit of millimeter-wave spectroscopic data for [2,4,5,6-^{2}H]-pyridine, ground vibrational state. Black (all) circles are measurements from the current work. The size of the outlined circle is proportional to the value of $|(f_{obs.} - f_{calc.})/\delta f|$, where δf is the frequency measurement uncertainty (50 kHz), and no quotient values are larger than three.

Table S24. Experimental and computed spectroscopic constants for [2,3,5,6-^{2}H]-pyridine

	S Reduction, I^r representation			A Reduction, I^r representation	
	Experimental	CCSD(T) [a]		Experimental	CCSD(T) [a]
A_0 (MHz)	5417.681 65 (17)	5387	A_0 (MHz)	5417.680 90 (17)	5387
B_0 (MHz)	4979.133 43 (14)	4963	B_0 (MHz)	4979.134 55 (14)	4963
C_0 (MHz)	2594.199 953 (93)	2583	C_0 (MHz)	2594.199 447 (93)	2583
D_J (kHz)	0.289 157 (47)	0.287	Δ_J (kHz)	0.437 850 (40)	0.434
D_{JK} (kHz)	0.844 29 (23)	0.827	Δ_{JK} (kHz)	−0.047 86 (17)	−0.055 1
D_K (kHz)	−0.053 15 (18)	−0.054 7	Δ_K (kHz)	0.690 30 (12)	0.681
d_1 (kHz)	−0.178 858 (16)	−0.178	δ_J (kHz)	0.178 859 (17)	0.178
d_2 (kHz)	−0.074 347 0 (77)	−0.073 5	δ_K (kHz)	0.406 734 (42)	0.399
H_J (Hz)	−0.000 010 1 (18)	0.000 008 2	Φ_J (Hz)	0.000 116 3 (18)	0.000 135
H_{JK} (Hz)	[0.000 117]	0.000 117	Φ_{JK} (Hz)	[−0.000 089]	−0.000 089
H_{KJ} (Hz)	[0.000 760]	0.000 760	Φ_{KJ} (Hz)	[−0.000 455]	−0.000 455
H_K (Hz)	[−0.000 482]	−0.000 482	Φ_K (Hz)	[0.000 812]	0.000 812
h_1 (Hz)	[0.000 041 6]	0.000 041 6	ϕ_J (Hz)	[0.000 067 2]	0.000 067 2
h_2 (Hz)	[0.000 063 3]	0.000 063 3	ϕ_{JK} (Hz)	[0.000 100]	0.000 100
h_3 (Hz)	[0.000 025 6]	0.000 025 6	ϕ_K (Hz)	[0.000 716]	0.000 716
N_{lines} [b]	947		N_{lines} [b]	947	
σ_{fit} (MHz)	0.039		σ_{fit} (MHz)	0.040	

[a] Evaluated using the cc-pCVTZ basis set. [b] Number of independent transitions.

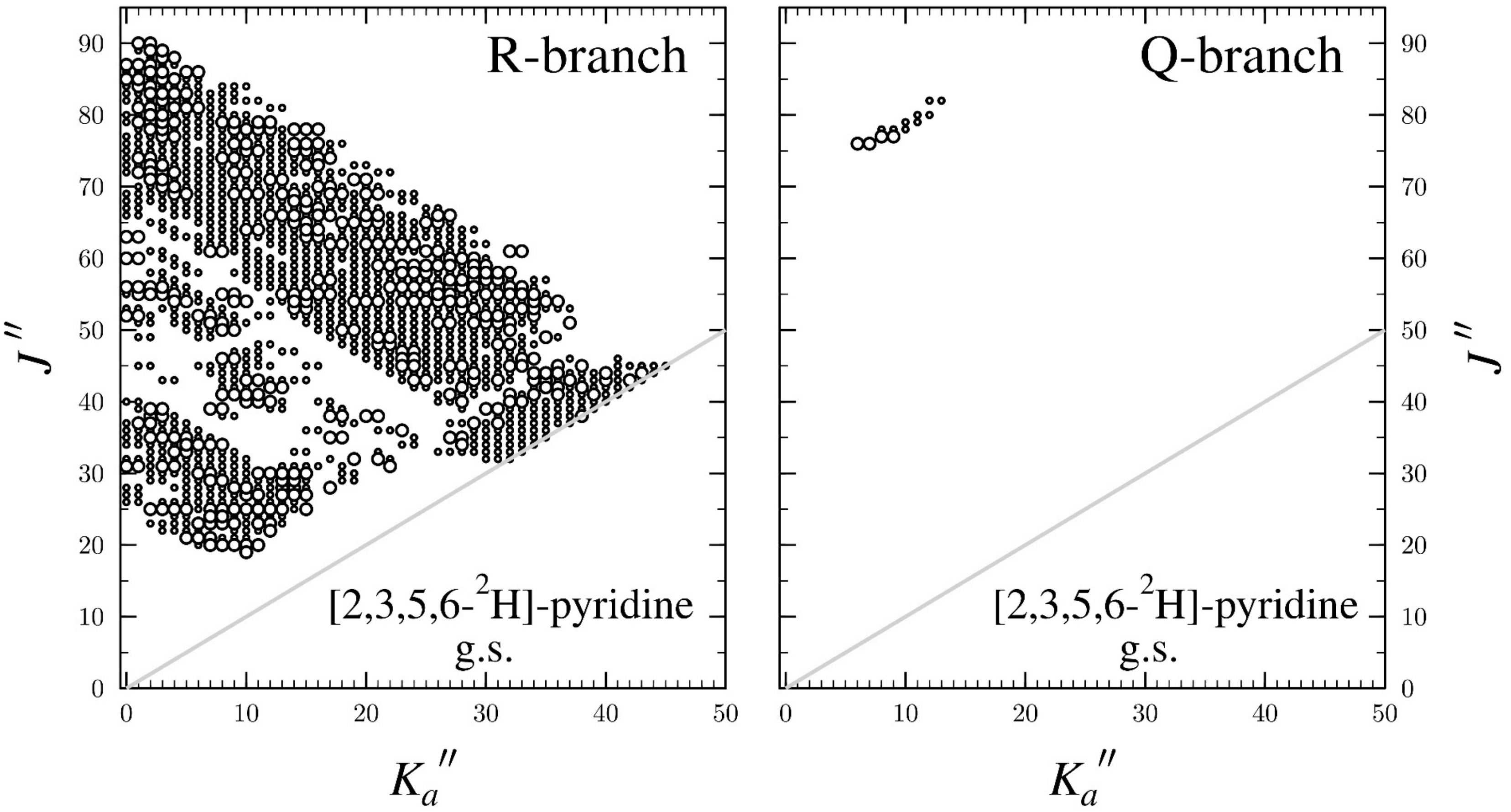

Figure S24. Data distribution plot for the least-squares fit of millimeter-wave spectroscopic data for [2,3,5,6-^{2}H]-pyridine, ground vibrational state. Black (all) circles are measurements from the current work. The size of the outlined circle is proportional to the value of $|(f_{obs.} - f_{calc.})/\delta f|$, where δf is the frequency measurement uncertainty (50 kHz), and no quotient values are larger than three.

Table S25. Experimental and computed spectroscopic constants for [2,5,6-^{2}H]-pyridine

	S Reduction, I^r representation			A Reduction, I^r representation	
	Experimental	CCSD(T) [a]		Experimental	CCSD(T) [a]
A_0 (MHz)	5574.901 82 (18)	5545	A_0 (MHz)	5574.899 91 (22)	5545
B_0 (MHz)	5160.809 20 (15)	5142	B_0 (MHz)	5160.810 05 (15)	5142
C_0 (MHz)	2679.498 04 (17)	2667	C_0 (MHz)	2679.497 12 (17)	2667
D_J (kHz)	0.343 098 (43)	0.340	Δ_J (kHz)	0.498 441 (45)	0.494
D_{JK} (kHz)	0.759 18 (12)	0.743	Δ_{JK} (kHz)	−0.173 433 (95)	−0.179
D_K (kHz)	0.072 10 (11)	0.070 0	Δ_K (kHz)	0.848 05 (17)	0.837
d_1 (kHz)	−0.205 720 (11)	−0.204	δ_J (kHz)	0.205 729 (11)	0.204
d_2 (kHz)	−0.077 704 5 (59)	−0.076 7	δ_K (kHz)	0.414 560 (31)	0.407
H_J (Hz)	−0.000 028 1 (24)	−0.000 020 3	Φ_J (Hz)	0.000 138 6 (24)	0.000 150
H_{JK} (Hz)	0.000 802 (13)	0.000 782	Φ_{JK} (Hz)	0.000 129 (14)	0.000 172
H_{KJ} (Hz)	−0.001 063 (35)	−0.000 938	Φ_{KJ} (Hz)	−0.001 396 (41)	−0.001 46
H_K (Hz)	[0.000 693]	0.000 693	Φ_K (Hz)	0.001 089 (71)	0.001 66
h_1 (Hz)	[0.000 055 4]	0.000 055 4	ϕ_J (Hz)	[0.000 074 8]	0.000 074 8
h_2 (Hz)	[0.000 085 3]	0.000 085 3	ϕ_{JK} (Hz)	[0.000 231]	0.000 231
h_3 (Hz)	[0.000 019 5]	0.000 019 5	ϕ_K (Hz)	[0.000 593]	0.000 593
N_{lines} [b]	1097		N_{lines} [b]	1097	
σ_{fit} (MHz)	0.039		σ_{fit} (MHz)	0.038	

[a] Evaluated using the cc-pCVTZ basis set. [b] Number of independent transitions.

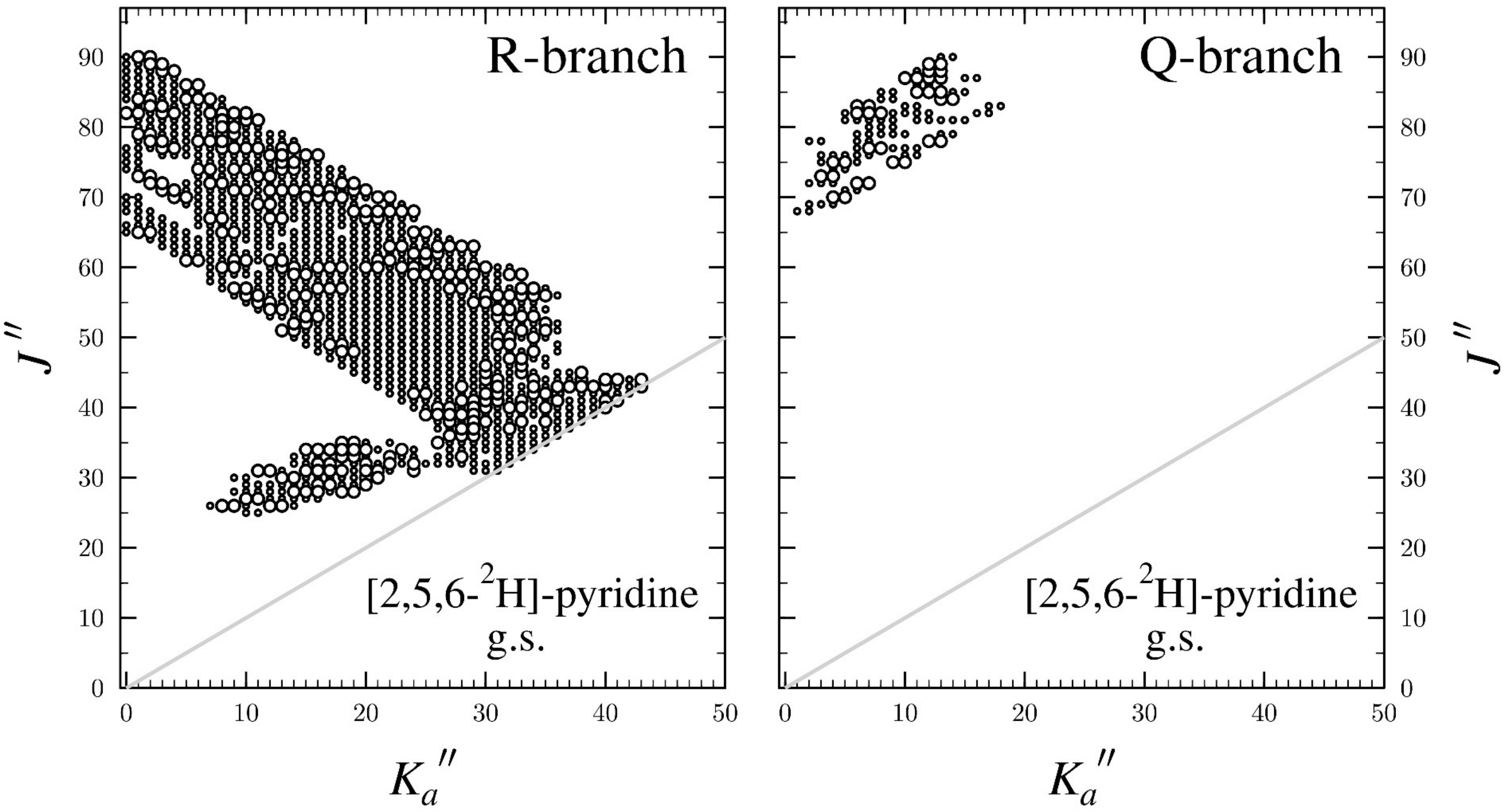

Figure S25. Data distribution plot for the least-squares fit of millimeter-wave spectroscopic data for [2,5,6-^{2}H]-pyridine, ground vibrational state. Black (all) circles are measurements from the current work. The size of the outlined circle is proportional to the value of $|(f_{obs.} - f_{calc.})/\delta f|$, where δf is the frequency measurement uncertainty (50 kHz), and no quotient values are larger than three.

Table S26. Experimental and computed spectroscopic constants for [3,4,5-^{2}H]-pyridine

	S Reduction, I^r representation			A Reduction, I^r representation	
	Experimental	CCSD(T) [a]		Experimental	CCSD(T) [a]
A_0 (MHz)	5434.142 35 (75)	5417	A_0 (MHz)	5434.141 57 (75)	5417
B_0 (MHz)	5266.588 52 (92)	5236	B_0 (MHz)	5266.589 55 (92)	5236
C_0 (MHz)	2674.069 83 (25)	2662	C_0 (MHz)	2674.069 39 (25)	2662
D_J (kHz)	0.418 48 (18)	0.411	Δ_J (kHz)	0.576 78 (25)	0.565
D_{JK} (kHz)	0.488 57 (65)	0.476	Δ_{JK} (kHz)	−0.461 22 (51)	−0.450
D_K (kHz)	0.126 3 (12)	0.139	Δ_K (kHz)	0.917 81 (77)	0.910
d_1 (kHz)	−0.244 59 (12)	−0.240	δ_J (kHz)	0.244 60 (13)	0.240
d_2 (kHz)	−0.079 148 (51)	−0.077 1	δ_K (kHz)	0.357 55 (23)	0.353
H_J (Hz)	[0.000 0320]	0.000 032 0	Φ_J (Hz)	[0.000 213]	0.000 213
H_{JK} (Hz)	[0.000 626]	0.000 626	Φ_{JK} (Hz)	[−0.000 124]	−0.000 124
H_{KJ} (Hz)	[−0.001 03]	−0.001 03	Φ_{KJ} (Hz)	[−0.001 24]	−0.001 24
H_K (Hz)	[0.000 795]	0.000 795	Φ_K (Hz)	[0.001 58]	0.001 58
h_1 (Hz)	[0.000 087 8]	0.000 0878	ϕ_J (Hz)	[0.000 106]	0.000 106
h_2 (Hz)	[0.000 090 4]	0.000 090 4	ϕ_{JK} (Hz)	[0.000 139]	0.000 139
h_3 (Hz)	[0.000 018 3]	0.000 018 3	ϕ_K (Hz)	[0.000 538]	0.000 538
N_{lines} [b]	317		N_{lines} [b]	317	
σ_{fit} (MHz)	0.041		σ_{fit} (MHz)	0.041	

[a] Evaluated using the cc-pCVTZ basis set. [b] Number of independent transitions.

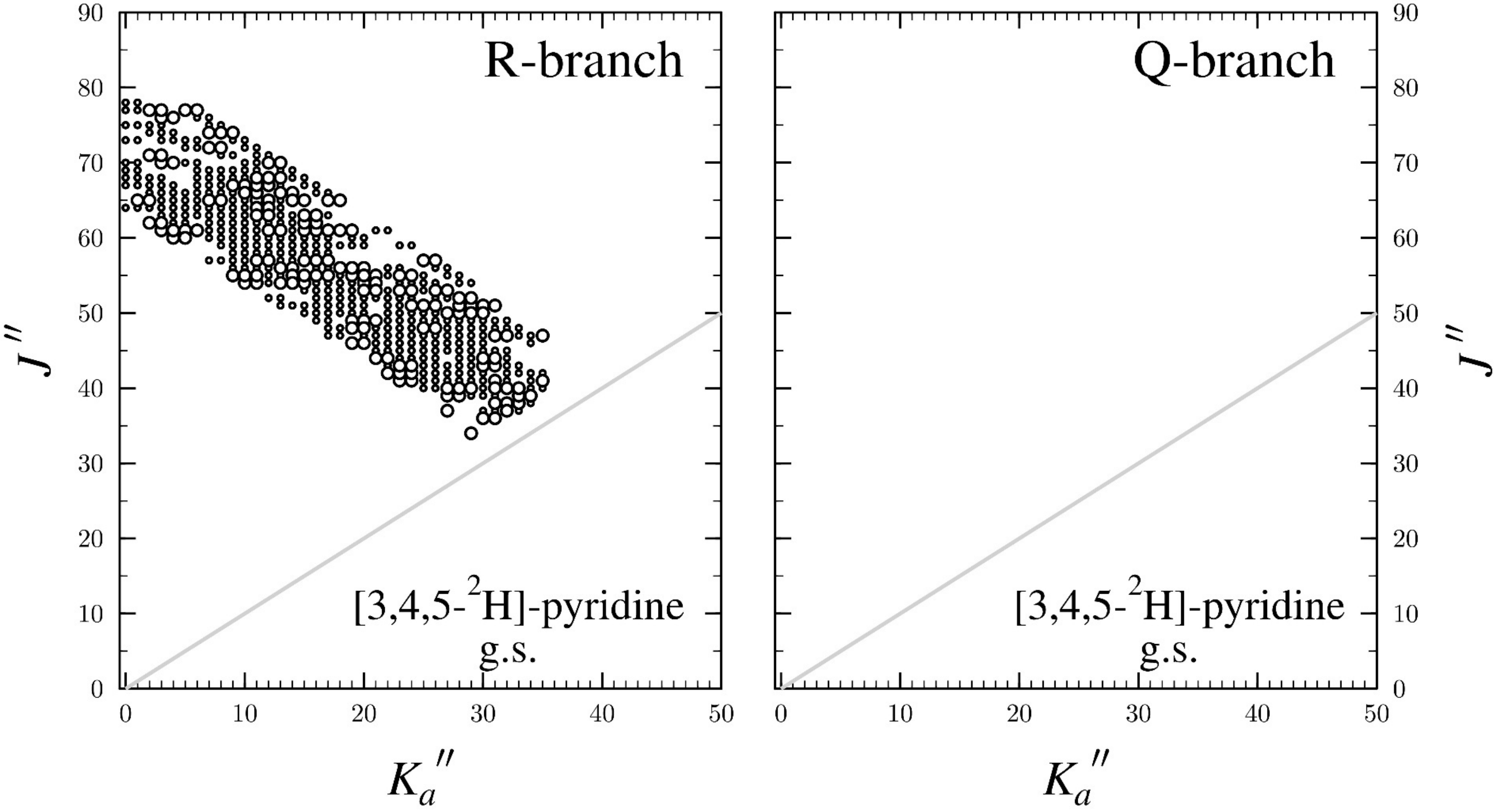

Figure S26. Data distribution plot for the least-squares fit of millimeter-wave spectroscopic data for [3,4,5-^{2}H]-pyridine, ground vibrational state. Black (all) circles are measurements from the current work. The size of the outlined circle is proportional to the value of $|(f_{obs.} - f_{calc.})/\delta f|$, where δf is the frequency measurement uncertainty (50 kHz), and no quotient values are larger than three.

Table S27. Experimental and computed spectroscopic constants for [2,4,6-^{2}H]-pyridine

	S Reduction, I^r representation			A Reduction, I^r representation	
	Experimental	CCSD(T) [a]		Experimental	CCSD(T) [a]
A_0 (MHz)	5482.212 (11)	5465	A_0 (MHz)	5482.209 (10)	5465
B_0 (MHz)	5230.970 (11)	5201	B_0 (MHz)	5230.973 (11)	5201
C_0 (MHz)	2676.385 31 (29)	2664	C_0 (MHz)	2676.385 01 (31)	2664
D_J (kHz)	0.384 7 (45)	0.398	Δ_J (kHz)	0.547 2 (34)	0.553
D_{JK} (kHz)	0.611 (14)	0.542	Δ_{JK} (kHz)	[−0.392]	−0.392
D_K (kHz)	[0.088 3]	0.088 3	Δ_K (kHz)	0.948 (19)	0.867
d_1 (kHz)	−0.228 1 (22)	−0.234	δ_J (kHz)	0.230 0 (17)	0.234
d_2 (kHz)	−0.079 309 (23)	−0.077 8	δ_K (kHz)	0.360 1 (55)	0.377
H_J (Hz)	[0.000 019 0]	0.000 0190	Φ_J (Hz)	[0.000 202]	0.000 202
H_{JK} (Hz)	[0.000 716]	0.000 716	Φ_{JK} (Hz)	[−0.000 027 5]	−0.000 027 5
H_{KJ} (Hz)	[−0.001 14]	−0.001 14	Φ_{KJ} (Hz)	[−0.001 41]	−0.001 41
H_K (Hz)	[0.000 835]	0.000 835	Φ_K (Hz)	[0.001 66]	0.001 66
h_1 (Hz)	[0.000 082 4]	0.000 082 4	ϕ_J (Hz)	[0.000 101]	0.000 101
h_2 (Hz)	[0.000 091 6]	0.000 091 6	ϕ_{JK} (Hz)	[0.000 178]	0.000 178
h_3 (Hz)	[0.000 018 3]	0.000 018 3	ϕ_K (Hz)	[0.000 535]	0.000 535
N_{lines} [b]	277		N_{lines} [b]	277	
σ_{fit} (MHz)	0.049		σ_{fit} (MHz)	0.049	

[a] Evaluated using the cc-pCVTZ basis set. [b] Number of independent transitions.

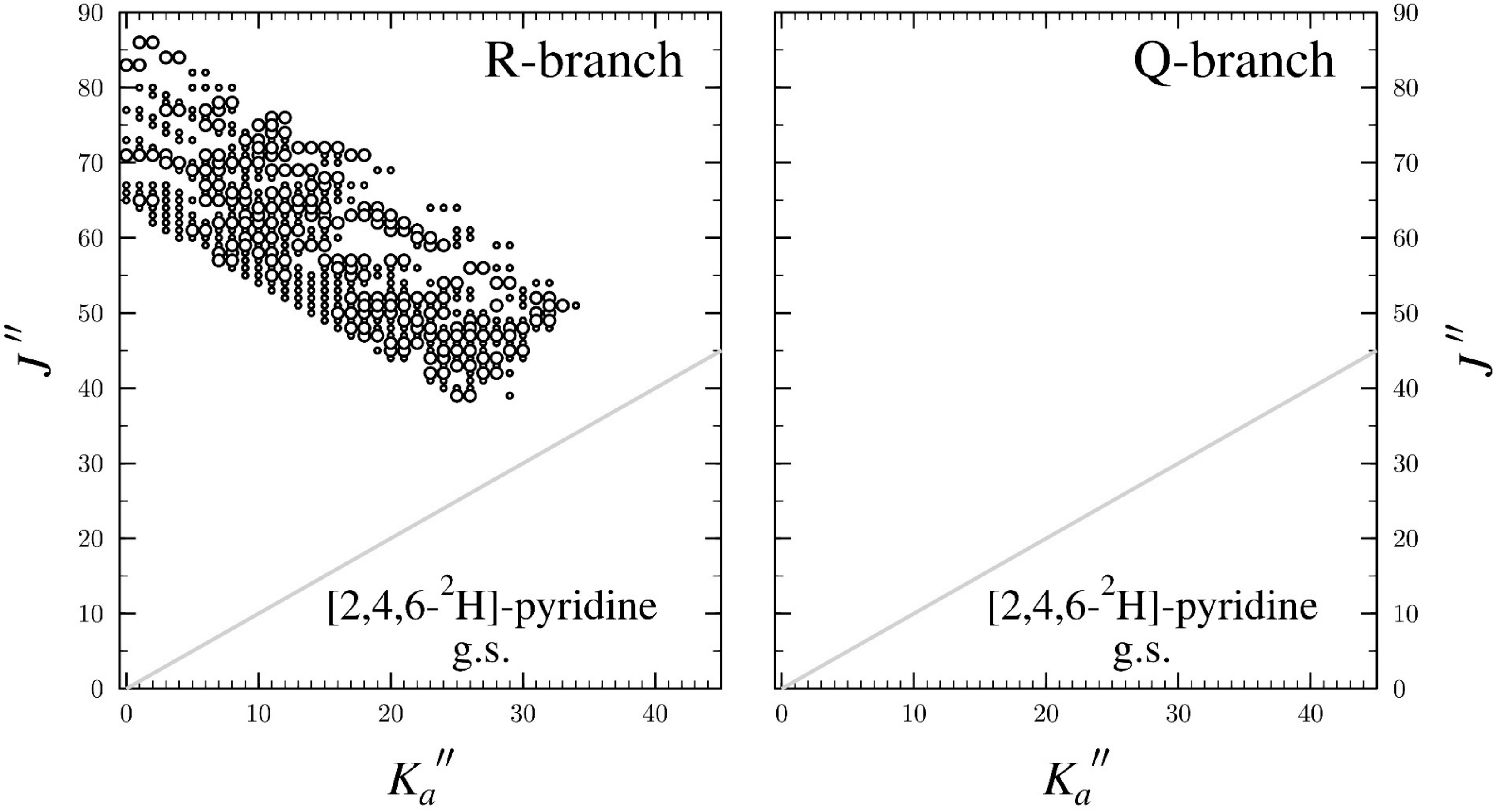

Figure S27. Data distribution plot for the least-squares fit of millimeter-wave spectroscopic data for [2,4,6-^{2}H]-pyridine, ground vibrational state. Black (all) circles are measurements from the current work. The size of the outlined circle is proportional to the value of $|(f_{obs.} - f_{calc.})/\delta f|$, where δf is the frequency measurement uncertainty (50 kHz), and no quotient values are larger than three.

Table S28. Experimental and computed spectroscopic constants for [2,3-^{2}H]-pyridine

	S Reduction, I^r representation			A Reduction, I^r representation	
	Experimental	CCSD(T) [a]		Experimental	CCSD(T) [a]
A_0 (MHz)	5604.564 (12)	5572	A_0 (MHz)	5604.571 (13)	5572
B_0 (MHz)	5471.256 (14)	5454	B_0 (MHz)	5471.257 (14)	5454
C_0 (MHz)	2768.048 03 (48)	2755	C_0 (MHz)	2768.046 87 (58)	2755
D_J (kHz)	0.376 88 (20)	0.374	Δ_J (kHz)	0.564 54 (22)	0.562
D_{JK} (kHz)	0.880 0 (16)	0.872	Δ_{JK} (kHz)	−0.257 1 (16)	−0.255
D_K (kHz)	[−0.042 5]	−0.042 5	Δ_K (kHz)	0.926 8 (66)	0.897
d_1 (kHz)	[−0.235]	−0.235	δ_J (kHz)	[0.235]	0.235
d_2 (kHz)	[−0.093 9]	−0.093 9	δ_K (kHz)	[0.408]	0.408
H_J (Hz)	[0.000 016 4]	0.000 016 4	Φ_J (Hz)	[0.000 217]	0.000 217
H_{JK} (Hz)	[0.000 117]	0.000 117	Φ_{JK} (Hz)	[−0.000 386]	−0.000 386
H_{KJ} (Hz)	[0.001 14]	0.001 14	Φ_{KJ} (Hz)	[−0.000 189]	−0.000 189
H_K (Hz)	[−0.000 757]	−0.000 757	Φ_K (Hz)	[0.000 876]	0.000 876
h_1 (Hz)	[0.000 067 8]	0.000 067 8	ϕ_J (Hz)	[0.000 108]	0.000 108
h_2 (Hz)	[0.000 100]	0.000 100	ϕ_{JK} (Hz)	[0.000 028 7]	0.000 028 7
h_3 (Hz)	[0.000 040 3]	0.000 040 3	ϕ_K (Hz)	[0.000 931]	0.000 931
N_{lines} [b]	125		N_{lines} [b]	125	
σ_{fit} (MHz)	0.042		σ_{fit} (MHz)	0.042	

[a] Evaluated using the cc-pCVTZ basis set. [b] Number of independent transitions.

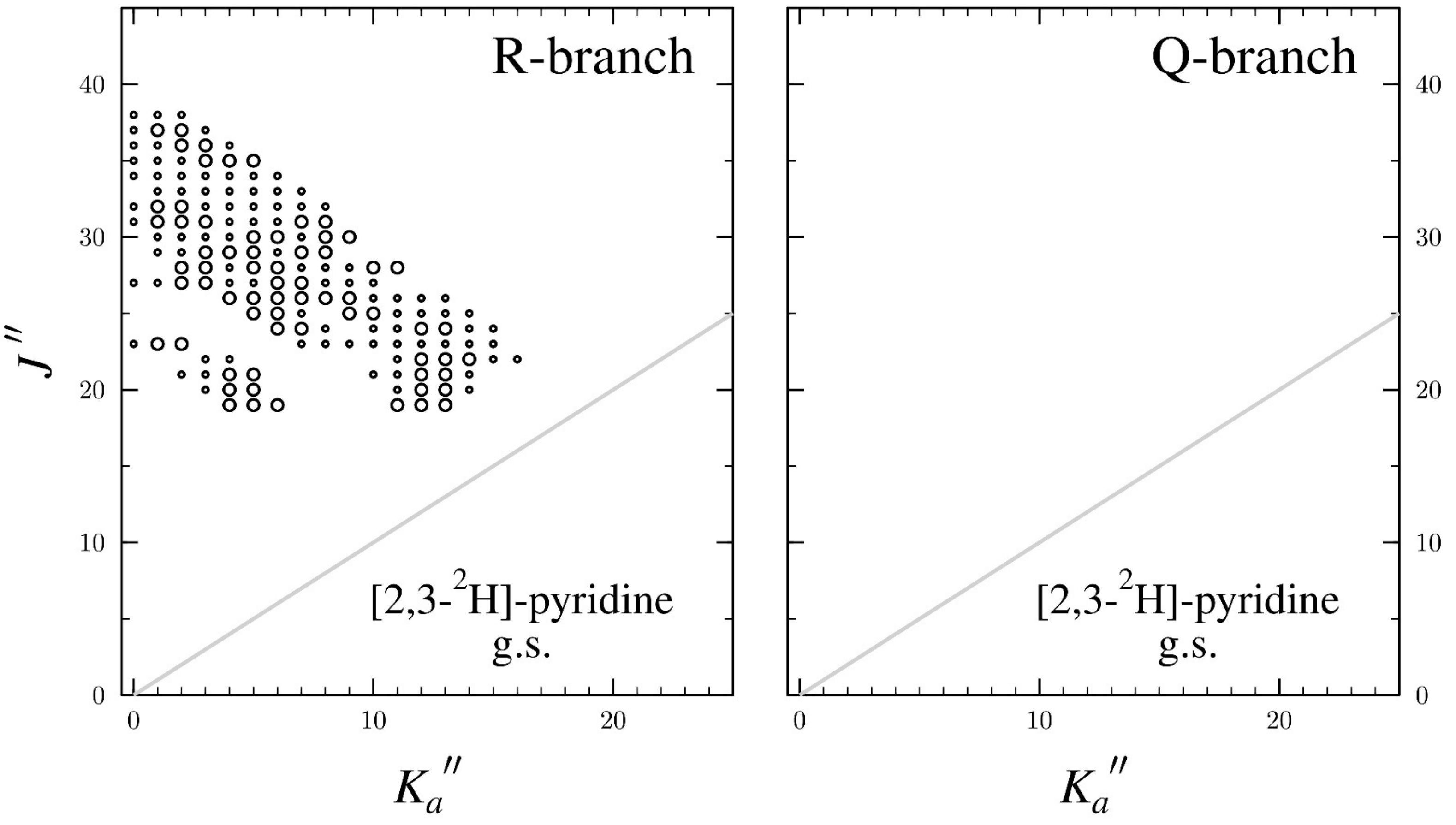

Figure S28. Data distribution plot for the least-squares fit of millimeter-wave spectroscopic data for [2,3-^{2}H]-pyridine, ground vibrational state. Black (all) circles are measurements from the current work. The size of the outlined circle is proportional to the value of $|(f_{obs.} - f_{calc.})/\delta f|$, where δf is the frequency measurement uncertainty (50 kHz), and no quotient values are larger than three.

Table S29. Experimental and computed spectroscopic constants for [2,5-^{2}H]-pyridine

	S Reduction, I^r representation			A Reduction, I^r representation	
	Experimental	CCSD(T) [a]		Experimental	CCSD(T) [a]
A_0 (MHz)	5876.912 1 (98)	5848	A_0 (MHz)	5876.924 (12)	5848
B_0 (MHz)	5222.506 (11)	5201	B_0 (MHz)	5222.504 (11)	5201
C_0 (MHz)	2764.709 74 (48)	2752	C_0 (MHz)	2764.708 53 (57)	2752
D_J (kHz)	0.360 12 (21)	0.357	Δ_J (kHz)	0.524 50 (22)	0.522
D_{JK} (kHz)	0.894 1 (22)	0.895	Δ_{JK} (kHz)	−0.095 6 (23)	−0.092 0
D_K (kHz)	[0.026 5]	0.026 5	Δ_K (kHz)	0.870 (10)	0.849
d_1 (kHz)	[−0.215]	−0.215	δ_J (kHz)	[0.215]	0.215
d_2 (kHz)	[−0.082 3]	−0.082 3	δ_K (kHz)	[0.503]	0.503
H_J (Hz)	[−0.000 027 9]	−0.000 027 9	Φ_J (Hz)	[0.000 163]	0.000 163
H_{JK} (Hz)	[0.000 922]	0.000 922	Φ_{JK} (Hz)	[0.000 339]	0.000 339
H_{KJ} (Hz)	[−0.001 03]	−0.001 03	Φ_{KJ} (Hz)	[−0.001 96]	−0.001 96
H_K (Hz)	[0.000 742]	0.000 742	Φ_K (Hz)	[0.002 06]	0.002 06
h_1 (Hz)	[0.000 058 3]	0.000 058 3	ϕ_J (Hz)	[0.000 081 3]	0.000 081 3
h_2 (Hz)	[0.000 095 6]	0.000 095 6	ϕ_{JK} (Hz)	[0.000 331]	0.000 331
h_3 (Hz)	[0.000 023 1]	0.000 023 1	ϕ_K (Hz)	[0.000 767]	0.000 767
N_{lines} [b]	111		N_{lines} [b]	111	
σ_{fit} (MHz)	0.040		σ_{fit} (MHz)	0.040	

[a] Evaluated using the cc-pCVTZ basis set. [b] Number of independent transitions.

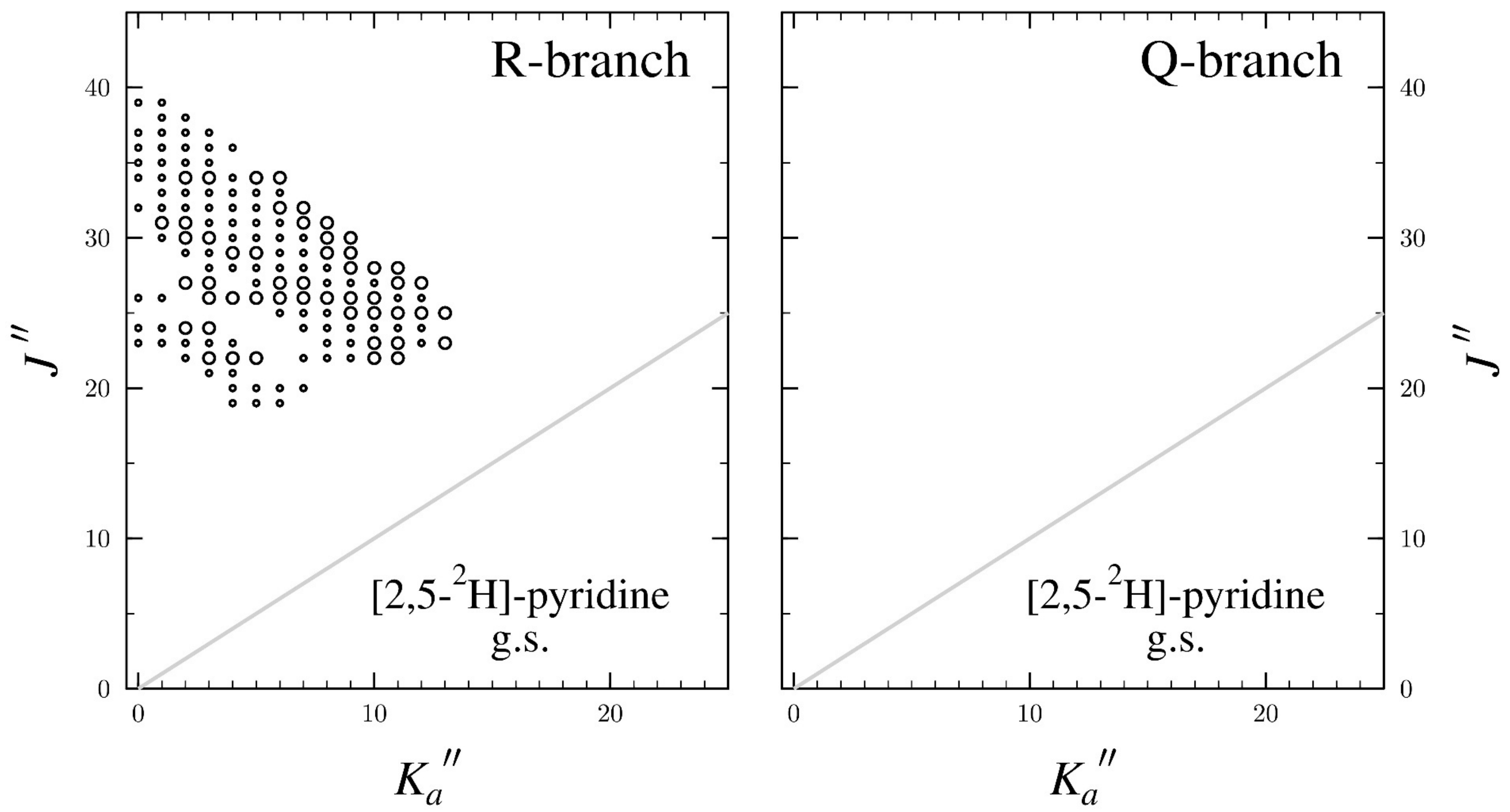

Figure S29. Data distribution plot for the least-squares fit of millimeter-wave spectroscopic data for [2,5-^{2}H]-pyridine, ground vibrational state. Black (all) circles are measurements from the current work. The size of the outlined circle is proportional to the value of $|(f_{obs.} - f_{calc.})/\delta f|$, where δf is the frequency measurement uncertainty (50 kHz), and no quotient values are larger than three.

Table S30. Experimental and computed spectroscopic constants for [2,6-^{2}H]-pyridine

	S Reduction, I^r representation			A Reduction, I^r representation	
	Experimental	CCSD(T) [a]		Experimental	CCSD(T) [a]
A_0 (MHz)	5598.540 44 (18)	5566	A_0 (MHz)	5598.539 04 (25)	5566
B_0 (MHz)	5482.370 84 (13)	5465	B_0 (MHz)	5482.371 84 (17)	5465
C_0 (MHz)	2769.420 651 (82)	2757	C_0 (MHz)	2769.420 02 (20)	2757
D_J (kHz)	0.370 447 (34)	0.368	Δ_J (kHz)	0.564 875 (54)	0.560
D_{JK} (kHz)	0.937 85 (19)	0.914	Δ_{JK} (kHz)	−0.229 14 (13)	−0.238
D_K (kHz)	−0.089 83 (17)	−0.086 7	Δ_K (kHz)	0.882 33 (21)	0.873
d_1 (kHz)	−0.235 209 (15)	−0.234	δ_J (kHz)	0.235 197 (14)	0.234
d_2 (kHz)	−0.097 246 8 (96)	−0.096 0	δ_K (kHz)	0.422 234 (40)	0.412
H_J (Hz)	[0.000 009 7]	0.000 009 7	Φ_J (Hz)	0.000 213 3 (30)	0.000 216
H_{JK} (Hz)	0.000 091 (16)	0.000 145	Φ_{JK} (Hz)	−0.000 435 (12)	−0.000 376
H_{KJ} (Hz)	0.001 215 (40)	0.001 18	Φ_{KJ} (Hz)	[−0.000 179]	−0.000 179
H_K (Hz)	[−0.000 829]	−0.000 829	Φ_K (Hz)	0.000 767 (71)	0.000 846
h_1 (Hz)	[0.000 065 9]	0.000 065 9	ϕ_J (Hz)	[0.000 108]	0.000 108
h_2 (Hz)	[0.000 103]	0.000 103	ϕ_{JK} (Hz)	[0.000 032 9]	0.000 032 9
h_3 (Hz)	[0.000 041 8]	0.000 041 8	ϕ_K (Hz)	[0.000 933]	0.000 933
N_{lines} [b]	938		N_{lines} [b]	938	
σ_{fit} (MHz)	0.039		σ_{fit} (MHz)	0.039	

[a] Evaluated using the cc-pCVTZ basis set. [b] Number of independent transitions.

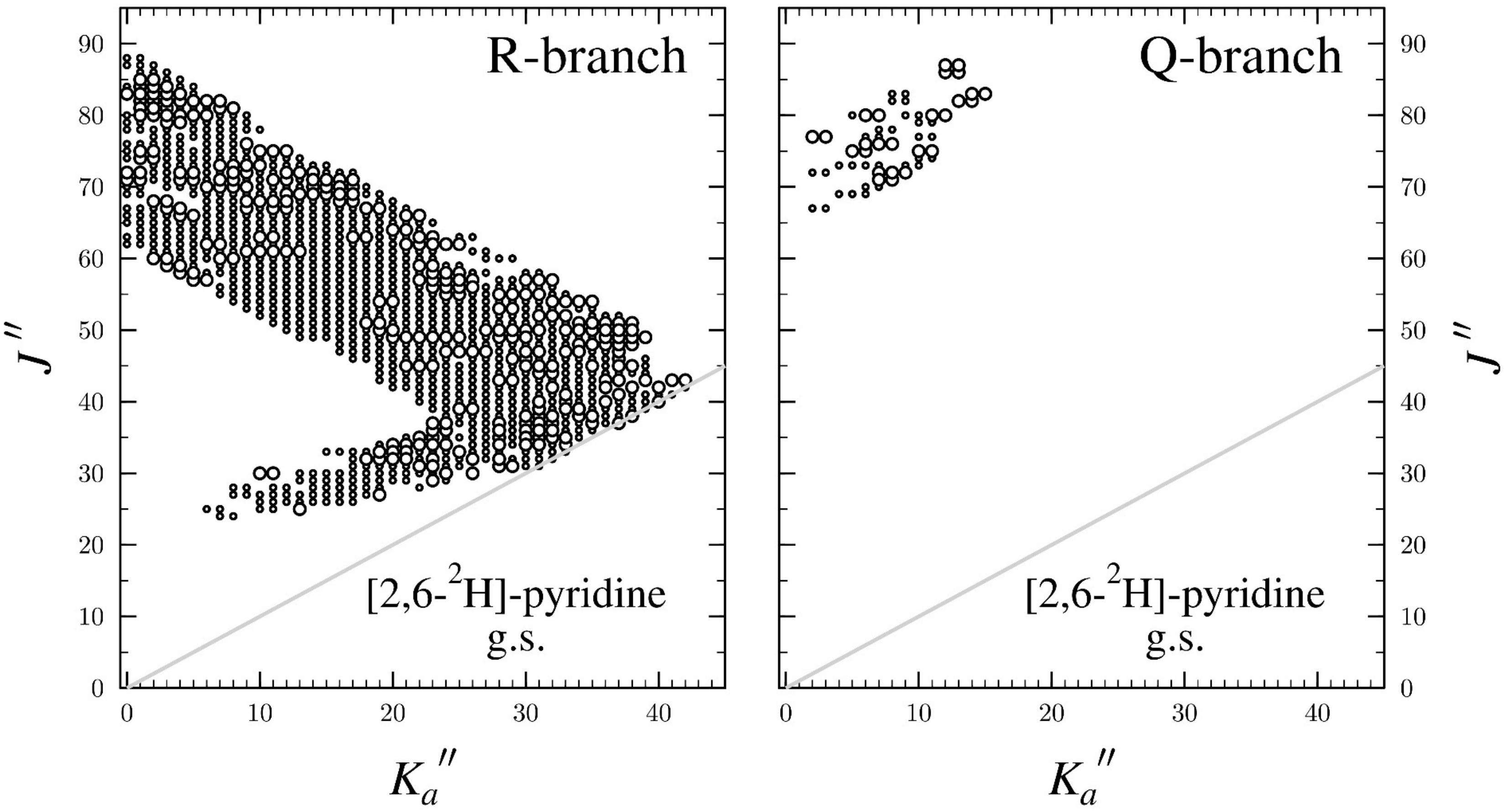

Figure S30. Data distribution plot for the least-squares fit of millimeter-wave spectroscopic data for [2,6-^{2}H]-pyridine, ground vibrational state. Black (all) circles are measurements from the current work. The size of the outlined circle is proportional to the value of $|(f_{obs.} - f_{calc.})/\delta f|$, where δf is the frequency measurement uncertainty (50 kHz), and no quotient values are larger than three.

Table S31. Experimental and computed spectroscopic constants for [3,4-^{2}H]-pyridine

	S Reduction, I^r representation			A Reduction, I^r representation	
	Experimental	CCSD(T) [a]		Experimental	CCSD(T) [a]
A_0 (MHz)	5781.746 58 (16)	5762	A_0 (MHz)	5781.745 59 (20)	5762
B_0 (MHz)	5292.276 25 (10)	5263	B_0 (MHz)	5292.277 63 (15)	5263
C_0 (MHz)	2762.578 63 (11)	2750	C_0 (MHz)	2762.578 09 (22)	2750
D_J (kHz)	0.391 524 (24)	0.386	Δ_J (kHz)	0.571 431 (55)	0.561
D_{JK} (kHz)	0.872 868 (80)	0.848	Δ_{JK} (kHz)	−0.206 394 (81)	−0.203
D_K (kHz)	−0.087 245 (96)	−0.065 3	Δ_K (kHz)	0.811 83 (21)	0.811
d_1 (kHz)	−0.238 071 6 (89)	−0.234	δ_J (kHz)	0.238 0670 (94)	0.234
d_2 (kHz)	−0.089 945 5 (60)	−0.087 7	δ_K (kHz)	0.499 053 (35)	0.491
H_J (Hz)	[−0.000 012 5]	−0.000 012 5	Φ_J (Hz)	0.000 200 1 (47)	0.000 198
H_{JK} (Hz)	0.000 810 (23)	0.000 733	Φ_{JK} (Hz)	0.000 193 (23)	0.000 123
H_{KJ} (Hz)	−0.000 652 (51)	−0.000 354	Φ_{KJ} (Hz)	−0.001 694 (55)	−0.001 47
H_K (Hz)	[0.000 104]	0.000 104	Φ_K (Hz)	0.001 418 (71)	0.001 62
h_1 (Hz)	[0.000 069 5]	0.000 069 5	ϕ_J (Hz)	[0.000 098 6]	0.000 098 6
h_2 (Hz)	[0.000 105]	0.000 105	ϕ_{JK} (Hz)	[0.000 255]	0.000 255
h_3 (Hz)	[0.000 029 1]	0.000 029 1	ϕ_K (Hz)	[0.000 838]	0.000 838
N_{lines} [b]	806		N_{lines} [b]	806	
σ_{fit} (MHz)	0.034		σ_{fit} (MHz)	0.034	

[a] Evaluated using the cc-pCVTZ basis set. [b] Number of independent transitions.

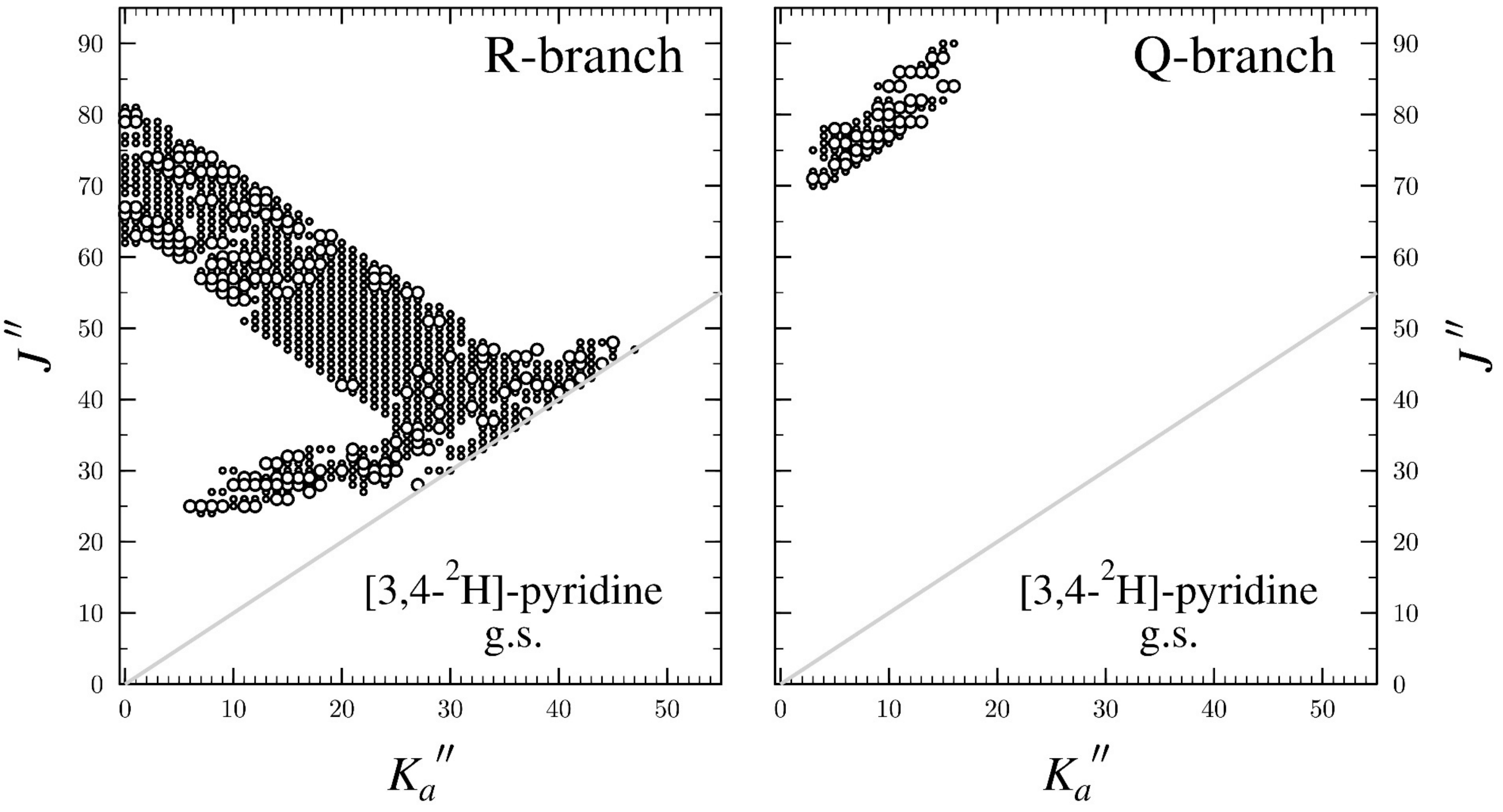

Figure S31. Data distribution plot for the least-squares fit of millimeter-wave spectroscopic data for [3,4-^{2}H]-pyridine, ground vibrational state. Black (all) circles are measurements from the current work. The size of the outlined circle is proportional to the value of $|(f_{obs.} - f_{calc.})/\delta f|$, where δf is the frequency measurement uncertainty (50 kHz), and no quotient values are larger than three.

Table S32. Experimental and computed spectroscopic constants for [3,5-^{2}H]-pyridine

	S Reduction, I^r representation			A Reduction, I^r representation	
	Experimental	CCSD(T) [a]		Experimental	CCSD(T) [a]
A_0 (MHz)	5620.175 86 (85)	5587	A_0 (MHz)	5620.174 93 (84)	5587
B_0 (MHz)	5434.287 00 (64)	5417	B_0 (MHz)	5434.288 19 (64)	5417
C_0 (MHz)	2762.311 21 (39)	2750	C_0 (MHz)	2762.310 80 (38)	2750
D_J (kHz)	0.380 22 (28)	0.378	Δ_J (kHz)	0.563 64 (21)	0.559
D_{JK} (kHz)	0.848 3 (15)	0.821	Δ_{JK} (kHz)	−0.252 25 (100)	−0.263
D_K (kHz)	0.014 8 (11)	0.017 3	Δ_K (kHz)	0.931 90 (66)	0.921
d_1 (kHz)	−0.234 474 (97)	−0.233	δ_J (kHz)	0.234 471 (98)	0.233
d_2 (kHz)	−0.091 712 (52)	−0.090 4	δ_K (kHz)	0.417 86 (23)	0.407
H_J (Hz)	[0.000 023 9]	0.000 023 9	Φ_J (Hz)	[0.000 214]	0.000 214
H_{JK} (Hz)	[0.000 084 4]	0.000 084 4	Φ_{JK} (Hz)	[−0.000 374]	−0.000 374
H_{KJ} (Hz)	[0.001 09]	0.001 09	Φ_{KJ} (Hz)	[−0.000 232]	−0.000 232
H_K (Hz)	[−0.000 669]	−0.000 669	Φ_K (Hz)	[0.000 925]	0.000 925
h_1 (Hz)	[0.000 068 8]	0.000 068 8	ϕ_J (Hz)	[0.000 107]	0.000 107
h_2 (Hz)	[0.000 095 1]	0.000 095 1	ϕ_{JK} (Hz)	[0.000 031 9]	0.000 031 9
h_3 (Hz)	[0.000 037 9]	0.000 037 9	ϕ_K (Hz)	[0.000 934]	0.000 934
N_{lines} [b]	202		N_{lines} [b]	202	
σ_{fit} (MHz)	0.041		σ_{fit} (MHz)	0.041	

[a] Evaluated using the cc-pCVTZ basis set. [b] Number of independent transitions.

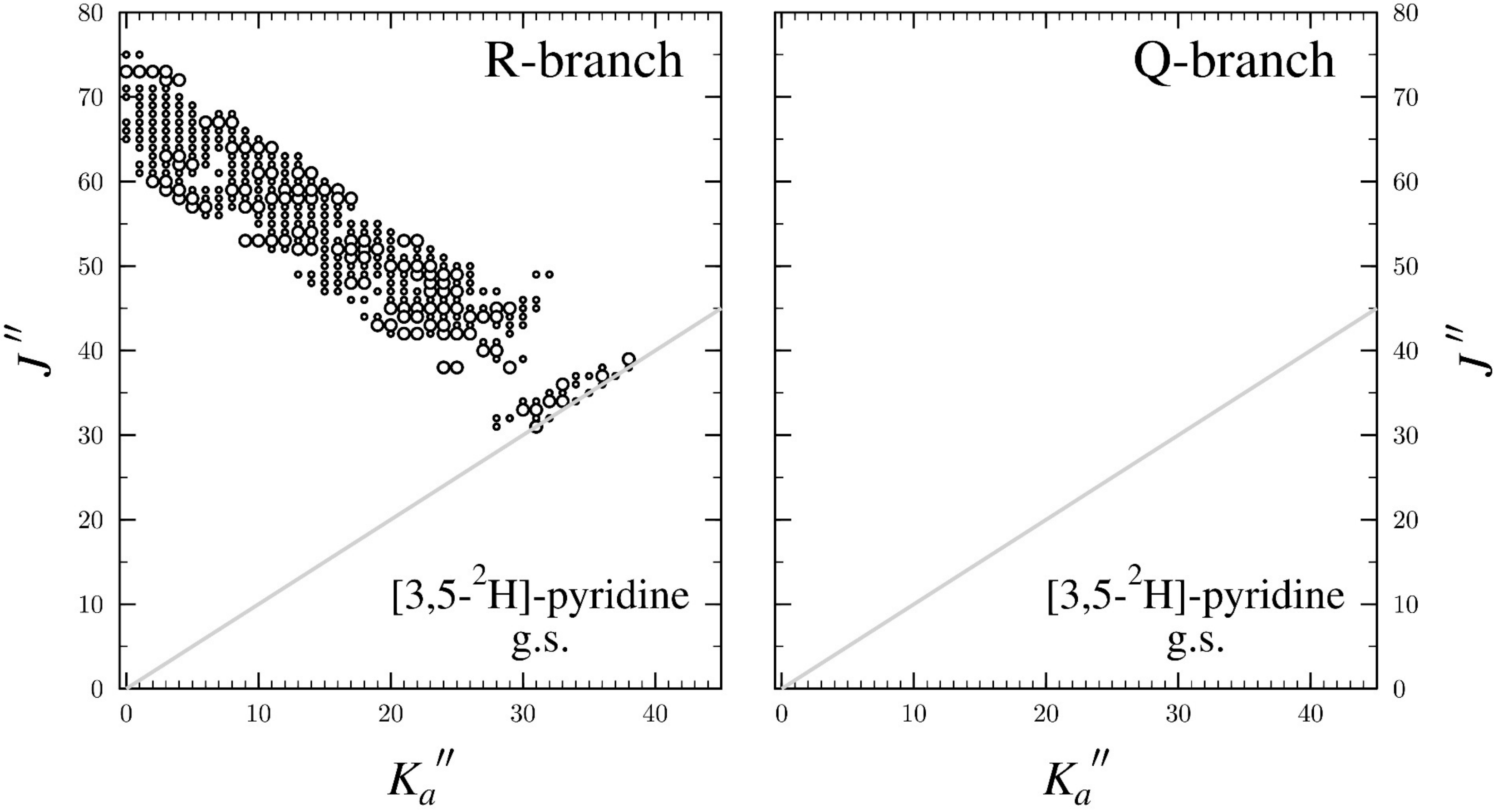

Figure S32. Data distribution plot for the least-squares fit of millimeter-wave spectroscopic data for [3,5-^{2}H]-pyridine, ground vibrational state. Black (all) circles are measurements from the current work. The size of the outlined circle is proportional to the value of $|(f_{obs.} - f_{calc.})/\delta f|$, where δf is the frequency measurement uncertainty (50 kHz), and no quotient values are larger than three.

Table S33. Differences between experimental determinable constants and those predicted by CCSD(T) and by *xrefit* [a]

Isotopologue	$A''_0 - A''_{CCSD(T)}$ (MHz)	$A''_0 - A''_{xrefit}$ (MHz)	$B''_0 - B''_{CCSD(T)}$ (MHz)	$B''_0 - B''_{xrefit}$ (MHz)	$C''_0 - C''_{CCSD(T)}$ (MHz)	$C''_0 - C''_{xrefit}$ (MHz)
C_5H_5N	19.2011	−0.0364	34.7122	−0.0169	13.8815	0.0015
[^{15}N]-C_5H_5N	19.2067	−0.0308	34.2990	−0.0255	13.8589	−0.0005
[2-^{13}C]-C_5H_5N	20.0675	0.0272	33.1888	−0.0504	13.6705	0.0085
[3-^{13}C]-C_5H_5N	20.3794	0.0203	33.1509	−0.0552	13.7332	0.0056
[4-^{13}C]-C_5H_5N	19.2072	−0.0303	33.9487	−0.0168	13.7688	0.0021
[2-^{13}C, ^{15}N]-C_5H_5N	19.3235	−0.0003	33.5439	−0.0245	13.6518	0.0071
[3-^{13}C, ^{15}N]-C_5H_5N	19.5770	−0.0128	33.5440	−0.0244	13.7105	0.0039
[4-^{13}C, ^{15}N]-C_5H_5N	19.2132	−0.0243	33.5406	−0.0238	13.7447	0.0008
[2-^{2}H]-C_5H_5N	25.7896	−0.0055	26.0795	−0.0525	13.2077	0.0023
[3-^{2}H]-C_5H_5N	26.5464	0.0222	25.7500	−0.0701	13.2970	0.0037
[4-^{2}H]-C_5H_5N	19.1661	−0.0713	31.8784	0.0030	13.3694	−0.0020
[3-^{2}H, 2-^{13}C]-C_5H_5N	26.2735	−0.8733	25.2946	0.7809	13.1055	0.0095
[3-^{2}H, 3-^{13}C]-C_5H_5N	27.3759	−0.3339	24.5798	0.2735	13.1667	0.0064
[3-^{2}H, 4-^{13}C]-C_5H_5N	23.4939	−0.0629	28.0992	0.0254	13.1976	0.0044
[3-^{2}H, 5-^{13}C]-C_5H_5N	28.0607	0.0274	23.8293	−0.0720	13.1570	0.0076
[3-^{2}H, 6-^{13}C]-C_5H_5N	27.7752	0.1139	23.9416	−0.1219	13.0936	0.0101
[2,3-^{2}H]-C_5H_5N	32.6589	−0.0544	17.5467	0.0004	12.6808	0.0060
[2,5-^{2}H]-C_5H_5N	28.7875	−0.0976	21.6609	0.0169	12.6619	0.0062
[2,6-^{2}H]-C_5H_5N	32.3391	−0.0232	17.4540	−0.0235	12.5865	0.0038
[3,4-^{2}H]-C_5H_5N	20.2055	−0.0426	29.3702	−0.0141	12.8323	0.0005
[3,5-^{2}H]-C_5H_5N	33.1316	−0.0221	17.5362	−0.0076	12.7510	0.0056
[2,4,6-^{2}H]-C_5H_5N	17.4630	−0.0150	29.7151	−0.0472	12.1471	−0.0003
[2,5,6-^{2}H]-C_5H_5N	29.8000	−0.0244	18.5629	−0.0156	12.0909	0.0059

Isotopologue	$A''_0 - A''_{CCSD(T)}$ (MHz)	$A''_0 - A''_{xrefit}$ (MHz)	$B''_0 - B''_{CCSD(T)}$ (MHz)	$B''_0 - B''_{xrefit}$ (MHz)	$C''_0 - C''_{CCSD(T)}$ (MHz)	$C''_0 - C''_{xrefit}$ (MHz)
[3,4,5-^{2}H]-C_5H_5N	17.5134	−0.0309	30.5827	−0.0092	12.3287	0.0029
[2,3,5,6-^{2}H]-C_5H_5N	30.8547	−0.0217	16.0620	−0.0066	11.6257	0.0069
[2,4,5,6-^{2}H]-C_5H_5N	22.0253	−0.0072	23.8568	−0.0460	11.6890	0.0009
[3,4,5,6-^{2}H]-C_5H_5N	23.4056	−0.0067	22.9410	−0.0391	11.7734	0.0026
[2,3,4,5,6-^{2}H]-C_5H_5N	28.5358	−0.0106	16.0401	−0.0284	11.2591	0.0032
[2,3,4,5,6-^{2}H, ^{15}N]-C_5H_5N	15.9951	−0.1020	28.3197	0.0545	11.2515	0.0010
[2,3,4,5,6-^{2}H, 2-^{13}C]-C_5H_5N	27.5447	0.0092	16.5605	−0.0262	11.1145	0.0089
[2,3,4,5,6-^{2}H, 3-^{13}C]-C_5H_5N	27.7037	0.0085	16.6137	−0.0357	11.1627	0.0062
[2,3,4,5,6-^{2}H, 4-^{13}C]-C_5H_5N	22.0000	0.0045	22.0822	−0.0384	11.1905	0.0043

[a] B''_0 = the averaged (between A- and S-reductions) experimental determinable constant. $B''_{CCSD(T)}$ = the averaged (between A- and S-reductions) predicted observable constants.

Table S34. Semi-experimental and best theoretical estimate equilibrium structures of pyridine using variable numbers of isotopologues for current r_e^{SE}

Parameter	$r_e^{SE\ a}$ This work	$r_e^{SE\ a}$ This work	$r_e^{SE\ a}$ This work	r_e^{SE} Császár *et al.*[13, a]	r_e^{SE} Picarrdo *et al.*[14, a]	r_e^{SE} Pennochio *et al.*[15, a]	r_e BTE, This work
$R_{C2–H}$ (Å)	1.0821 (1)	1.0822 (1)	1.0822 (3)	1.0816 (8)	1.0818 (4)	1.0821 (4)	1.0824
$R_{C3–H}$ (Å)	1.0801 (1)	1.0801 (1)	1.0801 (3)	1.0795 (8)	1.0796 (4)	1.0799 (4)	1.0802
$R_{C4–H}$ (Å)	1.0806 (2)	1.0806 (1)	1.0807 (2)	1.0803 (8)	1.0802 (4)	1.0806 (4)	1.0808
$R_{C2–C3}$ (Å)	1.3905 (2)	1.3905 (2)	1.3906 (3)	1.3902 (8)	1.3907 (4)	1.3907 (4)	1.3906
$R_{C3–C4}$ (Å)	1.3883 (2)	1.3883 (1)	1.3883 (3)	1.3890 (8)	1.3888 (4)	1.3885 (4)	1.3886
$R_{C4–N}$ (Å)	2.7980 (2)	2.7980 (2)	2.7979 (3)				2.7988
$\theta_{C2–C3–C4}$ (°)	118.544 (14)	118.542 (12)	118.537 (24)	118.54 (8)	118.53 (2)	118.54 (2)	118.544
$\theta_{C3–C2–H}$ (°)	120.316 (16)	120.316 (14)	120.322 (38)	120.30 (12)	120.25 (4)	120.24 (4)	120.301
$\theta_{C4–C3–H}$ (°)	121.332 (15)	121.332 (12)	121.311 (35)	121.34 (10)	121.37 (4) [b]	121.37 (4) [b]	121.317
$\theta_{C3–C4–H}$ (°)	120.778 (8)	120.779 (7)	120.774 (13)	120.71 (4)	120.78 (2)	120.78 (2)	120.782
Number of isotopologues	32	31	8	10	8	8	

[a] Uncertainties reported are multiplied by two to achieve 2σ.

Materials and Synthesis. Pyridine, [^{15}N]-pyridine, and [2,3,4,5,6-^{2}H]-pyridine were obtained commercially and used for analysis without further purification. These samples were sufficiently pure to observe all singly substituted heavy-atom isotopologues at natural abundance. The sample of [2,3,4,5,6-^{2}H]-pyridine (pyridine-d_5) was sufficiently *impure* to observe all three pyridine-d_4 isotopologues as minor contaminants.

Various synthetic procedures, along with good fortune, enabled the spectroscopic observation of 16 of the 20 possible deuterium-containing isotopologues of pyridine (see Scheme S3, below). The [2-^{2}H] and [3-^{2}H] isotopologues of pyridine were prepared from commercially available 2-bromopyridine or 3-bromopyridine by lithium-halogen exchange, followed by quenching with D_2O (Scheme S1). The lithiation chemistry of halopyridines is not necessarily clean, owing to rearrangements and other competing processes.[16-17] In our hands, the reaction of 2-bromopyridine afforded a sample that contained not only the intended mono-substituted product, [2-^{2}H]-pyridine, but also a small amount of two di-substituted products, [2,3-^{2}H]- and [2,5-^{2}H]-

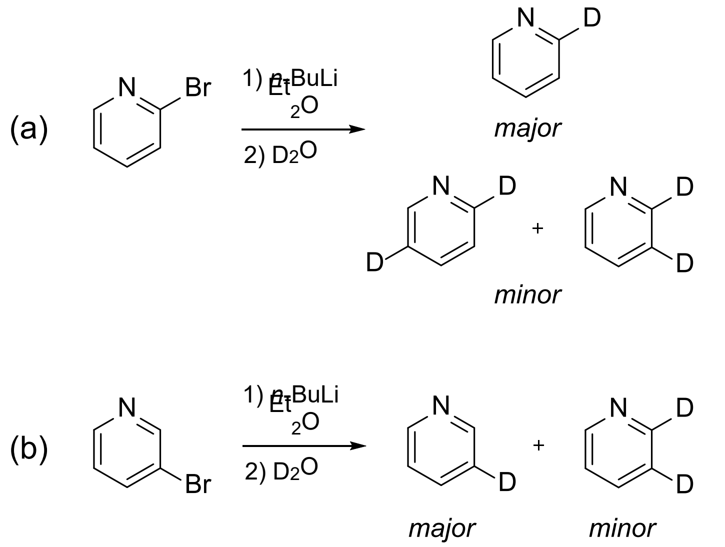

Scheme S1. Lithium-halogen exchange reactions, followed by quenching with D_2O, used to prepare deuterium-containing isotopologues of pyridine.

pyridine (Scheme S1a). The reaction of 3-bromopyridine afforded a sample of sufficient quantity and purity of [3-^{2}H]-pyridine (Scheme S1b) to observe all singly substituted heavy-atom isotopologues at natural abundance.

Base-catalyzed H/D exchange reactions of pyridine-h_5 with Me_2SO-d_6 (Scheme S2a) and of pyridine-d_5 with Me_2SO-h_6 (Scheme S2b) were employed, based upon the methodology of Li *et al.*,[18] which effects isotopic exchange at positions 3,4,5 but not at positions 2,6.

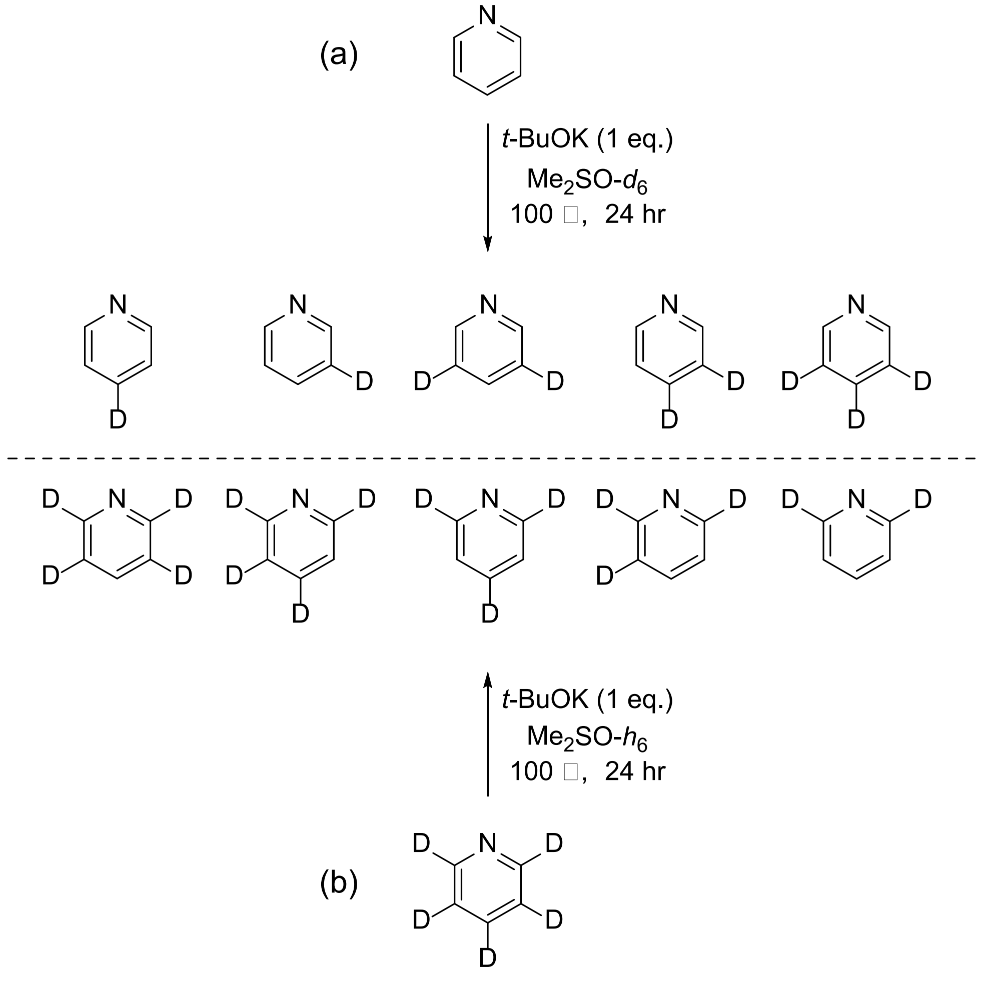

Scheme S2. Hydrogen-deuterium exchange reactions used to obtain various deuterium-containing isotopologues of pyridine.

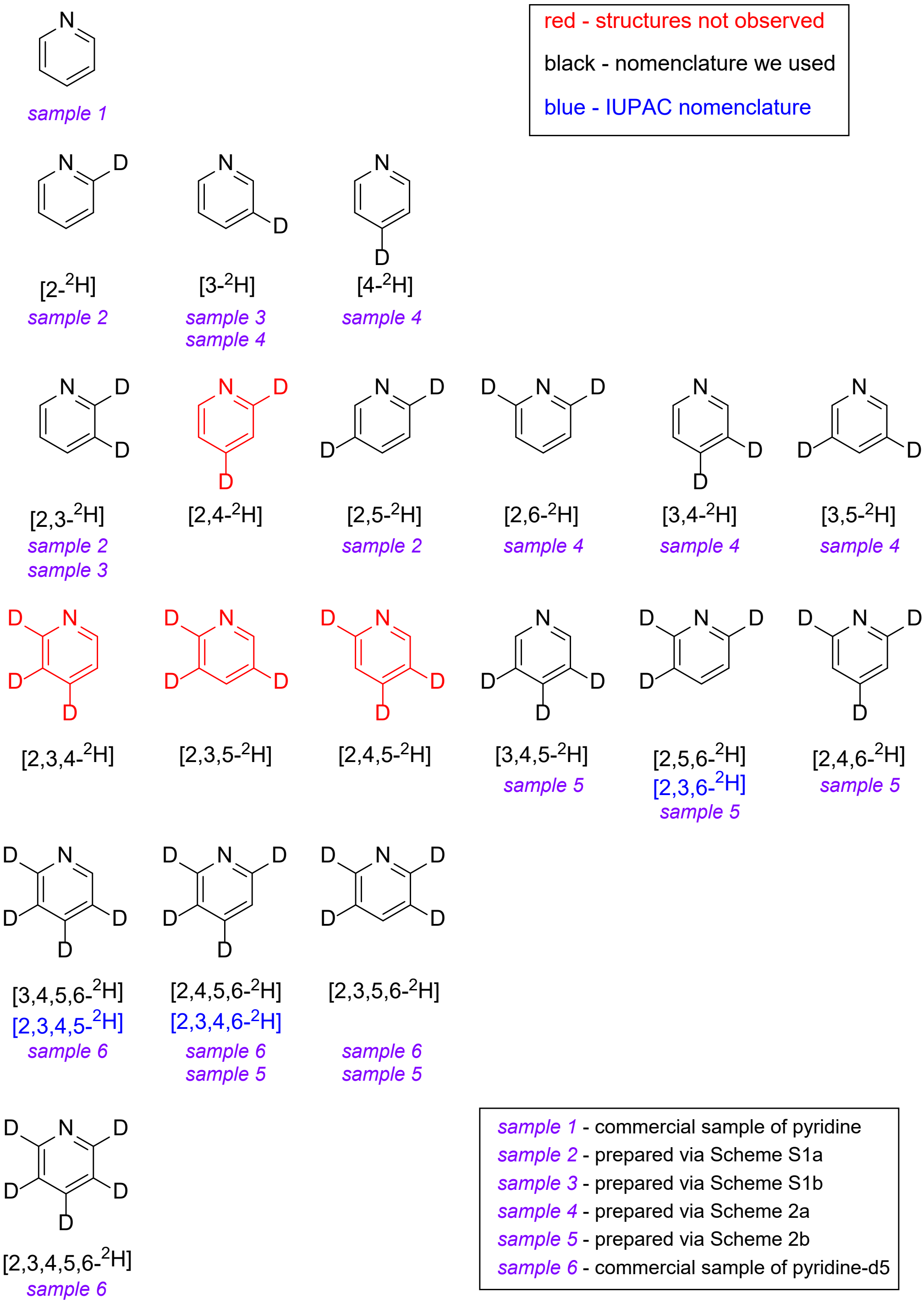

Scheme S3. Structures of all possible deuterium-containing isotopologues, along with their name / numbering and the sample from which they were observed. The numbering convention that we used does not always adhere to IUPAC guidelines for using the numbering sequence that gives the lowest possible numbers because those guidelines result in non-systematic changes in naming upon introduction of isotopic species at various positions. Those changes disrupt the logical categorization and ordering within a family of structurally related isotopologues.

Experimental Procedures for Pyridine Isotopologues

[2-^{2}H]-Pyridine. In an oven-dried 100-mL round-bottom flask (cooled and maintained under an atmosphere of dry N_2), 2-bromopyridine (8.0104 g, 50.6 mmol) was added in 35 mL dry diethyl ether. The mixture was cooled to 0 ºC on an ice bath, and *n*-BuLi (11.0 M in hexanes, 7 mL, 77 mmol, 1.5 equiv.) was added dropwise to the stirred reaction. The reaction turned rust-colored as the *n*-BuLi was added. After the addition was completed, the reaction was stirred at 0 ºC for an additional 5 min. Deuterium oxide (8.0 mL, 8.8 g, 440 mmol) was added dropwise to the stirred reaction flask at 0 ºC. The addition of D_2O results in an exotherm, the rate of addition was performed sufficiently slow to maintain the 0 ºC reaction temperature. After the addition of D_2O was complete, the reaction vessel was removed from the cooling bath and allowed to warm to room temperature. The reaction was extracted with diethyl ether (4 × 15 mL), the organic extract was dried over anhydrous $MgSO_4$, and filtered. The volume of the dried extract was reduced to approximately 25 mL by rotary evaporation. The remaining solvent was distilled away using a short path distillation rig at atmospheric pressure. The product ([2-^{2}H]-pyridine) was distilled at atmospheric pressure (bp = 100-115 ºC) as a colorless oil (937 mg, 23 %). HRMS (ASAP-MS) *m/z*: $[M+H]^+$ Calcd for C_5H_5DN: 81.0558. Found: 81.0556. Subsequent analysis of the sample by rotational spectroscopy revealed the presence of small amounts of the [2,3-^{2}H] and [2,5-^{2}H] isotopologues, in addition to [2-^{2}H]-pyridine.

[3-^{2}H]-Pyridine. In an oven-dried 100-mL round-bottom flask (cooled and maintained under an atmosphere of dry N_2), 2-bromopyridine (8.3335 g, 52.7 mmol) was added in 35 mL dry diethyl ether. The mixture was cooled to 0 ºC on an ice bath, and *n*-BuLi (11.0 M in hexanes, 7 mL, 77 mmol, 1.5 equiv.) was added dropwise to the stirred reaction. The reaction turned rust-colored as the *n*-BuLi was added. After the addition was completed, the reaction was stirred at 0 ºC for an additional 5 min. Deuterium oxide (8.0 mL, 8.8 g, 440 mmol) was added dropwise to the

stirred reaction flask at 0 ºC. The addition of D_2O results in an exotherm, the rate of addition was performed sufficiently slow to maintain the 0 ºC reaction temperature. After the addition of D_2O was complete, the reaction vessel was removed from the cooling bath and allowed to warm to room temperature. The reaction was extracted with diethyl ether (4 × 15 mL), the organic extract was dried over anhydrous $MgSO_4$, and filtered. The volume of the dried extract was reduced to approximately 25 mL by rotary evaporation. The remaining solvent was distilled away using a short path distillation rig at atmospheric pressure. The product ([3-^{2}H]-pyridine) was distilled at atmospheric pressure (bp = 100-115 ºC) as a colorless oil (725 mg, 17 %). HRMS (ASAP-MS) *m/z*: $[M+H]^+$ Calcd for C_5H_5DN: 81.0558. Found: 81.0556. Subsequent analysis of the sample by rotational spectroscopy revealed the presence of a small amount of the [2,3-^{2}H] isotopologue, in addition to [3-^{2}H]-pyridine.

Deuterio-pyridine isotopologues *via* isotopic exchange of pyridine-h_5 and pyridine-d_5.[18] In a flame-dried 50-mL glass pressure vessel equipped with a stir bar were combined 1.1468 g pyridine (14.5 mmol, 1.2 eq.), 1.4452 g *t*-BuOK (13 mmol, 1 eq.), and 20 mL MeSO-d_6 (Scheme S2a). The flask was sealed, and the solution was rapidly stirred at 100 °C for 24 h. The solid dissolved readily upon heating, and the solution darkened from clear to pale yellow over the reaction period. After this period, the solution was diluted in 300 mL deionized H_2O, then extracted three times with 100 mL CH_2Cl_2. The combined organic layers were washed twice with 100 mL brine, dried over Na_2SO_4, vacuum filtered, and CH_2Cl_2 removed via rotary evaporation, yielding a clear and distinct-smelling oil. The remaining CH_2Cl_2 was removed by fractional distillation using a short-path distillation apparatus at ambient pressure. The product was then distilled through the same apparatus to yield 0.8079 g (~70% yield) of colorless oil. The resulting oil consisted of multiple deuterium-containing pyridine isotopologues. The level of deuterium incorporation was estimated by NMR and mass spectrometry. Subsequent analysis of the sample by rotational

spectroscopy revealed the presence of [3-^{2}H], [4-^{2}H], [3,4-^{2}H], [3,5-^{2}H], and [3,4,5-^{2}H]-pyridine isotopologues.

The same procedure was replicated using the complementary set of reagents – pyridine-d_5 and Me_2SO-h_6 (Scheme S2b). In this instance, the product consisted of multiple deuterium-containing pyridine isotopologues with higher levels of deuterium incorporation. The level of deuterium incorporation was estimated by NMR and mass spectrometry. Subsequent analysis of the sample by rotational spectroscopy revealed the presence of [2,4,5,6-^{2}H], [2,3,5,6-^{2}H], [2,5,6-^{2}H], [2,4,6-^{2}H], and [2,6-^{2}H]-pyridine isotopologues.

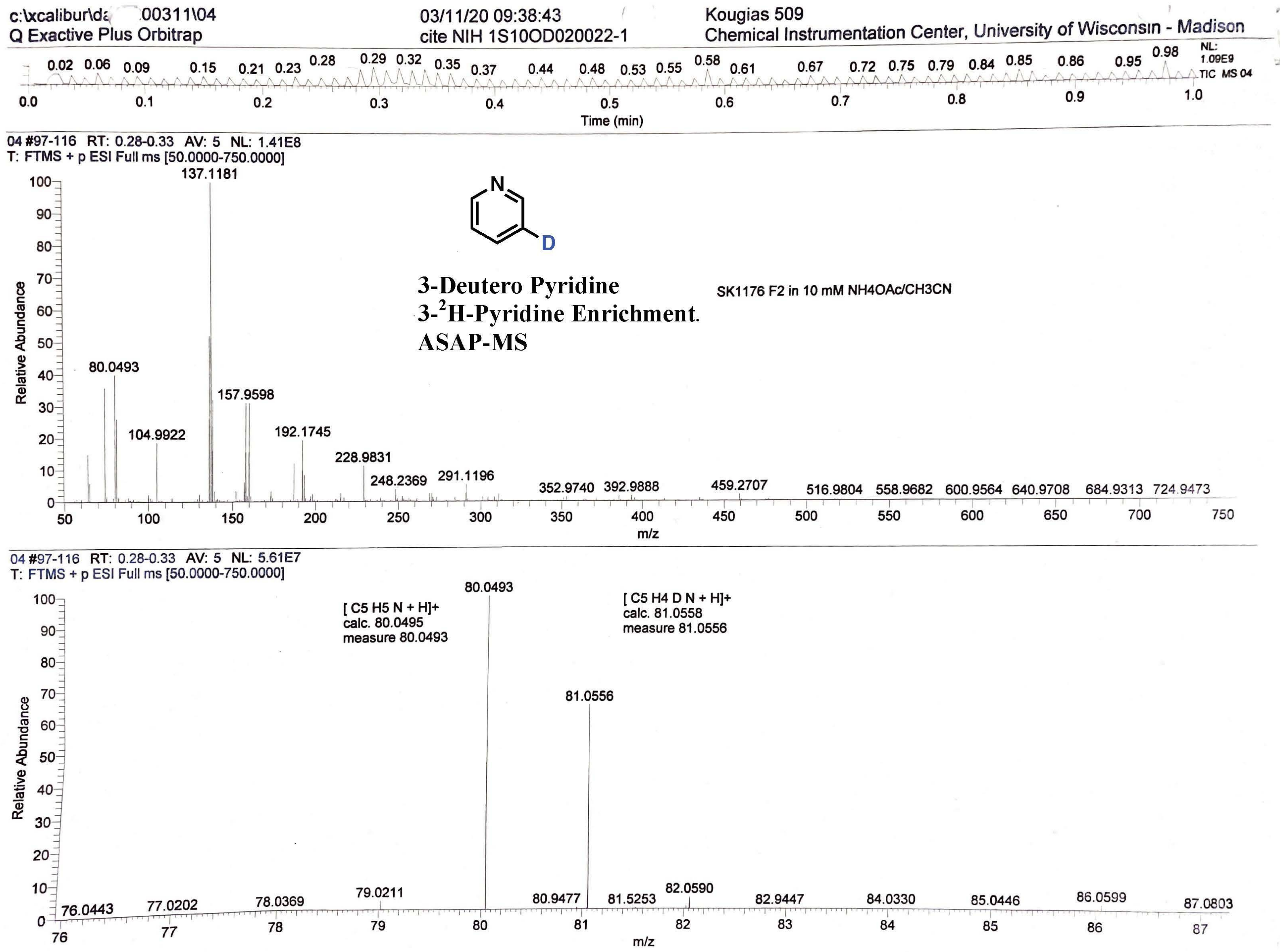
c:\xcalibur\da[illegible].00311\04
Q Exactive Plus Orbitrap
03/11/20 09:38:43
cite NIH 1S10OD020022-1
Kougias 509
Chemical Instrumentation Center, University of Wisconsin - Madison
NL: 1.09E9
TIC MS 04
Time (min)
04 #97-116 RT: 0.28-0.33 AV: 5 NL: 1.41E8
T: FTMS + p ESI Full ms [50.0000-750.0000]
Relative Abundance
137.1181
80.0493
104.9922
157.9598
192.1745
228.9831
248.2369
291.1196
352.9740
392.9888
459.2707
516.9804
558.9682
600.9564
640.9708
684.9313
724.9473
m/z
D
3-Deutero Pyridine
3-²H-Pyridine Enrichment.
ASAP-MS
SK1176 F2 in 10 mM NH4OAc/CH3CN
04 #97-116 RT: 0.28-0.33 AV: 5 NL: 5.61E7
T: FTMS + p ESI Full ms [50.0000-750.0000]
[C5 H5 N + H]+
calc. 80.0495
measure 80.0493
[C5 H4 D N + H]+
calc. 81.0558
measure 81.0556
80.0493
81.0556
76.0443
77.0202
78.0369
79.0211
80.9477
81.5253
82.0590
82.9447
84.0330
85.0446
86.0599
87.0803
m/z

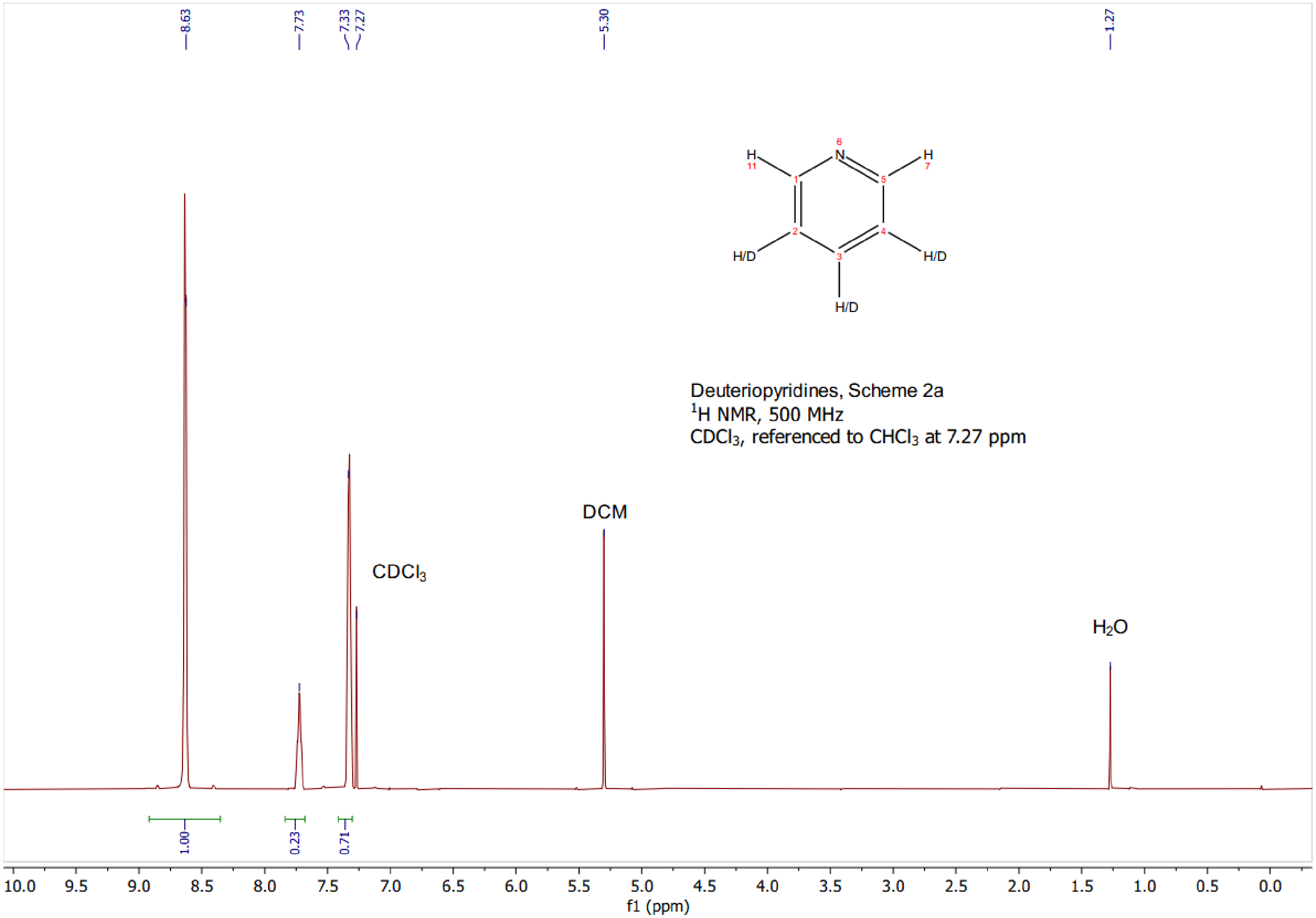

8.63
7.73
7.33
7.27
5.30
1.27
H
N
H
H/D
H/D
H/D
Deuteriopyridines, Scheme 2a
1H NMR, 500 MHz
CDCl3, referenced to CHCl3 at 7.27 ppm
DCM
CDCl3
H2O
1.00
0.23
0.71
10.0 9.5 9.0 8.5 8.0 7.5 7.0 6.5 6.0 5.5 5.0 4.5 4.0 3.5 3.0 2.5 2.0 1.5 1.0 0.5 0.0
f1 (ppm)

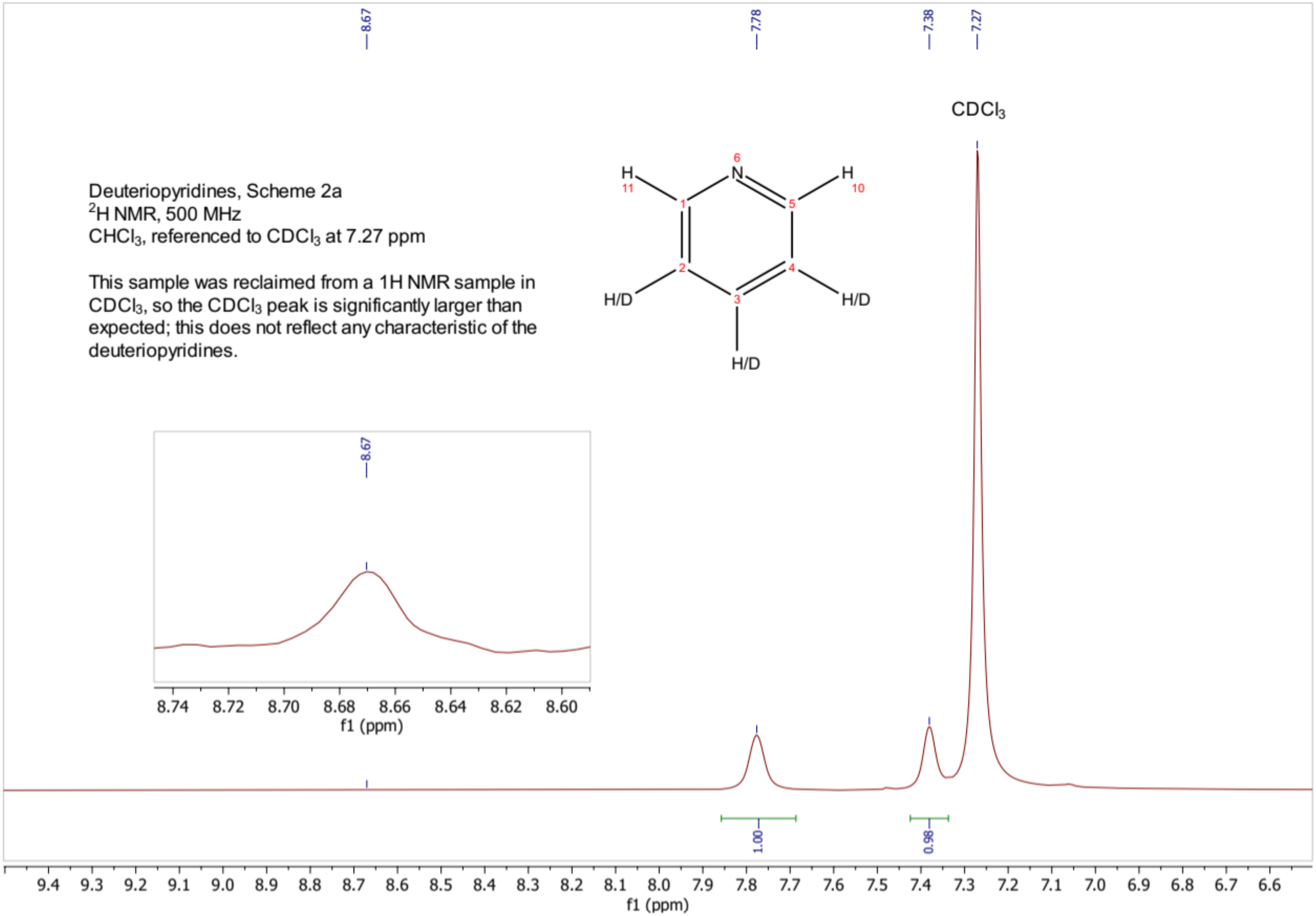
Deuteriopyridines, Scheme 2a
2H NMR, 500 MHz
CHCl3, referenced to CDCl3 at 7.27 ppm
This sample was reclaimed from a 1H NMR sample in CDCl3, so the CDCl3 peak is significantly larger than expected; this does not reflect any characteristic of the deuteriopyridines.
CDCl3
8.67
7.78
7.38
7.27
1.00
0.98
H/D
H/D
H/D
f1 (ppm)

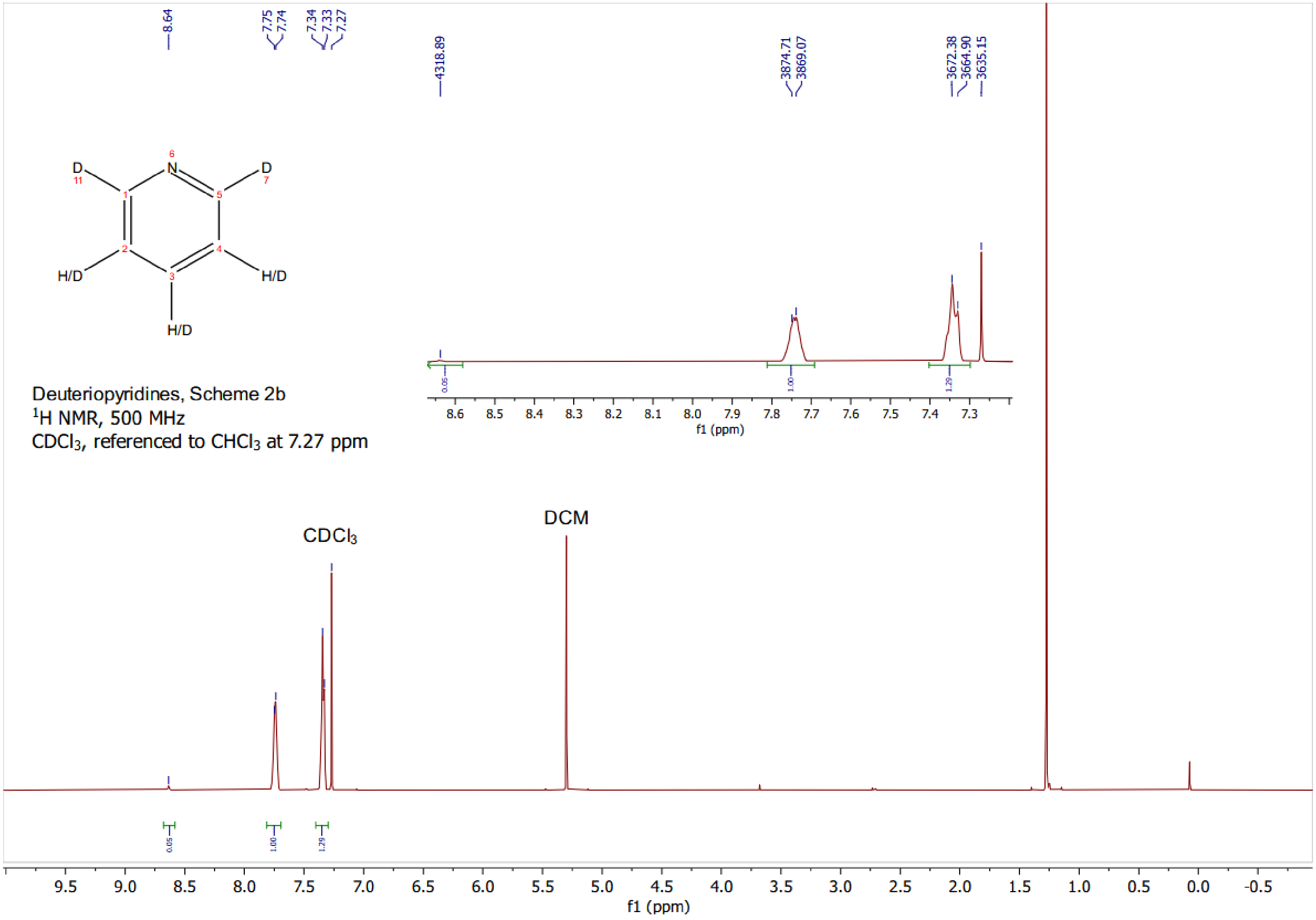
8.64
7.75
7.74
7.34
7.33
7.27
4318.89
3874.71
3869.07
3672.38
3664.90
3635.15
D
N
D
H/D
H/D
H/D
Deuteriopyridines, Scheme 2b
^{1}H NMR, 500 MHz
$CDCl_3$, referenced to $CHCl_3$ at 7.27 ppm
$CDCl_3$
DCM
f1 (ppm)

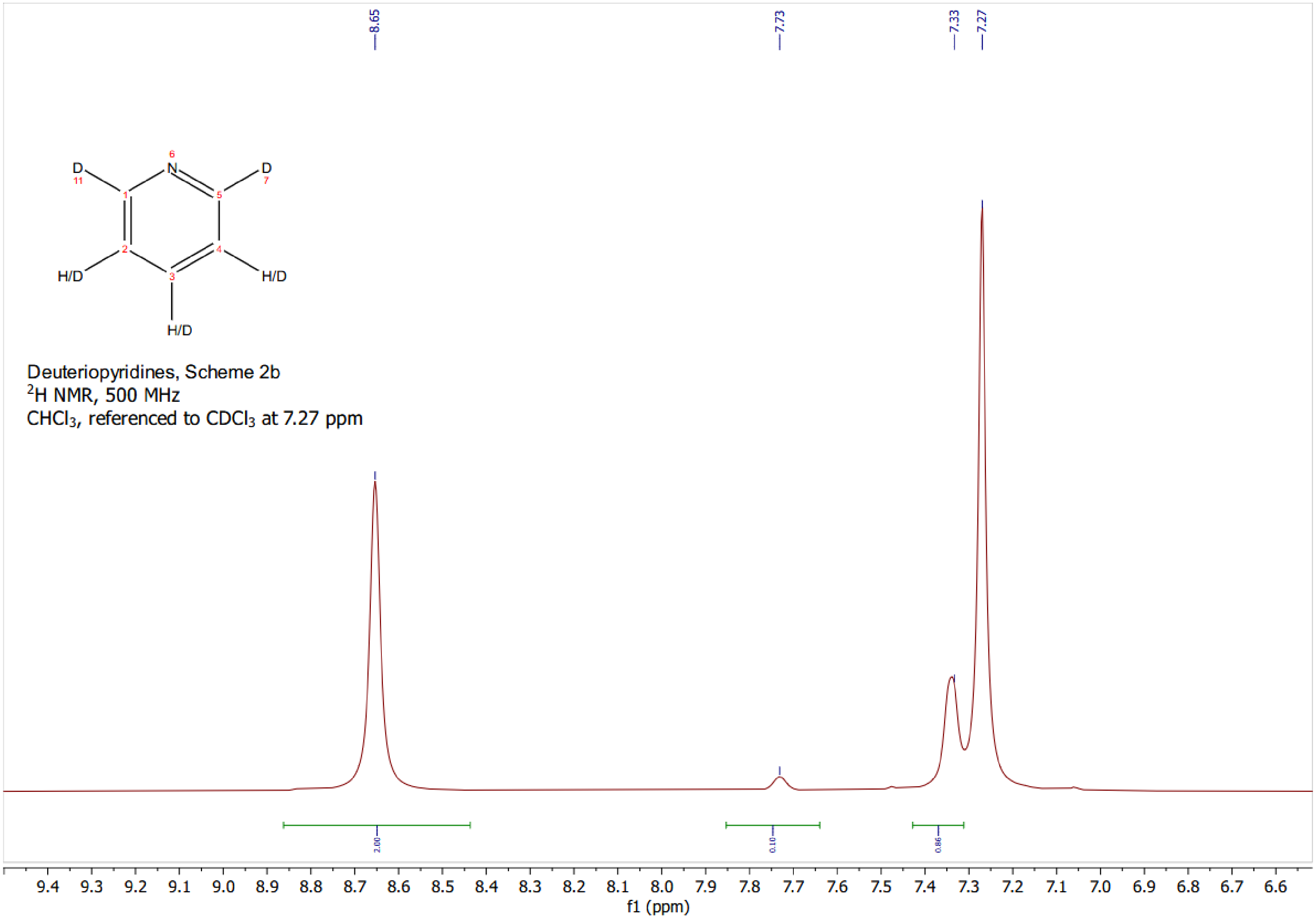

8.65
7.73
7.33
7.27
D
N
D
H/D
H/D
H/D
Deuteriopyridines, Scheme 2b
²H NMR, 500 MHz
CHCl3, referenced to CDCl3 at 7.27 ppm
2.00
0.10
0.86
9.4 9.3 9.2 9.1 9.0 8.9 8.8 8.7 8.6 8.5 8.4 8.3 8.2 8.1 8.0 7.9 7.8 7.7 7.6 7.5 7.4 7.3 7.2 7.1 7.0 6.9 6.8 6.7 6.6
f1 (ppm)

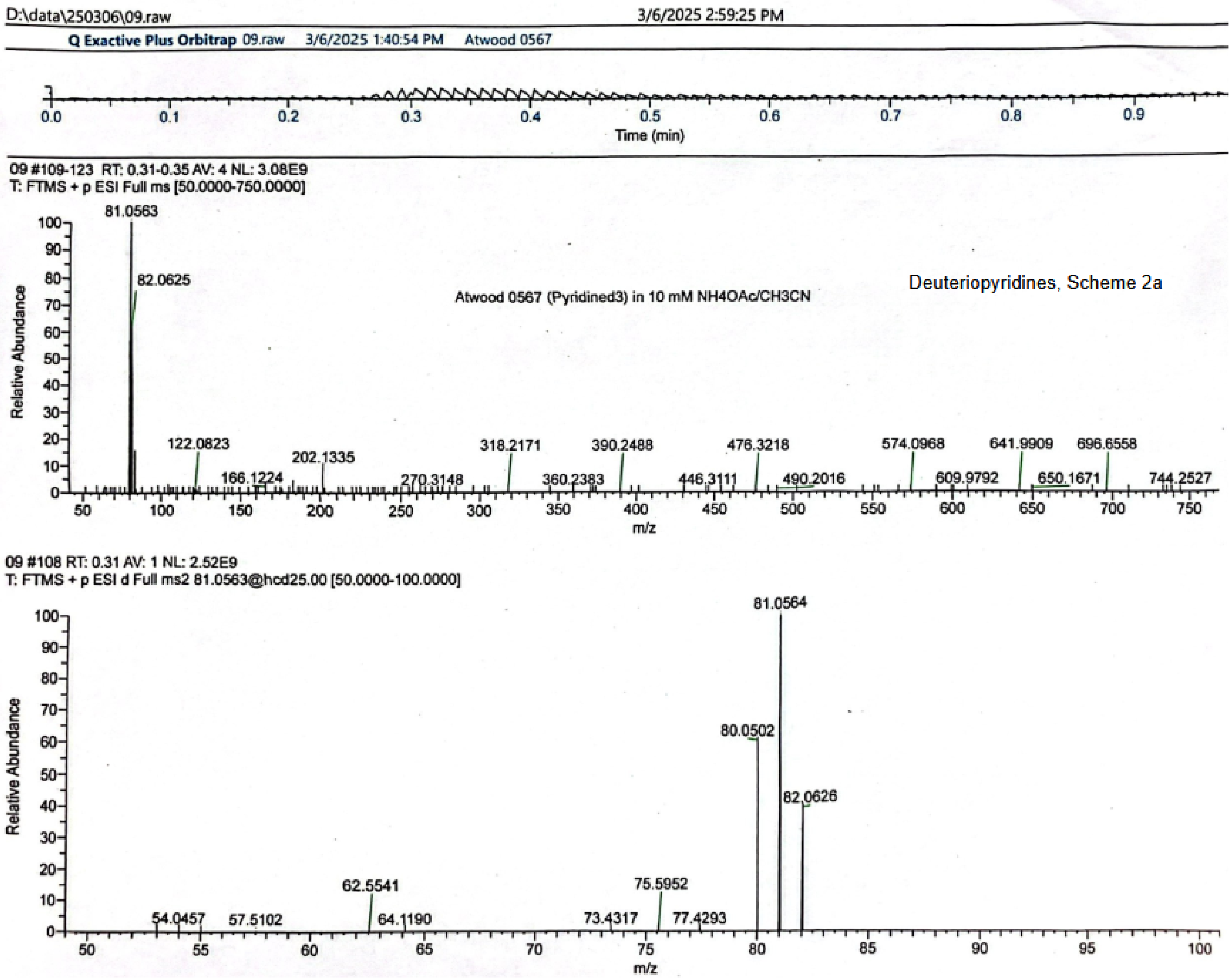
D:\data\250306\09.raw
3/6/2025 2:59:25 PM
Q Exactive Plus Orbitrap 09.raw 3/6/2025 1:40:54 PM Atwood 0567
Time (min)
09 #109-123 RT: 0.31-0.35 AV: 4 NL: 3.08E9
T: FTMS + p ESI Full ms [50.0000-750.0000]
81.0563
82.0625
Atwood 0567 (Pyridined3) in 10 mM NH4OAc/CH3CN
Deuteriopyridines, Scheme 2a
122.0823
166.1224
202.1335
270.3148
318.2171
360.2383
390.2488
446.3111
476.3218
490.2016
574.0968
609.9792
641.9909
650.1671
696.6558
744.2527
Relative Abundance
m/z
09 #108 RT: 0.31 AV: 1 NL: 2.52E9
T: FTMS + p ESI d Full ms2 81.0563@hcd25.00 [50.0000-100.0000]
81.0564
80.0502
82.0626
62.5541
64.1190
54.0457
57.5102
73.4317
75.5952
77.4293
Relative Abundance
m/z

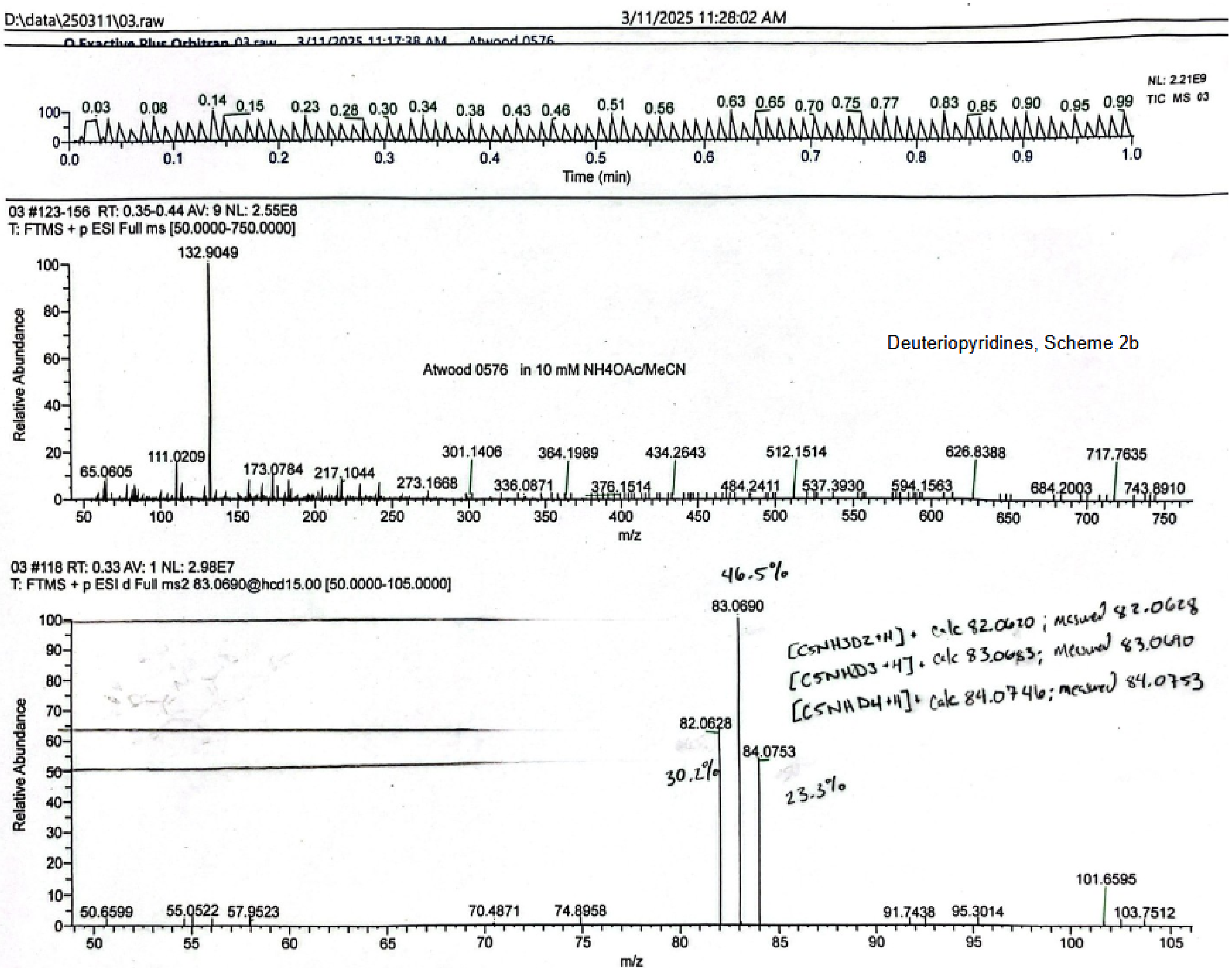
D:\data\250311\03.raw
3/11/2025 11:28:02 AM
NL: 2.21E9
TIC MS 03
Time (min)
03 #123-156 RT: 0.35-0.44 AV: 9 NL: 2.55E8
T: FTMS + p ESI Full ms [50.0000-750.0000]
132.9049
Relative Abundance
Deuteriopyridines, Scheme 2b
Atwood 0576 in 10 mM NH4OAc/MeCN
65.0605
111.0209
173.0784
217.1044
273.1668
301.1406
336.0871
364.1989
376.1514
434.2643
484.2411
512.1514
537.3930
594.1563
626.8388
684.2003
717.7635
743.8910
m/z
03 #118 RT: 0.33 AV: 1 NL: 2.98E7
T: FTMS + p ESI d Full ms2 83.0690@hcd15.00 [50.0000-105.0000]
46.5%
83.0690
[C5NH3D2+H]+ calc 82.0620; measured 82.0628
[C5NHD3+H]+ calc 83.0683; measured 83.0690
[C5NHD4+H]+ calc 84.0746; measured 84.0753
82.0628
84.0753
30.2%
23.3%
50.6599
55.0522
57.9523
70.4871
74.8958
91.7438
95.3014
101.6595
103.7512

References

(1) Puzzarini, C.; Bloino, J.; Tasinato, N.; Barone, V., Accuracy and Interpretability: The Devil and the Holy Grail. New Routes across Old Boundaries in Computational Spectroscopy. *Chem. Rev.* **2019**, *119*, 8131-8191.

(2) Bomble, Y. J.; Stanton, J. F.; Kallay, M.; Gauss, J., Coupled-Cluster Methods Including Noniterative Corrections for Quadruple Excitations. *J. Chem. Phys.* **2005**, *123*, 054101.

(3) Cheng, L.; Gauss, J., Analytic energy gradients for the spin-free exact two-component theory using an exact block diagonalization for the one-electron Dirac Hamiltonian. *J. Chem. Phys.* **2011**, *135*, 084114.

(4) Dyall, K. G., Interfacing relativistic and nonrelativistic methods. II. Investigation of a low-order approximation. *J. Chem. Phys.* **1998**, *109*, 4201-4208.

(5) Liu, W.; Peng, D., Exact two-component Hamiltonians revisited. *J. Chem. Phys.* **2009**, *131*, 031104.

(6) Born, M.; Huang, K., *Dynamical Theory of Crystal Lattices*. Oxford Univ. Press: 1954; p 430.

(7) Ye, E.; Bettens, R. P. A.; De Lucia, F. C.; Petkie, D. T.; Albert, S., Millimeter and submillimeter wave rotational spectrum of pyridine in the ground and excited vibrational states. *J. Mol. Spectrosc.* **2005**, *232*, 61-65.

(8) Sørensen, G. O.; Mahler, L.; Rastrup-Andersen, N., Microwave spectra of [^{15}N] and [^{13}C] pyridines, quadrupole coupling constants, dipole moment and molecular structure of pyridine. *J. Mol. Struct.* **1974**, *20*, 119-126.

(9) Bettens, R. P. A.; Bauder, A., The microwave spectrum and structure of the pyridine–CO complex. *J. Chem. Phys.* **1995**, *102*, 1501-1509.

(10) Bak, B.; Hansen, L.; Rastrup-Andersen, J., Microwave Determination of the Structure of Pyridine. *J. Chem. Phys.* **1954**, *22*, 2013-2017.

(11) Bak, B.; Hansen-Nygaard, L.; Rastrup-Andersen, J., Complete determination of the structure of pyridine by microwave spectra. *J. Mol. Spectrosc.* **1958**, *2*, 361-368.

(12) Heineking, N.; Dreizler, H.; Schwarz, R., Nitrogen and deuterium hyperfine structure in the rotational spectra of pyridine and [4-D]pyridine. *Z. Naturforsch., A: Phys., Phys. Chem., Kosmophys.* **1986**, *41A*, 1210-13.

(13) Császár, A. G.; Demaison, J.; Rudolph, H. D., Equilibrium Structures of Three-, Four-, Five-, Six-, and Seven-Membered Unsaturated *N*-Containing Heterocycles. *J. Phys. Chem. A* **2015**, *119*, 1731-1746.

(14) Piccardo, M.; Penocchio, E.; Puzzarini, C.; Biczysko, M.; Barone, V., Semi-Experimental Equilibrium Structure Determinations by Employing B3LYP/SNSD Anharmonic Force Fields: Validation and Application to Semirigid Organic Molecules. *J. Phys. Chem. A* **2015**, *119*, 2058-2082.

(15) Penocchio, E.; Piccardo, M.; Barone, V., Semiexperimental Equilibrium Structures for Building Blocks of Organic and Biological Molecules: The B2PLYP Route. *J. Chem. Theory Comput.* **2015**, *11*, 4689-4707.

(16) Mallet, M.; Quénguiner, G., Reaction de la bromo-3 pyridine avec le diisopropylamidure de lithium. Mecanismes de metallation et de migration d'halogene. Regioselectivite de l'addition polaire sur la pyridyne-3,4. *Tetrahedron* **1982**, *38*, 3035-3042.

(17) Mallet, M.; Queguiner, G., Homotransmetallation des halogeno-3 pyridines bromees en -2 ou -4 par le n-butyllithium. Proposition d'un nouveau mecanisme de telesubstitution du brome. *Tetrahedron* **1986**, *42*, 2253-2262.

(18) Li, Y.; Zheng, C.; Jiang, Z.-J.; Tang, J.; Tang, B.; Gao, Z., Potassium tert-butoxide promoted regioselective deuteration of pyridines. *Chem. Commun.* **2022**, *58*, 3497-3500.

(19) In Figure 4, the discrepancy between $R_{\mathrm{C4-N}}$ and the BTE value appears to be rather large, but it must be kept in mind that $R_{\mathrm{C4-N}}$ is not a simple bond distance, as in all other cases. $R_{\mathrm{C4-N}}$ is a cross-ring distance rather a distance between bonded atoms and the discrepancy needs to be considered in this context.
